# Are activation barriers of 50–70 kcal mol^−1^ accessible for transformations in organic synthesis in solution?†

**DOI:** 10.1039/d4sc08243e

**Published:** 2025-02-24

**Authors:** Ruslan R. Shaydullin, Alexey S. Galushko, Valentina V. Ilyushenkova, Yulia S. Vlasova, Valentine P. Ananikov

**Affiliations:** a Zelinsky Institute of Organic Chemistry, Russian Academy of Sciences Moscow 119991 Russia val@ioc.ac.ru https://AnanikovLab.ru

## Abstract

High-temperature organic chemistry represents a transformative approach for accessing reaction pathways previously considered unattainable under conventional conditions. This study focuses on a high-temperature synthesis as a powerful method for performing solution-phase organic reactions at temperatures up to 500 °C. Using the isomerization of *N*-substituted pyrazoles as a model reaction, we demonstrate the ability to overcome activation energy barriers of 50–70 kcal mol^−1^, achieving product yields up to 50% within reaction times as short as five minutes. The methodology is environmentally friendly, leveraging standard glass capillaries and *p*-xylene as a solvent. The significance of high-temperature synthesis lies in its simplicity, efficiency, and ability to address the limitations of traditional methods in solution chemistry. Kinetic studies and DFT calculations validate the experimental findings and provide insights into the reaction mechanism. The method holds broad appeal due to its potential to access diverse compounds relevant to pharmaceuticals, agrochemicals, and materials science. By expanding the scope of accessible reactions, this exploration of experimental possibilities opens a new frontier in synthetic chemistry, enabling the exploration of previously inaccessible transformations. This study establishes a new direction for further innovations in organic synthesis, fostering advancements in both fundamental research and practical applications.

## Abstract 2

High-temperature organic chemistry represents a transformative approach for accessing reaction pathways previously deemed unattainable under conventional conditions.

## Graphical abstract

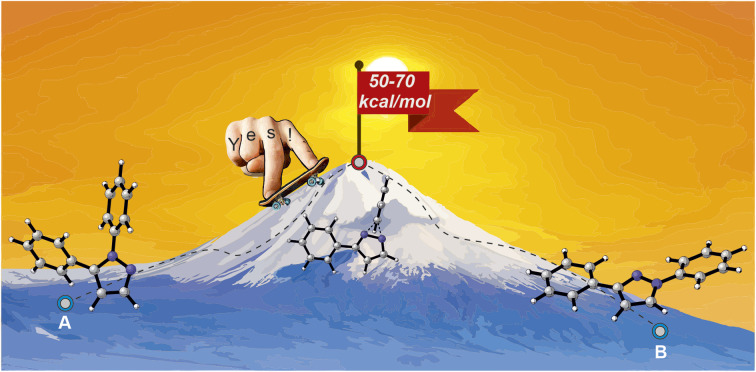

## Introduction

Organic reactions serve as a key technology for the modern society, ensuring applications in diverse fields such as medicine, materials science, and agriculture.^1–5^ These reactions govern the synthesis of life-saving drugs, enabling the development of treatments for numerous diseases,^6–9^ while also supporting the production of monomers and polymers that form the backbone of materials used in everyday life, from packaging to advanced electronics.^10–13^ Agrochemicals, including herbicides and pesticides, rely heavily on innovative organic synthetic processes to enhance crop yields and ensure global food security.^14–16^ Additionally, organic chemistry facilitates the creation of dyes, pigments, and display materials, broadening its impact across numerous industries.^17–19^ Fine chemical synthesis, in particular, is pivotal, enabling the production of tailored molecules that power advancements in pharmaceuticals and beyond.^20–22^

The dominance of solution-phase reactions in organic synthesis is unparalleled, offering a versatile platform for controlling solubility, solvent effects, and reaction dynamics through solvent–solute interactions.^23–25^ Unlike gas-phase chemistry, which is largely limited to small molecules, solution-based methods allow for an expansive range of molecular transformations. Solvents are critical to fine chemical synthesis, providing a medium that enhances reactivity, selectivity, and scalability.^26–28^ Tunability of solvents enables chemists to explore complex reaction landscapes and create an unprecedented diversity of molecular architectures, positioning their role as a central component in synthetic strategies.^29,30^

Despite its versatility, solution-based organic chemistry is typically constrained to reaction temperatures below 200 °C, with only some exceptional cases extending up to 250–300 °C. Traditional approaches, such as microwave-assisted synthesis and closed-vessel techniques, provide a means to achieve elevated temperatures, but their application remains limited for the ranges above. High-temperature solution-phase reactions are often subject to preconceptions in research practice, limiting their potential for developing new chemical methodologies. Of course, high-temperature gas phase reactions (pyrolysis and other techniques) are well-known, but the conditions are principally different as compared to those of solution state chemistry.

**Fig. 1:**
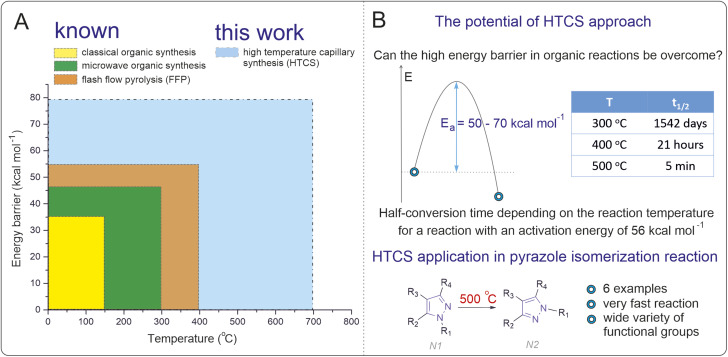
(A) Range of temperatures and energy barriers for carrying out known methods of organic synthesis and the method in this work (the energy barrier is considered high enough at a given temperature if the half-reaction time is more than 24 hours). (B) Half-conversion time depending on the reaction temperature for a reaction with an activation energy of 56 kcal mol^−1^. Specially selected conditions in this work allowed previously inaccessible energy barriers for rearrangement of pyrazoles to be overcome upon heating.

The thermodynamic limitations of conventional solution-phase reactions (<250 °C) impose a restriction for accessing activation energies greater than 40 kcal mol^−1^ (Fig. 1A). Within these constraints prevailing for decades in organic synthesis, the majority of chemical reactions with practical utility in a standard temperature range have already been explored. High-temperature solution-phase reactions offer a promising avenue to overcome this stumbling block, unlocking new synthetic possibilities. By enabling the exploration of transformations with high activation energies, the scope of modern organic synthesis could be significantly expanded,^31–41^ providing access to previously unattainable chemical transformations and molecular frameworks. The isomerization of *N*-substituted pyrazoles represents a suitable model reaction for exploring high-temperature solution-phase chemistry. Pyrazoles play a vital role in medicinal chemistry, agrochemicals, and materials science, serving as core structures in drugs such as celecoxib and other bioactive compounds.^42–46^ However, their isomerization is thermodynamically and kinetically challenging under conventional conditions due to high activation barriers (“forbidden” reaction).^47–55^ Developing a methodology for direct isomerization of pyrazoles would offer a practical advantage, circumventing the need for separate synthetic pathways to access different isomers and expanding the utility of these valuable scaffolds in applied chemistry.

In the present study, we probe a high-temperature synthesis method to overcome the activation barrier limitation in solution-phase organic chemistry. Using *N*-substituted pyrazole isomerization as a model reaction, we demonstrated the ability to perform reactions at temperatures up to 500 °C. Through computational and kinetic studies, we confirmed that this approach enables access to reactions with activation barriers exceeding 50 kcal mol^−1^. The methodology was validated for its applicability and efficiency, achieving good reaction yields in significantly reduced reaction times (Fig. 1B).

The importance of the present study lies in demonstrating the principal feasibility of overcoming activation barriers of 50–70 kcal mol^−1^ in solution-phase organic synthesis. By employing high-temperature synthesis in practice, we addressed the fundamental question of whether such barriers can be surpassed, providing a practical methodology to unlock new chemical transformations that were previously inaccessible under conventional conditions.

## Results and discussion

### Mechanistic analysis of the reaction by DFT modeling

**Fig. 2:**
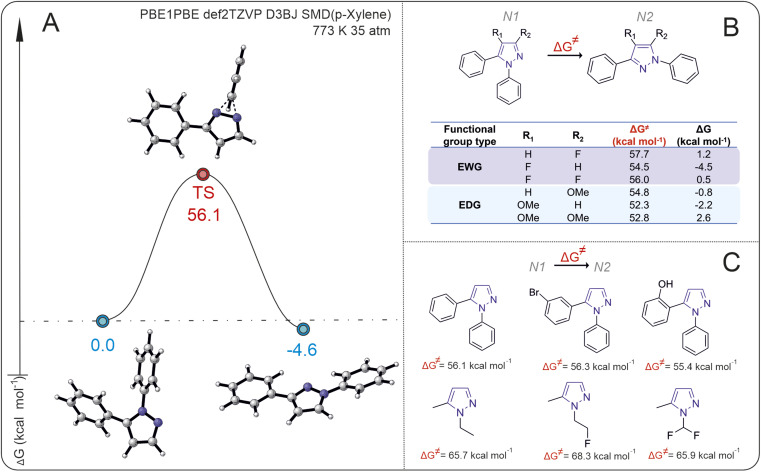
(A) Isomerization of the 1,5-diphenylpyrazole free energy profile. (B) The effect of substituents (–OMe and –F) on reaction Gibbs energies and activation Gibbs energies. (C) Activation Gibbs energies for isomerization of the *N*_1_-substituted pyrazoles.

In the first stage of our research, we analyzed the energy barriers of rearrangement for a series of *N*_1_- to *N*_2_-diarylpyrazoles, using 1,5-diphenylpyrazoles as a model substrate. The isomerization of *N*_1_ pyrazole shows a high activation Gibbs energy, approximately 56 kcal mol^−1^, with a small thermodynamic energy difference between the starting compound and the product (Δ*G* < 5 kcal mol^−1^) (Fig. 2A).

The introduction of electron-donating groups EDG and electron-withdrawing groups EWG at the *R*_1_ or *R*_2_ positions of 1,5-diphenylpyrasole (*N*_1_) does not lead to significant changes in the Gibbs activation energy of the reaction (the difference is less than 5 kcal mol^−1^) (Fig. 2B). Interestingly, the introduction of an EWG (–F) or an EDG (–OMe) into the *R*_1_ position of *N*_1_ pyrazole results in *N*_2_ pyrazole becoming energetically favorable, whereas when the EWG or EDG is in the *R*_1_ and *R*_2_ positions simultaneously, *N*_1_ pyrazole becomes more favorable. The introduction of an EWG (–F) or EDG (–OMe) at the *R*_2_ position results in a thermodynamic preference for the *N*_2_ (–OMe) or *N*_1_ (–F) product correspondingly. Despite the small difference in energies between the initial substrates and isomerization products (which, strictly speaking, is close to the error of computational methods), the appearance of even a small energy change between the isomers would allow observation of the reaction (*i.e.*, the second isomer may form at least in small amounts).

It can also be assumed that owing to such a small difference in the calculated energies of the state of the substances, provided that the high energy barrier is overcome, an equilibrium mixture of the starting substance and the product, which can be registered by standard physicochemical methods of analysis, should be observed in the system. It is unlikely that full conversion of one isomer to another isomer would take place, but formation of the second isomer should be observable if it is possible to overcome the activation barrier.

To understand how large the energy barrier is for isomerization of other pyrazoles with different substituents both on the *N*_1_ atoms of pyrazole and on the substituents in *N*_2_, the activation Gibbs energies for different pyrazole substrates were calculated (Fig. 2C). The activation Gibbs energies of pyrazoles with aryl substituents at the *N*_1_ position are significantly lower than those of pyrazoles with alkyl or alkylfluoro substituents. For 1-(2-fluoroethyl)-3-methyl-1*H*-pyrazole, the activation Gibbs energy is 68.3 kcal mol^−1^, whereas for 3-(1-phenyl-1*H*-pyrazol-2-yl)phenol, it is 55.4 kcal mol^−1^.

On the basis of the calculated data of the Gibbs energy change for the transition state, the temperature at which the energy barrier can be overcome is estimated *via*eqn (1).1
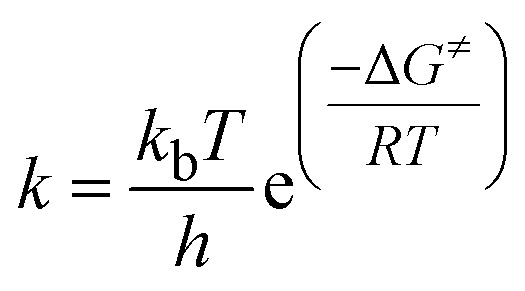


A good indicator of the time required for a reaction is the transformation time *t*_1/*n*_, which for the first-order reaction A → B is *t*_1/*n*_ = ln(*n*)/*k*, where *n* is a number indicating how many times the concentration of A has decreased relative to the initial concentration. In the special case when *n* = 2, the time *t* = *t*_1/2_ is the half-reaction time. Fig. 1B shows the *t*_1/2_ data for different temperatures. Interestingly, the reaction can be carried out in a short time under conditions in which a high energy barrier is obtained. The calculated data suggest that the optimal isomerization temperature is approximately 500 °C, which is a nontrivial task in regular organic syntheses. Therefore, a search for techniques for synthesis at high temperatures in organic reactions was carried out.

### Experimental realization of high temperature transformation

For ease of use and the possibility of easy reproduction of the setup in other laboratories without the need for special expensive equipment, we implemented a method of organic synthesis at high temperatures and pressures in sealed capillaries and called this method high-temperature capillary synthesis (HTCS). Glass capillaries are commonly available, so we believe that this synthesis method is convenient and easily reproducible in laboratories. Owing to capillary effects, thin tubes with a small-bore diameter in sealed form can withstand high pressures up to 35 atm arising during heating. The detailed methodology of synthesis in capillaries is shown in the ESI.†

**Fig. 3:**
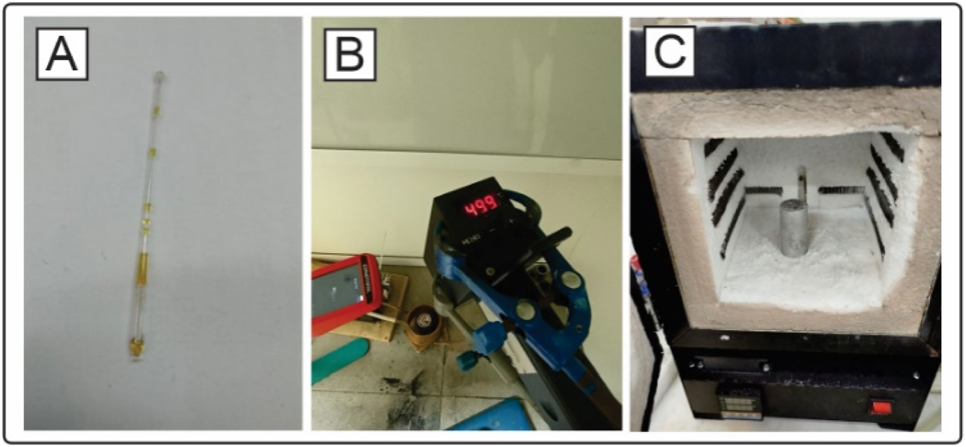
(A) Photo of a capillary with *p*-xylene after heating to 500 °C for 5 min. (B) Photo of an experiment with induction heating. (C) Muffle furnace with maximum heating temperature of 1200 °C. Chamber volume: 3.6 liters.

Since the ratio of the empty volume inside the capillary to the solution-filled volume affects the internal pressure created by the vapors when heated, the effect of the degree of filling of the capillary with solution on the ability of the capillary to withstand the required synthesis conditions was studied. When the capillary was filled to 50% (50 μL free volume to 50 μL *p*-xylene), 5 out of 10 capillaries burst. When the capillary was filled to a 25% volume (25 μL), all 10 capillaries survived the conditions. The capillaries in all the cases were 8 cm long at the sealing sites (230 mm Duran pipettes) (Fig. 3A). To approximately estimate the pressure generated inside the capillary, an experiment was performed to reproduce the synthesis conditions in high-pressure reactors. The pressure determined by the manometer on the reactor after heating *p*-xylene to 500 °C (occupied volume of solution 25%) was 32.3 bar; thus, we can conclude that the glass capillaries in our studies can withstand this pressure. Notably, when heated to a temperature of above 500 °C, the capillary loses the meniscus between the gas and liquid phase. In this case, due to high pressure, the organic substance/solvent may enter a supercritical state. For *p*-xylene, the triple point is at 616.2 ± 0.2 K, 35 ± 2 bar.^56,57^ Considering the measurement error in HTCSs, the solvent may be at a point on the phase diagram close to the supercritical fluid. The supercritical state may be preferable compared to the regular liquid or gas-phase state of the solvent for high-temperature organic syntheses. The supercritical state may result in better mixing of substrates and better heat transfer, which would be difficult to achieve in the case of a gas-phase reaction in a short time.

The systems can be heated in different ways: *via* microwaves, *via* induction heating or in a classic muffle furnace. Inductive heating of materials is extremely fast and provides the best power transfer rates of all heating technologies.^58^ The material to be heated is usually located inside or close to the coil so that heat is not generated by convection over the surface. This method has found wide industrial application for heating large metal objects and workpieces. We assembled an induction heating system with temperature control *via* a laser pyrometer (ESI† and Fig. 3B). The heated element is a graphite cup, in which an aluminum cylinder with holes for capillaries is placed. Good reproducibility has been shown even after 50 heating and cooling cycles (see the ESI† for more details). The isomerization of 1,5-diphenylpyrazole was carried out on this apparatus with an isolated yield of 42% (48% conversion by ^1^H NMR) of the isomerization product, with 10% by ^1^H NMR of the source compound remaining. The structure of the product was confirmed by X-ray analysis (see the ESI†). The setup was experimentally convenient, although the disadvantage of this design is the lack of accurate temperature control directly near the capillaries due to impossibility to introduce a thermocouple (metal thermocouple cannot be placed inside the heater since the thermocouple will be disproportionally heated by induction currents).

Another alternative for carrying out the syntheses is the use of a suitable furnace, which allows the temperature of the experiment to be adjusted with greater precision. The maximum yield of the reaction product after isolation was 45% (50% conversion by ^1^H NMR), while the starting compound 1,5-diphenylpyrazole remained in the equilibrium mixture (^1^H NMR). Despite the energy efficiency of induction heating, the simple muffle furnace design was more convenient for high-temperature organic synthesis (Fig. 3C). Further syntheses were performed *via* a muffle furnace with glass fiber thermal insulation. This type of insulation allows the reduction of negative effects during a plausible capillary fracture.

**Fig. 4:**
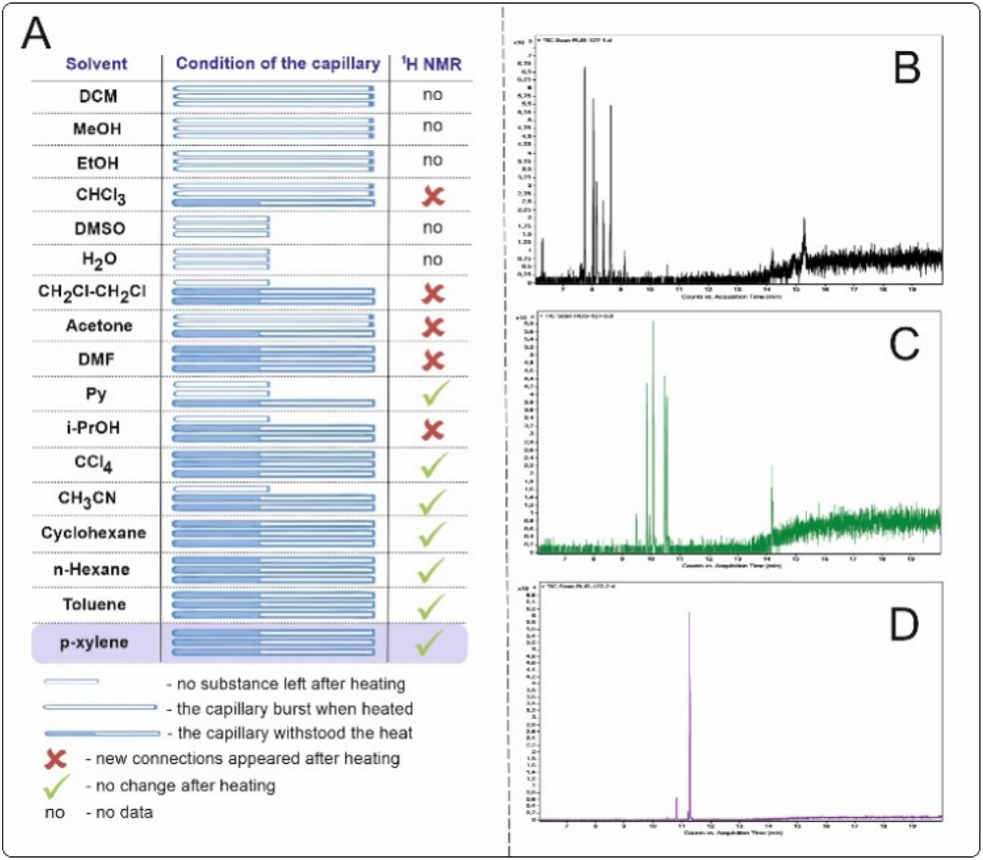
(A) Varying solvents for high-temperature organic synthesis. The capillaries were filled with 25 μL of solvent and sealed. An experiment was carried out, and the capillary was opened and washed with 500 μL of CDCl_3_ for ^1^H NMR spectrum registration. (B) Gas chromatography of the reaction mixture with cyclohexane after the experiment. (C) Gas chromatography of the reaction mixture with toluene after the experiment. (D) Gas chromatography of the reaction mixture with *p*-xylene after the experiment.

Studied solvents, when heated to 500 °C for 15 min, led to the formation of significant amounts of byproducts, as recorded by ^1^H NMR and GC-MS. All experiments were performed on three different filled capillaries for the set of statistical data of solvent behavior (Fig. 4A). Solvents such as water, dimethylsulfoxide (DMSO), and pyridine (Py) caused the capillaries to rupture. In the case of DMSO, the Py capillaries ruptured, most likely due to the pressure increase caused by outgassing from side processes occurring at such high temperatures.

The most suitable solvents for high-temperature syntheses are considered to be aromatic or saturated hydrocarbons since the formation of the least number of byproducts that could interfere with the main reaction is expected. Examination of the reaction mixtures after the experiment by gas chromatography coupled with mass spectrometry (GC–MS) for cyclohexane, toluene and *p*-xylene (Fig. 4B–D) revealed that *p*-xylene was the most preferred solvent for high-temperature synthesis. A dozen new compounds are observed *via* GC–MS in the case of cyclohexane and toluene. The reaction temperature suggests that radical reactions may have occurred. In the case of *p*-xylene, two main byproducts observed on the GC-MS are formed. This is most likely 1,2-bis(4-methylphenyl)ethane or its isomers (see the ESI† for more details).

The solvent should be used to achieve high yields, and the solvent-free system may not be optimal. In experiments with 3 mg of 1,5-diphenylpyrazole and 25 μL of *p*-xylene, conversion of the reagent to the product (^1^H NMR) was 29.1%, and the isolated yield was 28.8% after 3 min of heating at 500 °C. When 3 mg of 1,5-diphenylpyrazole was heated in the absence of solvent, the conversion was 59.9%, with an isolated yield of only 4.3%. These findings suggest that in the absence of a solvent, side reactions such as bimolecular reactions of pyrazoles interacting with each other may occur, resulting in high conversion in the reaction mixture and a low yield of the isomerization reaction target substance. A photograph of the capillaries after heating to 500 °C is presented (see the ESI† for more details). The color of the capillary in which the experiment was conducted without solvent noticeably changed, and the formation of soot (carbonization products) was observed. In the capillary with *p*-xylene, visual changes are also observed after the reaction (Fig. 3A). Therefore, initially, the capillary was translucent, and after the reaction, the solution became yellow in color. The use of a solvent is essential to achieve a better yield of the target reaction product.

Pyrazoles are reactive compounds; thus, isomerization and decomposition reactions are reported in the literature.^59,60^ For this reason, the stability of pyrazoles when synthesized in capillaries and at high temperatures is unclear. Thermogravimetric analysis (TGA) was carried out to evaluate the stability of the pyrazoles when heated to the boiling point. The behavior of the samples in both air and argon atmospheres was studied, and it was found that there was no significant difference between them. Two endothermic peaks are recorded *via* differential scanning calorimetry (DSC). The first one is without mass loss, probably melting. The second peak corresponds to the main degradation or vaporization of the substance. The second process is one-step, and if it is degrading, it is in a rather narrow temperature range. The beginning of mass loss (5%) occurred at 182 °C. Pyrazole does not oxidize in air, and there are no other reactions that can take place at temperatures up to 180 °C. Consequently, 1,5-diphenylpyrazoles are stable when heated to their boiling point.

### High-temperature rearrangements

The main variable parameters of the reaction are the temperature and time of the experiment. Optimization of the conditions was carried out, and the optimal temperature of the reaction was 500 °C (Table 1). Notably, the reaction yield may depend on the concentration of the substance. Capillaries with 2 mg of substance in 25 μL of *p*-xylene were used for optimization. In each experiment, 3 capillaries were used to check for reproducibility. The data presented in Table 1 are the average of the three results. When the synthesis time is small, controlling the reaction time and heat transfer is difficult. When the capillary is placed in a muffle furnace heated to a given temperature, a certain amount of time elapses from the time the capillary is heated from room temperature to a temperature that allows the energy barrier of the reaction to be overcome. However, this time is less than 2 min, since in the experiment at 500 °C for 2 min, the yield of the target product was already observed.

**Table 1:** Optimization of high-temperature synthesis conditions

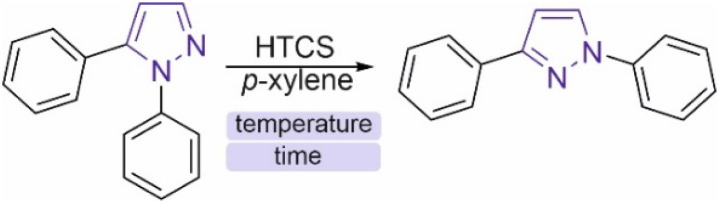			
Temperature (°C)	Time (min)	Yield (%)	Source compound (%)
520	5	1	70
500	2	8	57
500	10	54(50^a^)	7
480	6	14	59
480	20	47	16
460	10	12	51
460	35	23	8
440	25	27	30
420	90	26	11

a Yield of the isolated substance. The reaction mixture was set to 10 mg.

Such a high temperature of synthesis allows the reactions to be carried out in a short time, such as 5 min, which is an advantage of this technique, but it also provides an opportunity for other reactions that are off-target and result in a low conversion of the substrate to the target product. High-temperature synthesis, in this case, is a necessary criterion for overcoming the high energy barrier, and temperature reduction could be achieved by carrying out the reaction in catalytic mode. The search for substances for catalytic activity did not lead to a positive result (examples of components investigated for catalytic activity are presented in the ESI†). It was expected that Cu compounds could have catalytic activity because of their ability to coordinate pyrazoles at the nitrogen atom. A search among the Cu^0^, Cu^1+^, and Cu^2+^ compounds did not lead to a positive result.

With the introduction of Pd, Pt, Au, *etc.*, the amount of organic impurity compounds in the mixture, as determined by ^1^H NMR, noticeably decreased. This is most likely because these metals lead to the complete decomposition of organic compounds under such conditions. The absence of a catalyst in this reaction can be seen as an advantage, as it results in no metal contamination, and no posttreatment is required to remove this metal from the target product.

The observed stability of the product and the starting compound at such high temperatures is interesting. The amount of product does not change monotonically as a function of reaction time, and there is a certain maximum after which the product content in the reaction mixture decreases, *i.e.*, the curve has a dome-shaped appearance. The GC–MS data of the reaction mixtures (Fig. S8†), which were obtained at 500 °C in steps of 1, 5, 10 and 15 min, are presented. The maximum content of the isomerization reaction product is observed at 5 min, after which its content decreases. This may be due to both the instability of the reaction product and the occurrence of side reactions with the compound, *i.e.*, the source compound reacts, and a product is formed that may subsequently react in other reactions or decompose. The source compound is likely to be affected in the same way.

### Reaction kinetics at high temperatures

**Fig. 5:**
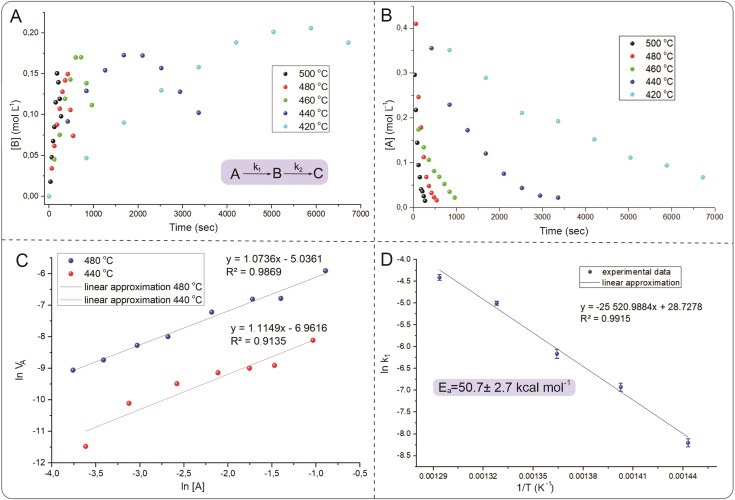
(A) Kinetic curves of changes in the concentration of 1,3-diphenylpyrazole, the product of the isomerization reaction. (B) Kinetic curves of changes in the concentration of the starting compound 1,5-diphenylpyrazole. (C) Measurement of the reaction order from the results of kinetic measurements of 1,5-diphenylpyrazole at 440 °C and 480 °C. (D) Data from the experimental determination of the activation energy for the isomerization reaction of pyrazoles. Kinetic data were measured using the ^1^H NMR method with 2,5-dimethylfuran as an internal standard.

To understand the reaction mechanism and search for optimal parameters for the synthesis and determination of activation energies, kinetic curves of the isomerization of 1,5-diphenylpyrazole into 1,3-diphenylpyrazole in *p*-xylene were experimentally constructed at temperatures ranging from 420 °C to 500 °C with a step of 20 °C (Fig. 5). To the best of our knowledge, this work is the first to use such high-temperature measurements of organic reactions studied. Known syntheses of organic substances at temperatures above 350 °C usually take place in closed vessels or FVP-type systems, which do not allow kinetics measurements or are very difficult to perform. The HTCS method allows such an experiment. The resulting time dependence of the concentration of the isomerization product of 1,5-diphenylpyrazole into 1,3-diphenylpyrazole is described by a dome shape, which corresponds to the kinetics of the sequential reaction.

There is a maximum point of the formed product, after which its concentration decreases with reaction time, which is explained by possible side reactions with 1,3-diphenylpyrazole. Moreover, the source compound (1,5-diphenylpyrazole) is also subject to side reactions, since at temperatures above the boiling point, radical reactions usually begin to predominate. These reactions can include carbonization, degradation, decomposition or interaction with a solvent to form a new compound. It was not possible to isolate individual compounds from the side reactions, but as noted earlier, a change in the color of the solution after the experiment was observed. The measured concentrations of 1,5-diphenylpyrazole and the source compound, over time are well described by first-order kinetics. To describe the kinetic dependencies, we considered this reaction in a model version, as shown in formula S1 (ESI†).

**Fig. 6:**
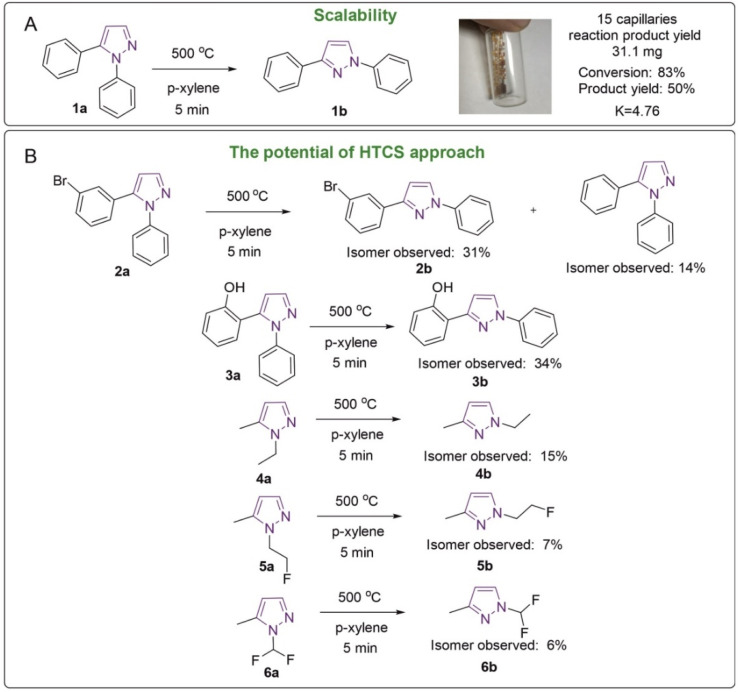
High temperature reactions: (A) the general reaction scheme and scaling and (B) reaction examples.

Measurements of the changes in the concentrations of the starting compound and the reaction product allow us to find the rate constants *k*_1_, and *k*_2_. According to the calculated data presented earlier, the Gibbs energy difference between the starting compound and the product is small and is on the order of 4.6 kcal mol^−1^, which corresponds to an equilibrium constant of 20 (*T* = 773 K). Therefore, it was expected that this reaction mixture would exhibit equilibrium with similar concentrations of the product and initial compound. However, the experimental data show that the equilibrium is strongly shifted toward the product. The reason for this difference between the experimental data and the calculations remains unclear, and the study of reaction mechanisms at high temperatures is a new field to date. The initial pyrazole is involved in other reactions, which affects the determination of the activation energy and reaction order. Fig. 6 shows that the yield of the reaction at temperatures below 500 °C results in more products being formed at the peak point than at 500 °C. This difference in synthesis temperature did not significantly increase the product yield (50% *versus* 60%), but performing the reaction at 500 °C noticeably accelerated the synthesis process (5 min instead of 35 min). The balance of synthesis time and yield of the reaction product at 500 °C is optimal from our point of view.

The measurement of the reaction rate constants at different temperatures allowed us to find the experimental activation energy *via* the Arrhenius equation. The measured activation energy was 50.7 ± 2.7 kcal mol^−1^, which is comparable to the values obtained *via* DFT calculations (57.2 kcal mol^−1^). This activation energy was determined *via* a simplified model, which does not consider side reactions that could occur with the source compound. Therefore, this value of the activation energy is a composite value, whereas the actual value for a particular process will be somewhat different. Nevertheless, the experimentally measured activation energy illustrates the large energy barrier of the process, which is difficult to overcome with traditional organic chemistry methods. Without performing DFT calculations, such a high activation barrier is very difficult to assume and is based only on structural changes. This is probably why this type of reaction is not widely reported in the literature, and the high energy barrier obviously limits the study of such transitions. The next step after optimization of the conditions and description of the kinetic dependences was to study the generality of the observed process for the example of other pyrazoles with other substituents.

### Experimental observation of overcoming a large activation barrier for substrate scope

To further address the possibility of overcoming large activation barriers, first, scaling to a larger amount of product was carried out (Fig. 6A). In this study, the isomerization reaction of 1,5-diphenylpyrazole was set up in 15 sealed capillaries, loading 4–5 mg per capillary in 25 μL of *p*-xylene. The fifteen capillaries were placed in a tubular oven, the synthesis itself took 5 min. The mass of the isolated product was 31.1 mg, and the yield was 50%, which is not different from the isolated yield recorded earlier. Notably, equilibrium of the starting compound and product was observed in the reaction mixture. The experimental equilibrium constant was 4.76 at 500 °C. Heating 1,3-diphenylpyrazole at 500 °C for 10 min led to the formation of small amounts of 1,5-diphenylpyrazole, which shows the reversibility of this reaction.

The pyrazole isomerization reaction, in addition to the model 1,5-diphenylpyrazole, was tested for other compounds **2a–6a** (Fig. 6B). In all the studied cases the formation of the second isomer was observed. The amounts of the compounds formed were in the range of 6–34% (Fig. 6B). It should be pointed out that a high degree of conversion should not be expected in all the cases, since the isomers may have rather similar reaction energies, and relative stabilities of *N*_1_/*N*_2_ forms may change (see the Mechanistic analysis of the reaction by DFT modeling section above). The principal outcome of these experiments is the demonstration that high activation barriers were successfully overcome for a number of examples (Fig. 6).

It should be noted that not all functional groups can be suitable for this method of synthesis; for example, with furan, 5-(2-furanyl)-1-phenyl-1*H*-pyrazole, the observed ^1^H NMR yield of the product was 0%, which can be explained by the instability of the compound, which is subjected to other reactions, including those leading to polymerization. Interestingly, in the reactions with halogen derivatives such as Br (2a) significant formation of product 1a was observed. This can be explained by the fact that the C–Br bond is unstable and susceptible to breakage at such temperatures. Most likely, a high-temperature homolytic bond breaks after collision with a solvent molecule that accepts the solvent hydrogen.

To study the mechanism of the formation of product 1a from 2a, an ESI-HRMS experiment with the deuterated solvent *p*-xylene-d_10_ was carried out. When *p*-xylene-d_10_ is present in the reaction medium, only deuterated compounds 1a are formed, indicating that D or H are taken from the solvent rather than from the source compound or isomerization product. This is due to the different collision frequencies of the molecules of the investigated compounds with the solvent and with their own substance molecules. Since the kinetic curves indicate a monomolecular process, the probability of the collision of pyrazole molecules with themselves in the reaction medium is low, and the exchange of hydrogen with deuterium can take place in several stages. The exchange of deuterium up to 5 of deuterium atoms has been observed. In the future, this methodology will allow the application of high-temperature organic chemistry in capillaries for deuterium exchange processes and the creation of isotopically labeled compounds.

An example of 1,5-diphenylpyrazole with synthetically useful conversion (83%) and good isolated yields (50%) suggests potential practical application for the isomerization reaction studied. This may help accessing another isomer, which may not be equally well accessible from the synthesis. The structure of pyrazoles is found in many drugs, for example one commercially available example is celecoxib. Celecoxib, which is sold under the brand name Celebrex, among others, is a COX-2 inhibitor and nonsteroidal anti-inflammatory drug (NSAID). It is used to treat pain and inflammation in osteoarthritis, acute pain in adults, rheumatoid arthritis, psoriatic arthritis, ankylosing spondylitis and other diseases.^61,62^

## Conclusions

This study demonstrates the feasibility of preparative organic synthesis at 500 °C, marking a significant advancement in reaction-extended solution-phase chemistry. Using *p*-xylene as the optimal solvent, the method showcases excellent scalability and the ability of capillaries to withstand medium pressures, enabling rapid and efficient reactions. As exemplified by the isomerization of pyrazoles, this approach achieves reaction completion in as little as five minutes. The experimental activation energy of 50.7 ± 2.7 kcal mol^−1^, aligns well with DFT calculations, reinforcing the validity of the method's conditions.

The development of high-temperature organic chemistry unlocks new opportunities for synthesizing compounds previously inaccessible due to synthetic technique limitations and the constraints of conventional methodologies. The HTCS method offers considerable potential for expanding the scope of target compounds. While not all substances may be suitable for high-temperature transformations and yields may vary, this approach can still be advantageous. Even with modest yields, the ability to synthesize target compounds in a single step from simple substrates often surpasses the overall efficiency of multi-step classical methods, making HTCS potentially a more economically viable solution.

The exploration of high-temperature synthesis represents an exciting and underexplored frontier in organic chemistry. Expanding knowledge in this area is expected to lead to the discovery of novel reactions, facilitating advancements in both fundamental research and practical applications in synthetic chemistry.

### Limitations

While the high-temperature method demonstrates significant promise, it is not without its limitations. The methodology may not be universally applicable to all reaction types or substrates. Certain functional groups and sensitive compounds may degrade or undergo side reactions under the high temperature conditions required for these transformations. Additionally, while the study showcases the successful isomerization of *N*-substituted pyrazoles, further investigations are necessary to adapt and optimize this approach for a broader range of reactions and complex substrates.

Despite these limitations, the opportunities provided by this method are undeniably exciting. The described opportunity provides a gateway to explore previously inaccessible reaction pathways, offering new possibilities for synthetic chemistry. Continued research will be crucial for refining the technique, expanding its scope, and addressing its constraints, potentially unlocking its full potential across diverse fields of application.

The energy of the C–C bond is estimated to be around 87 kcal mol^−1^ and other CC bonds are typically stronger,^63,64^ so overcoming barriers of the order of 70 kcal mol^−1^ is possible and should not lead to the breakage of the carbon skeleton. Nevertheless, attempts to overcome large barriers may reach a limitation due to the stability of organic molecules towards partial degradation. The topic should be studied further in more detail in future studies.

## Data availability

The data supporting this article have been uploaded as part of the ESI.†

## Author contributions

Ruslan R. Shaydullin: formal analysis; methodology; visualization; writing – original draft; writing – review & editing. Alexey S. Galushko: formal analysis; methodology; visualization; writing – original draft; writing – review & editing. Valentina V. Ilyushenkova: methodology; formal analysis; visualization; writing – review & editing. Yulia S. Vlasova: formal analysis; methodology; investigation; writing – review & editing. Valentine P. Ananikov: idea; development; supervision; conceptualization; investigation; visualization; writing – original draft; writing – review & editing.

## Conflicts of interest

There are no conflicts of interest to declare.

## Supplementary Material

SC-OLF-D4SC08243E-s001

SC-OLF-D4SC08243E-s002
