# Supplementary material for: Are activation barriers of 50–70 kcal mol−1 accessible for transformations in organic synthesis in solution?
Source: Chem Sci. 2025 Feb 24;16(12):5289–98. doi: 10.1039/d4sc08243e (PMC11848743; doi:10.1039/d4sc08243e)
Supplement: SC-016-D4SC08243E-s001 [file SC-016-D4SC08243E-s001.pdf]

Supplementary Information (SI) for Chemical Science.
This journal is © The Royal Society of Chemistry 2025

Supporting information for the article

Are activation barriers of 50-70 kcal mol^{-1} accessible for transformations in organic synthesis in solution?

Ruslan R. Shaydullin, Alexey S. Galushko, Valentina V. Ilyushenkova, Yulia S. Vlasova, Valentine P. Ananikov*

Zelinsky Institute of Organic Chemistry, Russian Academy of Sciences, Moscow 119991, Russia

(*) E-mail: val@ioc.ac.ru; https://AnanikovLab.ru

Contents

General experimental information S2
Theoretical calculations S4
Synthetic method S16
Catalyst search S22
Kinetics measurement S23
Mechanistic study S30
Preparation of pyrazoles and isomerization reactions S32
^{1}H NMR, ^{13}C NMR spectra and HRMS S36
X-ray crystallographic data and refinement details. S66
References S74

General experimental information

General procedures.

All reagents were purchased and used as received except when indicated. Chemical shifts (δ) are given in ppm downfield from Me_4Si and refer to the internal standard to the residual solvent (unless indicated) $CDCl_3$: (δ = 7.26 for ^1H and 77.00 for ^{13}C). The coupling constants, *J*, are reported in Hertz (Hz). TLC was carried out on SiO_2, and the spots were located with UV light. Flash chromatography was carried out on SiO_2. The organic extracts were dried over anhydrous Na_2SO_4 during the reactions. Evaporation of the solvents was accomplished with a rotatory evaporator.

Caution for high temperature reactions.

We urge researchers to be careful when conducting any high temperature experiments and ensure that special precautions be taken. Glass capillaries can explode at high pressures and temperatures, producing dangerous sharp fragments. The necessary precautions should be taken and appropriately configured laboratory environment should be arranged.

Computational details.

Geometry optimization for pyrazoles and vibrational frequency calculations were performed via the PBE1PBE (PBE0)[1,2] hybrid functional and def2TZVP[3,4] basis set, the D3 version of Grimme's dispersion with Becke–Johnson damping (GD3BJ)[5,6] in Gaussian16[7]. The SMD model (solvation model based on the charge density of solute molecules) was used as a continuum model of the solvent (*p*-xylene) media.[8] For all the structures, the thermodynamic parameters were calculated at 298.15 K and 1 atm. For all structures the calculations were also carried out at 773 K and 35 atm. This temperature and pressure are approximately the same as the conditions for synthesis. Stationary points were characterized as minima by evaluating the elements of the Hessian matrix. All the observed transition states had one negative vibrational mode corresponding to the reaction path. Visualization and analysis of optimized structures were performed using CYLview program.[9]

ESI-HRMS study.

The samples for the ESI-TOF-HRMS experiments were prepared in 1.8 mL glass vials with screwtop caps fitted with Teflon-lined septa (Agilent Technologies). High-resolution mass spectra (HRMS) were recorded on a Bruker maXis q-TOF (tandem quadrupole/time-of-flight mass analyzer) mass spectrometer equipped with an electrospray ionization (ESI) source. The *m/z* scanning range was 50–3000. The measurements were carried out in positive ion mode (+) (ground spray needle, –4500 V high-voltage capillary; 500 V HV end plate) and in negative ion mode (–) (ground spray needle, +4000 V high-voltage capillary; –500 V HV end plate offset). External calibration of the mass scale was carried out via the low-concentration calibration solution "tuning mix" (Agilent Technologies). The samples were injected via a 500 μL Hamilton RN 1750 syringe (Switzerland). The flow rate during injection was controlled with a syringe pump (3 μL/min).

Nitrogen was used as the nebulizer gas (1.0 bar) and dry gas (4.0 L/min, 200 °C). The data were processed via Bruker Data Analysis 4.0 software.

NMR study.

NMR spectra were recorded via Bruker Fourier 300HD spectrometers operating at 300.1 MHz for ^{1}H and 76 MHz for ^{13}C. The ^{1}H and ^{13}C NMR chemical shifts are reported relative to the solvent signals as internal standards: 2.50 ppm/39.5 ppm for DMSO-d_6 and 7.26 ppm/77.2 ppm for $CDCl_3$. All the measurements were performed at room temperature.

X-ray crystallographic data and refinement details.

X-ray diffraction data were collected at 100 K on a four-circle Rigaku Synergy S diffractometer equipped with a HyPix6000HE area detector (kappa geometry, shutterless ω-scan technique) using graphite monochromatized Cu K_a-radiation. The intensity data were integrated and corrected for absorption and decay via the CrysAlisPro program[10]. The structure was solved via direct methods via SHELXT[11] and refined on F^2 via SHELXL-2018[12] in the OLEX2 program.[13] All nonhydrogen atoms were refined with individual anisotropic displacement parameters. All hydrogen atoms were placed in ideal calculated positions and refined as riding atoms with relatively isotropic displacement parameters. The Mercury program suite[14] was used for molecular graphics. The structures have been deposited at the Cambridge Crystallographic Data Center with the reference CCDC numbers 2419386.

GC-MC analysis.

The system consists of a gas chromatograph (Agilent Technologies 7890B) coupled with a quadrupole mass spectrometer equipped with an electron ionization source (Agilent Technologies 5977A MSD). NIST 2017 mass spectral library software, version 2.3 (Gaiticsburg, Pennsylvania, USA), was used to collect and process the data.

GC-MS analysis was performed on a HP Agilent 5 ms capillary column with a length of 30 m, an I.D. of 0.25 mm, and df of 0. 25 μm, carrier gas - helium, injector temperature - 270 °C, initial chromatograph oven temperature - 60 °C, heating rate 10 °C/min to 300 °C, followed by isotherm for 5 min; MS parameters: electron ionization at 70 eV, source temperature - 230 °C.

Sample preparation for GC/MS analysis. Two microliters of the upper reaction layer was transferred to a vial and diluted with 1 ml of ACN (HPLC). A 1 μl solution was injected into the GC/MS system.

TGA analysis.

Instrument used for TGA analysis - DTG-60H "Shimadzu", heating rate 10 °C/min, temperature range 30–450 (850) °C

Theoretical calculations

XYZ coordinates, total energy (E), enthalpy (H) and Gibbs free energy (G) values for optimized structures

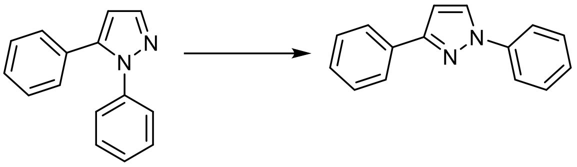

	ΔE (ΔE≠), kcal/mol	ΔH (ΔH≠), kcal/mol	ΔG (ΔG≠), kcal/mol	K = exp(-ΔG/RT)	k, s^{-1}	$t_{1/2}$, s
298.15 K; 1 atm	-3.3 (57.2)	-3.3 (54.8)	-3.8 (55.2)			
773 K; 35 atm (experiment conditions)	-3.3 (57.2)	-3.2 (54.5)	-4.6 (56.1)	20.2	0.0022	315

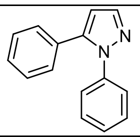			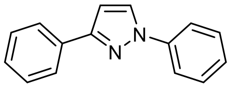		
	**298.15 K; 1 atm**	**773 K; 35 atm**		**298.15 K; 1 atm**	**773 K; 35 atm**
Total Energy	-687.790550 Eh	-687.705926 Eh	Total Energy	-687.795774 Eh	-687.795774 Eh
Total Enthalpy	-687.543021 Eh	-687.475879 Eh	Total Enthalpy	-687.548221 Eh	-687.481007 Eh
Total Gibbs free energy	-687.597184 Eh	-687.705926 Eh	Total Gibbs free energy	-687.603197 Eh	-687.713285 Eh
Number of imaginary frequencies	0	0	Number of imaginary frequencies	0	0

C	-4.90381700	1.68831000	-0.13280400	C	-10.92342900	3.30415800	0.88596000
C	-4.40788000	0.40363700	-0.22751900	C	-12.10818300	2.63907200	1.28087700
C	-3.03430200	0.52348300	0.02229500	C	-11.85437200	1.31212100	1.06791100
N	-3.82995200	2.48237700	0.14504000	N	-10.59240200	1.22553300	0.57961800
N	-2.69057700	1.77877400	0.24553200	N	-10.02070000	2.42871300	0.46254400
C	-3.76941700	3.89086600	0.25803300	C	-9.89354700	0.06670100	0.19048200
C	-6.29279500	2.14319600	-0.21303100	C	-10.28790600	-1.18157800	0.65849200
C	-4.44608600	4.69884600	-0.64529500	C	-9.59819600	-2.31316400	0.25520500
C	-3.00587900	4.45059000	1.27328700	C	-8.80389900	0.18124000	-0.66496800
C	-2.93254600	5.82876700	1.38995100	C	-8.11769000	-0.95722500	-1.05181200
C	-3.62283800	6.64510200	0.50485400	C	-8.51134500	-2.20873000	-0.59986500
C	-4.37655300	6.07615400	-0.51132700	H	-7.26898800	-0.86277100	-1.71952900
H	-2.47480400	3.79916000	1.95569200	H	-8.51020500	1.16110100	-1.01607700
H	-2.33611800	6.26648300	2.18213600	H	-7.97186800	-3.09644100	-0.90819900
H	-3.56747400	7.72304700	0.60265400	H	-9.90947800	-3.28324600	0.62524000
H	-4.90635500	6.70693300	-1.21568600	H	-11.11516400	-1.27333300	1.35070700
H	-5.01712300	4.25195400	-1.44879900	C	-10.62852700	4.73693100	0.90652200
C	-6.82761600	3.02740800	0.72472800	C	-11.60433500	5.66056600	1.27916200
C	-7.12005700	1.64859800	-1.22080200	C	-9.36234400	5.20680800	0.55307100
C	-8.44875400	2.03502000	-1.29465900	C	-11.32381400	7.01726800	1.29787800
C	-8.96927600	2.92230700	-0.36421000	C	-9.08423600	6.56211600	0.57221300
C	-8.15443500	3.41466900	0.64595200	C	-10.06290900	7.47449600	0.94463400
H	-9.07806000	1.64540000	-2.08679200	H	-9.84304100	8.53592200	0.95953300
H	-6.70955200	0.96684800	-1.95704000	H	-12.09555700	7.72081100	1.58945400
H	-8.55623500	4.10086100	1.38277400	H	-12.59483400	5.31699200	1.55411200
H	-10.00764700	3.22773900	-0.42367300	H	-8.09524500	6.91043000	0.29575600
H	-6.20247900	3.40612200	1.52439700	H	-8.59961400	4.49324200	0.26599100
H	-4.98178500	-0.49177300	-0.40368900	H	-13.02126800	3.06533600	1.66234400
H	-2.28116700	-0.25050600	0.04935900	H	-12.47554100	0.44260500	1.20387200

TS

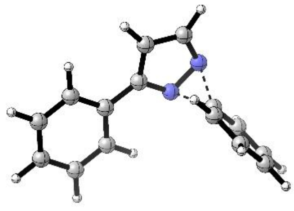

	298.15 K; 1 atm	773 K; 35 atm
Total Energy	-687.699456 Eh	-687.699456 Eh
Total Enthalpy	-687.455748 Eh	-687.389016 Eh
Total Gibbs free energy	-687.509185 Eh	-687.616571 Eh
Number of imaginary frequencies	1 (-600.80 cm^{-1})	1 (-600.80 cm^{-1})

C	0.82600000	2.67660000	0.06010000
C	-0.47460000	2.33970000	0.37730000
N	1.34950000	1.74510000	-0.76040000
N	0.38940000	0.75590000	-0.93450000
C	-0.70420000	1.09460000	-0.21510000
C	1.83800000	0.21370000	-0.25430000
C	2.69440000	-0.45610000	-1.12720000
C	1.84460000	-0.06690000	1.10610000
C	2.72090000	-1.02470000	1.59100000
C	3.58260000	-1.70080000	0.74010000
C	3.55850000	-1.40610000	-0.61970000
C	-1.85340000	0.20590000	-0.13230000
C	-3.01070000	0.58930000	0.54810000
C	-4.09110000	-0.27080000	0.63960000
C	-1.80600000	-1.06150000	-0.71780000
C	-2.88710000	-1.92000000	-0.62060000
C	-4.03380000	-1.52950000	0.05700000
H	1.41050000	3.53380000	0.35940000
H	-1.17140000	2.92920000	0.95340000
H	2.66850000	-0.21920000	-2.18320000
H	1.17090000	0.45800000	1.77290000
H	2.72280000	-1.24190000	2.65350000
H	4.26410000	-2.44790000	1.12790000
H	4.22510000	-1.92480000	-1.29990000
H	-3.06730000	1.56940000	1.00740000
H	-4.98340000	0.04270000	1.16960000
H	-0.91410000	-1.36540000	-1.25240000
H	-2.83450000	-2.90140000	-1.07830000
H	-4.88010000	-2.20280000	0.13030000

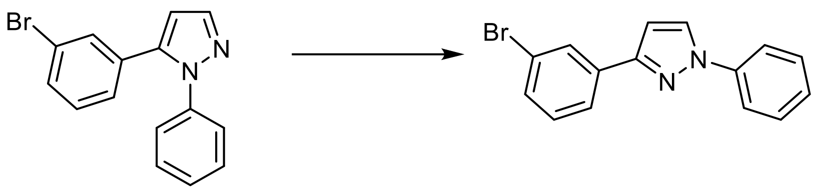

	ΔE (ΔE≠), kcal/mol	ΔH (ΔH≠), kcal/mol	ΔG (ΔG≠), kcal/mol	K = exp(-ΔG/RT)	k, s^{-1}	$t_{1/2}$, s
298.15 K; 1 atm	-3.7 (57.1)	-3.5 (54.7)	-3.7 (55.3)			
773 K; 35 atm (experiment conditions)	-3.7 (57.1)	-3.5 (54.5)	-3.9 (56.3)	12.7	0.0019	365

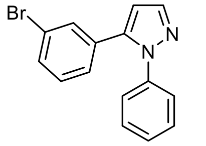			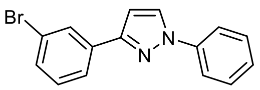		
	**298.15 K; 1 atm**	**773 K; 35 atm**		**298.15 K; 1 atm**	**773 K; 35 atm**
Total Energy	-3261.056302 Eh	-3261.056302 Eh	Total Energy	-3261.062172 Eh	-3261.062172 Eh
Total Enthalpy	-3260.817422 Eh	-3260.747698 Eh	Total Enthalpy	-3260.823056 Eh	-3260.753337 Eh
Total Gibbs free energy	-3260.876326 Eh	-3260.994186 Eh	Total Gibbs free energy	-3260.882185 Eh	-3261.00041 Eh
Number of imaginary frequencies	0	0	Number of imaginary frequencies	0	0

C	0.32929600	3.35044700	0.01677600	C	-0.73676000	1.94541000	-0.15959700
C	-0.91499000	2.70630800	0.02116500	C	0.63071100	1.98226300	-0.16486400
N	1.32380500	2.48145300	0.00186800	N	-1.09543900	0.64336500	-0.04161500
N	0.74126600	1.27263900	-0.00061300	N	-0.03217100	-0.16252300	0.02418900
C	-0.61970700	1.35828500	0.01961700	C	1.02934600	0.63088400	-0.04629600
C	1.55967600	0.12281700	-0.09762100	C	-2.39779900	0.10666600	-0.00871700
C	2.69845800	0.04153400	0.69179900	C	-3.47327400	0.90248000	0.36779900
C	1.23766100	-0.89631200	-0.98316900	C	-2.59632200	-1.22451400	-0.35464900
C	2.05066100	-2.01599100	-1.05610000	C	-3.87619800	-1.75159500	-0.32787500
C	3.18658600	-2.11066200	-0.26550500	C	-4.95852800	-0.96182700	0.03327200
C	3.51142900	-1.07610500	0.60084200	C	-4.74988000	0.36475800	0.37965600
C	-1.53511100	0.22054400	0.11633800	C	2.38084100	0.07407000	0.00721100
C	-2.69119400	0.21688100	-0.66304300	C	3.49093400	0.90915400	-0.11120900
C	-3.58549300	-0.83303500	-0.55215400	C	4.76269400	0.36822000	-0.05941000
C	-1.30519700	-0.83461400	0.99915300	C	2.58061900	-1.29662400	0.17707100
C	-2.21077100	-1.87671600	1.09075100	C	3.86096200	-1.81655000	0.22557600
C	-3.36110900	-1.88894000	0.31504000	C	4.97029500	-0.99008200	0.10771000
H	0.54318900	4.40919900	0.02328100	H	-1.46669800	2.73189500	-0.25542700
H	-1.89800300	3.14627400	0.06994000	H	1.24945200	2.86038600	-0.24966700
H	2.93619500	0.85717800	1.36283900	H	-3.31722700	1.92998100	0.67169800
H	0.36316300	-0.80950600	-1.61521400	H	-1.74559400	-1.82783600	-0.64137000
H	1.79920000	-2.81258100	-1.74661000	H	-4.02756700	-2.79008900	-0.59917100
H	3.82244900	-2.98593300	-0.33003500	H	-5.95845800	-1.37895400	0.04924800
H	4.40164200	-1.14002700	1.21584100	H	-5.58539200	0.98852000	0.67569800
H	-2.87538200	1.02584500	-1.35852600	H	3.36452600	1.97547400	-0.24535500
Br	-5.14722400	-0.82737400	-1.62148000	Br	6.25754400	1.52047800	-0.22259000
H	-0.41973600	-0.83310500	1.62228100	H	1.71905600	-1.94518900	0.27252900
H	-2.02605100	-2.69130000	1.78133300	H	4.00579300	-2.88263500	0.35843500
H	-4.07032700	-2.70352900	0.38698100	H	5.97315200	-1.39521400	0.14594700

TS

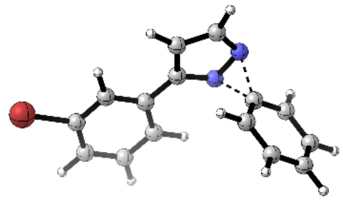

	298.15 K; 1 atm	773 K; 35 atm
Total Energy	-3260.965315 Eh	-3260.965315 Eh
Total Enthalpy	-3260.730213 Eh	-3260.660918 Eh
Total Gibbs free energy	-3260.788196 Eh	-3260.904419 Eh
Number of imaginary frequencies	1 (-600.88 cm^{-1})	1 (-600.88 cm^{-1})

C	0.83150000	2.66970000	-0.02980000
C	-0.46390000	2.33340000	0.30910000
N	1.35190000	1.72050000	-0.83180000
N	0.39770000	0.72190000	-0.97180000
C	-0.69070000	1.07360000	-0.25090000
C	1.85650000	0.20180000	-0.29140000
C	2.70760000	-0.48320000	-1.15680000
C	1.87710000	-0.04280000	1.07500000
C	2.76460000	-0.98260000	1.57520000
C	3.62210000	-1.67390000	0.73250000
C	3.58300000	-1.41430000	-0.63410000
C	-1.83330000	0.18000000	-0.13840000
C	-2.99440000	0.59510000	0.51590000
C	-4.06100000	-0.27560000	0.63080000
C	-1.77480000	-1.11210000	-0.66420000
C	-2.85350000	-1.96800000	-0.53340000
C	-4.01040000	-1.56080000	0.11550000
H	1.41390000	3.53740000	0.24190000
H	-1.15790000	2.93290000	0.87850000
H	2.66940000	-0.27280000	-2.21800000
H	1.20720000	0.49550000	1.73500000
H	2.77890000	-1.17280000	2.64270000
H	4.31280000	-2.40610000	1.13230000
H	4.24680000	-1.94560000	-1.30730000
H	-3.06500000	1.59310000	0.92800000
Br	-5.62980000	0.30720000	1.51630000
H	-0.87840000	-1.43700000	-1.17750000
H	-2.79850000	-2.97040000	-0.94190000
H	-4.85550000	-2.22940000	0.21710000

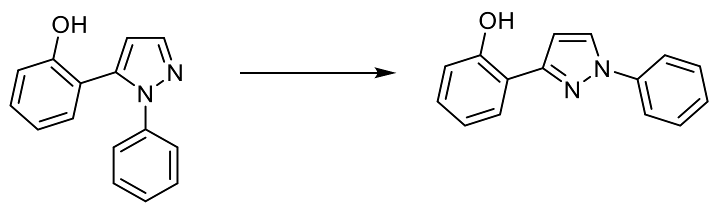

	ΔE (ΔE≠), kcal/mol	ΔH (ΔH≠), kcal/mol	ΔG (ΔG≠), kcal/mol	K = exp(-ΔG/RT)	k, s^{-1}	$t_{1/2}$, s
298.15 K; 1 atm	-3.3 (56.3)	-3.3 (54.0)	-3.9 (54.5)			
773 K; 35 atm (experiment conditions)	-3.3 (56.3)	-3.2 (53.7)	-4.9 (55.4)	24.1	0.0035	198

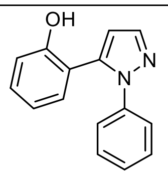			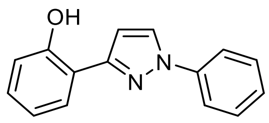		
	**298.15 K; 1 atm**	**773 K; 35 atm**		**298.15 K; 1 atm**	**773 K; 35 atm**
Total Energy	-762.973237 Eh	-762.973237 Eh	Total Energy	-762.978524 Eh	-762.978524 Eh
Total Enthalpy	-762.720170 Eh	-762.649135 Eh	Total Enthalpy	-762.725384 Eh	-762.654292 Eh
Total Gibbs free energy	-762.776967 Eh	-762.89197 Eh	Total Gibbs free energy	-762.783159 Eh	-762.899763 Eh
Number of imaginary frequencies	0	0	Number of imaginary frequencies	0	0

C	0.32991700	3.36044300	0.07756700	C	-0.74220800	1.99227800	-0.05339100
C	-0.92397600	2.73704400	0.05061800	C	0.62423700	2.05738300	-0.06333900
N	1.31189500	2.47720000	0.05277900	N	-1.07492900	0.67945100	-0.00698300
N	0.70903200	1.27881800	0.01186000	N	0.00462800	-0.10692300	0.00990000
C	-0.65284300	1.38492700	0.02073100	C	1.05466700	0.70862500	-0.01933100
C	1.51069600	0.11996800	-0.10019900	C	-2.36403000	0.11414700	-0.00627600
C	2.65545200	0.01680000	0.67858100	C	-3.46134700	0.86986300	0.39101700
C	1.17002000	-0.88870200	-0.99126500	C	-2.53130000	-1.20714500	-0.40462500
C	1.96887900	-2.01706700	-1.08065100	C	-3.79919200	-1.76304700	-0.40964800
C	3.11039100	-2.13285500	-0.30083200	C	-4.90227000	-1.01261300	-0.02789800
C	3.45432200	-1.10956000	0.57131800	C	-4.72552200	0.30380000	0.37126500
C	-1.56286500	0.24141400	0.10973400	C	2.40384800	0.14078600	0.01980700
C	-2.69447300	0.15578100	-0.71052300	C	3.55938600	0.90112700	-0.20260700
C	-3.55396800	-0.93226500	-0.60686500	C	4.81545700	0.30545700	-0.15978900
C	-1.34109500	-0.77921200	1.03394400	C	2.56862100	-1.22136100	0.28484900
C	-2.19497300	-1.86239900	1.13988800	C	3.81495200	-1.81631900	0.32673400
C	-3.30498900	-1.93736000	0.31138500	C	4.94737700	-1.04568700	0.10251300
H	0.56002500	4.41550900	0.10865500	H	-1.48636600	2.76959400	-0.10277100
H	-1.90012200	3.19183700	0.07436500	H	1.23161800	2.94296100	-0.10281600
H	2.90868000	0.82380600	1.35432300	H	-3.33183300	1.88831100	0.73493300
H	0.29031600	-0.78612400	-1.61334900	H	-1.66475000	-1.77956000	-0.70671000
H	1.70147200	-2.80490200	-1.77532100	H	-3.92459400	-2.79338600	-0.72241100
H	3.73479600	-3.01539100	-0.37785500	H	-5.89271200	-1.45205700	-0.03693900
H	4.34860300	-1.18919500	1.17868700	H	-5.57705200	0.89733100	0.68365800
O	-2.91250900	1.14635700	-1.60497400	O	3.43298200	2.22474500	-0.46685800
H	-4.42299700	-0.98562700	-1.25647400	H	5.69537800	0.91738400	-0.33826500
H	-0.47655400	-0.71154100	1.68371400	H	1.67702500	-1.80971800	0.46330100
H	-1.99749000	-2.63972700	1.86776600	H	3.90364900	-2.87535800	0.53728200
H	-3.98519100	-2.77860400	0.38015800	H	5.93444400	-1.49263900	0.13304700
H	-3.70685100	0.94403000	-2.11026700	H	4.30913700	2.59807300	-0.60744900

TS

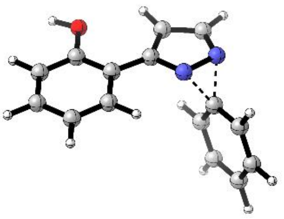

	298.15 K; 1 atm	773 K; 35 atm
Total Energy	-762.883464 Eh	-762.883463 Eh
Total Enthalpy	-762.634187 Eh	-762.563575 Eh
Total Gibbs free energy	-762.690131 Eh	-762.803699 Eh
Number of imaginary frequencies	1 (-597.29 cm^{-1})	1 (-597.29 cm^{-1})

C	-1.14110000	1.33660000	-0.51610000
C	0.20540000	1.63890000	-0.47040000
N	-1.31530000	0.00810000	-0.38380000
N	-0.06260000	-0.55930000	-0.19210000
C	0.86200000	0.43120000	-0.20330000
C	-1.15560000	-0.81720000	1.06960000
C	-0.97720000	-0.08420000	2.23680000
C	-1.73880000	-2.08420000	1.10580000
C	-2.14270000	-2.60260000	2.32010000
C	-1.97500000	-1.88310000	3.49940000
C	-1.39070000	-0.62640000	3.44300000
C	2.25500000	0.11330000	0.06970000
C	3.27550000	1.07510000	0.04420000
C	4.58600000	0.71950000	0.33910000
C	2.60830000	-1.19990000	0.39850000
C	3.91030000	-1.55420000	0.69310000
C	4.90530000	-0.58690000	0.66250000
H	-1.99050000	1.99010000	-0.64880000
H	0.66200000	2.60130000	-0.62430000
H	-0.51990000	0.89730000	2.19700000
H	-1.86570000	-2.63620000	0.18320000
H	-2.59740000	-3.58700000	2.34440000
H	-2.29530000	-2.29870000	4.44690000
H	-1.24870000	-0.05070000	4.35120000
O	2.95630000	2.35250000	-0.27200000
H	5.35890000	1.48250000	0.31320000
H	1.82660000	-1.94950000	0.41350000
H	4.14880000	-2.58040000	0.94430000
H	5.93300000	-0.84660000	0.88910000
H	3.75500000	2.88990000	-0.24730000

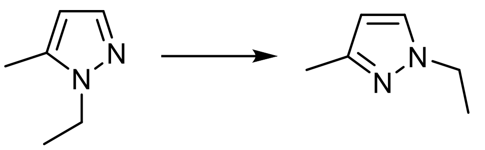

	ΔE (ΔE≠), kcal/mol	ΔH (ΔH≠), kcal/mol	ΔG (ΔG≠), kcal/mol	K = exp(-ΔG/RT)	k, s^{-1}	$t_{1/2}$, s
298.15 K; 1 atm	0.0 (68.7)	-0.2 (66.0)	-0.6 (65.9)			
773 K; 35 atm (experiment conditions)	0.0 (68.7)	-0.1 (66.0)	-1.4 (65.7)	2.4	$4.3\cdot10^{-6}$	161722 (45 h)

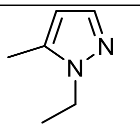			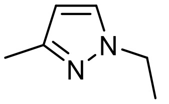		
	**298.15 K; 1 atm**	**773 K; 35 atm**		**298.15 K; 1 atm**	**773 K; 35 atm**
Total Energy	-343.889992 Eh	-343.889992	Total Energy	-343.890048 Eh	-343.890048 Eh
Total Enthalpy	-343.725023 Eh	-343.686622	Total Enthalpy	-343.725284 Eh	-343.68678 Eh
Total Gibbs free energy	-343.767002 Eh	-343.843031	Total Gibbs free energy	-343.767983 Eh	-343.84523 Eh
Number of imaginary frequencies	0	0	Number of imaginary frequencies	0	0

C	-0.23718000	-1.71165800	-0.76843400	C	0.57835700	-1.71251200	-0.64014700
C	0.38197500	-2.44252800	0.40614700	C	1.08264700	-2.16759600	0.71370900
H	-1.16602000	-2.19109400	-1.08191100	H	0.15079500	-2.55161400	-1.19360500
H	0.44161200	-1.71090800	-1.62266200	H	1.38837100	-1.29428900	-1.24022800
H	1.30705700	-1.95155700	0.71384100	H	1.53056000	-1.33660500	1.26361100
H	0.61429100	-3.47345000	0.12887000	H	1.83865000	-2.94663500	0.59034400
H	-0.29673200	-2.46327600	1.26202700	H	0.26363100	-2.57385800	1.30936400
C	-1.45940000	1.53370500	0.19159500	C	-1.53287000	1.19943000	-0.48860500
C	-0.10382000	1.70982800	-0.12036500	C	-0.32031300	0.62310900	-0.78957000
C	-1.70473600	0.19453100	-0.03885800	C	-2.33705300	0.13079000	-0.04675600
N	0.45181400	0.57480600	-0.51306900	N	-0.43868000	-0.69187800	-0.53339100
N	-0.53246200	-0.33076600	-0.46324200	N	-1.65477500	-1.00837800	-0.07797500
C	-2.94784400	-0.60245800	0.10629100	C	-3.75364200	0.15673500	0.40301200
H	-2.17042100	2.27036300	0.53054600	H	-1.80321900	2.23937700	-0.58153900
H	0.48838800	2.61270400	-0.08234200	H	0.60098400	1.04288600	-1.16328100
H	-3.26757500	-1.03410900	-0.84670000	H	-4.41703300	0.51163300	-0.39048900
H	-3.75296500	0.03688400	0.46803500	H	-3.88638400	0.82186100	1.26057300
H	-2.82768300	-1.42381700	0.81833200	H	-4.07172700	-0.84535500	0.69297400

TS

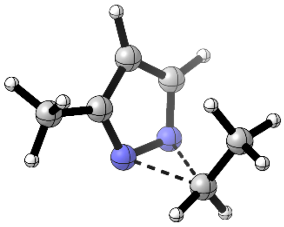

	298.15 K; 1 atm	773 K; 35 atm
Total Energy	-343.780541 Eh	-343.780541 Eh
Total Enthalpy	-343.619778 Eh	-343.581516 Eh
Total Gibbs free energy	-343.661979 Eh	-343.738371 Eh
Number of imaginary frequencies	1 (-642.43 cm^{-1})	1 (-642.43 cm^{-1})

C	0.11680000	-1.73780000	-0.96240000
C	0.58740000	-1.87790000	0.43230000
H	-0.69970000	-2.40260000	-1.21710000
H	0.86480000	-1.70700000	-1.74310000
H	1.53750000	-1.37680000	0.61250000
H	0.69410000	-2.94790000	0.64590000
H	-0.15570000	-1.48920000	1.13690000
C	-1.20240000	0.94660000	0.60840000
C	0.02910000	0.97620000	-0.02850000
C	-1.99110000	0.08430000	-0.15810000
N	-0.00790000	0.22390000	-1.13250000
N	-1.28720000	-0.33120000	-1.21540000
C	-3.39410000	-0.36010000	0.05720000
H	-1.51080000	1.51880000	1.47210000
H	0.92820000	1.51190000	0.24270000
H	-3.75980000	-0.88530000	-0.82630000
H	-4.05610000	0.48780000	0.24960000
H	-3.47510000	-1.03670000	0.91360000

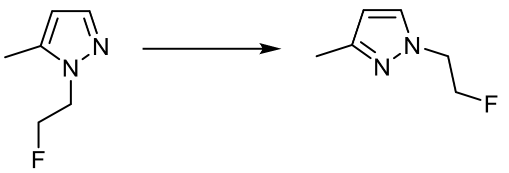

	ΔE (ΔE≠), kcal/mol	ΔH (ΔH≠), kcal/mol	ΔG (ΔG≠), kcal/mol	K = exp(-ΔG/RT)	k, s^{-1}	$t_{1/2}$, s
298.15 K; 1 atm	0.0 (69.4)	-0.1 (67.1)	-0.7 (67.5)			
773 K; 35 atm (experiment conditions)	0.0 (69.4)	-0.1 (66.9)	-1.6 (68.3)	2.9	$7.9 \cdot 10^{-7}$	878722 (244 h)

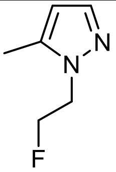			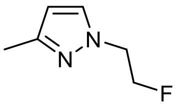		
	**298.15 K; 1 atm**	**773 K; 35 atm**		**298.15 K; 1 atm**	**773 K; 35 atm**
Total Energy	-443.076491 Eh	-443.076491 Eh	Total Energy	-443.076479 Eh	-443.076479 Eh
Total Enthalpy	-442.917707 Eh	-442.877895 Eh	Total Enthalpy	-442.917940 Eh	-442.878006 Eh
Total Gibbs free energy	-442.962068 Eh	-443.042654 Eh	Total Gibbs free energy	-442.963173 Eh	-443.04523 Eh
Number of imaginary frequencies	0	0	Number of imaginary frequencies	0	0

C	-0.25194200	-1.70351400	-0.76033500	C	0.56622500	-1.70344900	-0.64752100
C	0.41094200	-2.38713400	0.41745000	C	1.04241100	-2.13232800	0.72471400
H	-1.18055100	-2.21512300	-1.01593300	H	0.15457700	-2.56119100	-1.18326000
H	0.40983000	-1.73068100	-1.62719100	H	1.39961200	-1.29932800	-1.22363200
H	1.35787600	-1.90153700	0.66364800	H	1.47707100	-1.29045100	1.27077600
F	0.67003100	-3.70610000	0.08120000	F	2.02242400	-3.10051200	0.57198700
H	-0.23993800	-2.38268100	1.29638000	H	0.22303400	-2.56112600	1.30516900
C	-1.46732000	1.53863500	0.19312400	C	-1.53782400	1.20402200	-0.49107200
C	-0.10590500	1.70762400	-0.10039100	C	-0.32603000	0.63127400	-0.79410200
C	-1.71915700	0.20256000	-0.03978800	C	-2.34187200	0.13458100	-0.04743600
N	0.44985700	0.57156600	-0.48571500	N	-0.44404300	-0.68574500	-0.53870200
N	-0.54222900	-0.32947400	-0.44909000	N	-1.66111700	-1.00414700	-0.07977900
C	-2.96871800	-0.58695000	0.08526200	C	-3.75699500	0.16243100	0.40546100
H	-2.17810800	2.27985700	0.52203100	H	-1.81000600	2.24332400	-0.58464800
H	0.49014200	2.60740900	-0.05286300	H	0.59359100	1.05124000	-1.17149000
H	-3.27716300	-1.01305600	-0.87378200	H	-4.41994800	0.52134100	-0.38639900
H	-3.77436700	0.05685200	0.43735300	H	-3.88548300	0.82650000	1.26434200
H	-2.86498100	-1.41105100	0.79674200	H	-4.07732700	-0.83933100	0.69359100

TS

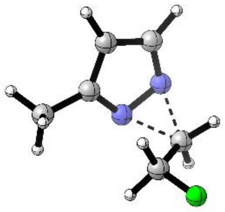

	298.15 K; 1 atm	773 K; 35 atm
Total Energy	-442.965876 Eh	-442,965876
Total Enthalpy	-442.810828 Eh	-442,771359
Total Gibbs free energy	-442.854497 Eh	-442,933854
Number of imaginary frequencies	1 (-654.29 cm^{-1})	1 (-654.29 cm^{-1})

C	0.34570000	-1.54500000	-0.87280000
C	-0.20950000	-2.36330000	0.26000000
H	0.27960000	-2.01320000	-1.84570000
H	1.36060000	-1.23110000	-0.63670000
H	-0.35980000	-1.75770000	1.15590000
F	0.74150000	-3.33400000	0.56620000
H	-1.13550000	-2.86890000	-0.01680000
C	-1.07830000	1.31000000	0.27950000
C	0.02410000	1.28830000	-0.56520000
C	-1.79980000	0.15720000	-0.01540000
N	-0.05020000	0.23530000	-1.38160000
N	-1.19600000	-0.48100000	-1.03200000
C	-3.04880000	-0.36810000	0.59650000
H	-1.35400000	2.08960000	0.97510000
H	0.83860000	1.99390000	-0.64840000
H	-3.46900000	-1.16180000	-0.02280000
H	-3.79870000	0.42010000	0.69470000
H	-2.87230000	-0.77320000	1.59740000

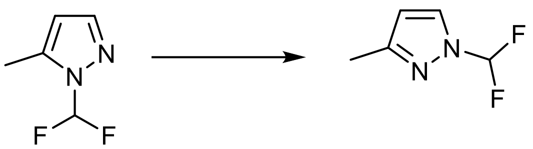

	ΔE (ΔE≠), kcal/mol	ΔH (ΔH≠), kcal/mol	ΔG (ΔG≠), kcal/mol	K = exp(-ΔG/RT)	k, s^{-1}	$t_{1/2}$, s
298.15 K; 1 atm	-2.1 (68.4)	-2.2 (66.2)	-2.9 (66.0)			
773 K; 35 atm (experiment conditions)	-2.1 (68.4)	-2.1 (65.9)	-3.9 (65.9)	13.1	$3.7\cdot10^{-6}$	184210 (51 h)

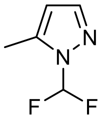			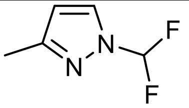		
	**298.15 K; 1 atm**	**773 K; 35 atm**		**298.15 K; 1 atm**	**773 K; 35 atm**
Total Energy	-502.994285 Eh	-502.994285 Eh	Total Energy	-502.997583 Eh	-502.997583 Eh
Total Enthalpy	-502.872728 Eh	-502.837333 Eh	Total Enthalpy	-502.876304 Eh	-502.840758 Eh
Total Gibbs free energy	-502.915245 Eh	-502.991042 Eh	Total Gibbs free energy	-502.919833 Eh	-502.997335 Eh
Number of imaginary frequencies	0	0	Number of imaginary frequencies	0	0

C	-0.24338800	-1.70334100	-0.69017500	C	0.52315700	-1.72774900	-0.61528100
F	0.66106900	-2.21916900	0.16838700	F	1.56146600	-1.54197800	0.23217400
H	-1.15905300	-2.29401400	-0.64503400	H	0.05110400	-2.69172700	-0.42772600
F	0.29223500	-1.80544400	-1.92405400	F	1.05158900	-1.70028600	-1.86076600
C	-1.47145500	1.52080000	0.17847800	C	-1.48835900	1.19650500	-0.34322100
C	-0.09021200	1.68075900	-0.05247600	C	-0.26866900	0.63405900	-0.59374000
C	-1.72247500	0.18969500	-0.03782400	C	-2.35117000	0.10839000	-0.06027800
N	0.48427600	0.54648100	-0.38648600	N	-0.43555500	-0.70362700	-0.45854300
N	-0.51620000	-0.35286500	-0.37551700	N	-1.69907400	-1.03675900	-0.13419600
C	-2.98350300	-0.58649200	0.04817400	C	-3.79549600	0.14403700	0.28227400
H	-2.19102200	2.27104900	0.46432700	H	-1.73499200	2.24622500	-0.35891400
H	0.50308900	2.58135300	0.01288300	H	0.68727500	1.06188800	-0.85189800
H	-3.25667400	-1.03004100	-0.91321200	H	-4.37658300	0.61471400	-0.51490200
H	-3.79350300	0.07704100	0.34911400	H	-3.96821500	0.71861400	1.19599300
H	-2.91950000	-1.39032900	0.78677900	H	-4.17102000	-0.86810900	0.43370800

TS

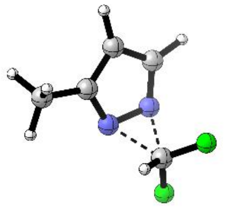

	298.15 K; 1 atm	773 K; 35 atm
Total Energy	-502.885308 Eh	-502.885308 Eh
Total Enthalpy	-502.767273 Eh	-502.732361 Eh
Total Gibbs free energy	-502.810013 Eh	-502.886041 Eh
Number of imaginary frequencies	1 (-663.17 cm^{-1})	1 (-663.17 cm^{-1})

C	0.28170000	-1.52470000	-0.41470000
F	1.28410000	-1.24980000	0.43430000
H	-0.40810000	-2.17330000	0.12090000
F	0.75380000	-2.07920000	-1.50250000
C	-1.42480000	1.30420000	0.23760000
C	-0.10620000	1.29080000	-0.20060000
C	-2.00110000	0.14200000	-0.26800000
N	0.09230000	0.24240000	-0.99850000
N	-1.09630000	-0.47980000	-1.04080000
C	-3.36960000	-0.40630000	-0.08390000
H	-1.91490000	2.07560000	0.81360000
H	0.69210000	1.99140000	-0.00220000
H	-4.12920000	0.30720000	-0.41300000
H	-3.56950000	-0.63410000	0.96660000
H	-3.49060000	-1.32110000	-0.66540000

Synthetic method

We strongly recommend following corresponding safety procedures. When heated, capillaries may explode with a loud bang.

Capillary preparation method (HTCS):

Pasteur pipettes made of chemical glass (Borosilikatglas 5.1 230 mm Duran) were used in the manufacture of capillaries. The capillary was sealed from the thin part with a burner flame (pencil torch). The samples were then filled with a solution of the substance and sealed. In the synthesis of several tens of milligrams, several tens of capillaries were produced. Capillaries were placed in a vertical state in an aluminum thermoblock. Then, the samples were placed in a heating element. For induction heating, a graphite glass inside the induction heater was used. Otherwise, the samples were placed in a muffle furnace.

One should pay attention to the quality of the sealant, since this greatly affects the course of the experiment. Capillaries in which carbon soot had already formed due to the evaporation of solvent vapor and subsequent contact with the hot part of the glass were prone to rupture when heated to 500 °C. This is explained by the large defectiveness of glass at the sealing site due to the emergence of a new phase, such as amorphous carbon, during carbonization of the solvent at the junction, which significantly reduces the quality of this capillary. Another limiting factor in the use of chemical glass capillaries is the maximum synthesis temperature. Capillaries made of chemical glass begin to melt at approximately 700 °C. To overcome this limitation, quartz glass can be used, which increases the synthesis temperature to 1100–1200 °C. Quartz glass has fewer imperfections and a coefficient of thermal expansion, which allows syntheses to be carried out with greater internal pressure inside the capillary than when chemical glass is used.

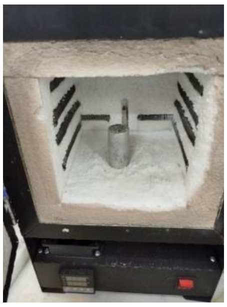

Figure S1. Muffle furnace “satellite” Russia. Maximum heating temperature: 1200 °C.

Measurement of pressure inside the reaction medium:

In a vertical steel reactor with a volume of 60 mL, 15 mL of toluene was added. The reaction medium was purged with argon. The initial pressure was 1 atm. The reactor was then closed with an output to the monometer. The reactor was heated to 500 °C; after the set temperature was established after 10 minutes of heating, the pressure was measured. The pressure at the time of heating increased monotonically, and the highest recorded value was 32.3 bar. The reactor then cooled. After the experiment, the volume of the solution did not change.

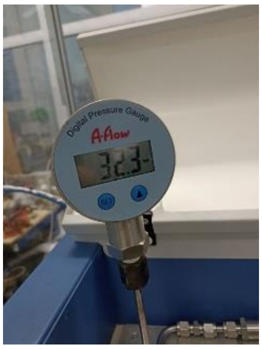

Figure S2. Photo of a steel reactor pressure gauge heated to 500 °C.

Induction furnace for syntheses:

The induction heating board module (China, 1000 W, voltage 12-48 V) and power unit (China, CHUX S-1000-24, DC input 20-28 V 40 A) were used. Changing the voltage at the output of the power supply allows the temperature to be adjusted. The capillary was placed vertically inside an aluminum block, which was located in a craft glass. The graphite glass is located inside the inductor. Temperature measurements were carried out on the outer part of the aluminum block with a pyrometer ("KELVIN KB DIPOLE PYROCELSUS" Russia).

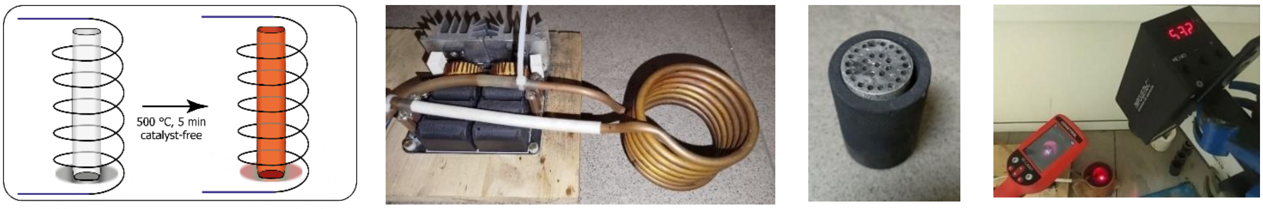

A B C D

Figure S3. A - Schematic representation of induction heating in capillaries, B - photo of the inductor, C - photo of a graphite glass with an aluminum block for capillaries, and D - photo of an experiment with induction heating.

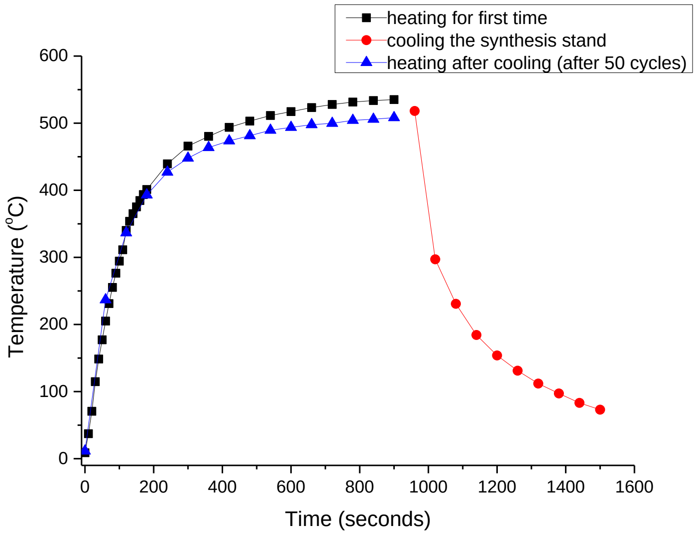

Figure S4. Induction heating operating mode.

Influence of inert gas on the reaction:

The capillary, which is sealed on one side, is filled with the prepared mixture of 1,5-diphenylpyrazole in *p*-xylene. After that, the capillary is purged with argon via a gas ball with an outlet in the form of a syringe for 10–15 seconds. After that, the capillary was sealed. The capillary was then placed in a heating cassette and placed in a muffle furnace heated to the desired temperature.

There are no differences in the course of the reaction depending on the gas atmosphere that fills the capillary. There was no difference in the GC–MS data between the samples filled with argon and those filled with air. This is most likely due to the small amount of gas that is present in the capillary, which does not have a significant effect on the course of the reaction.

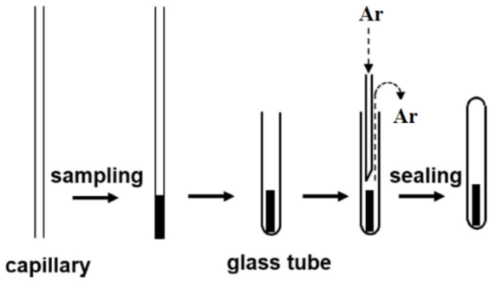

Figure S5. The capillary was sealed with blowing argon.

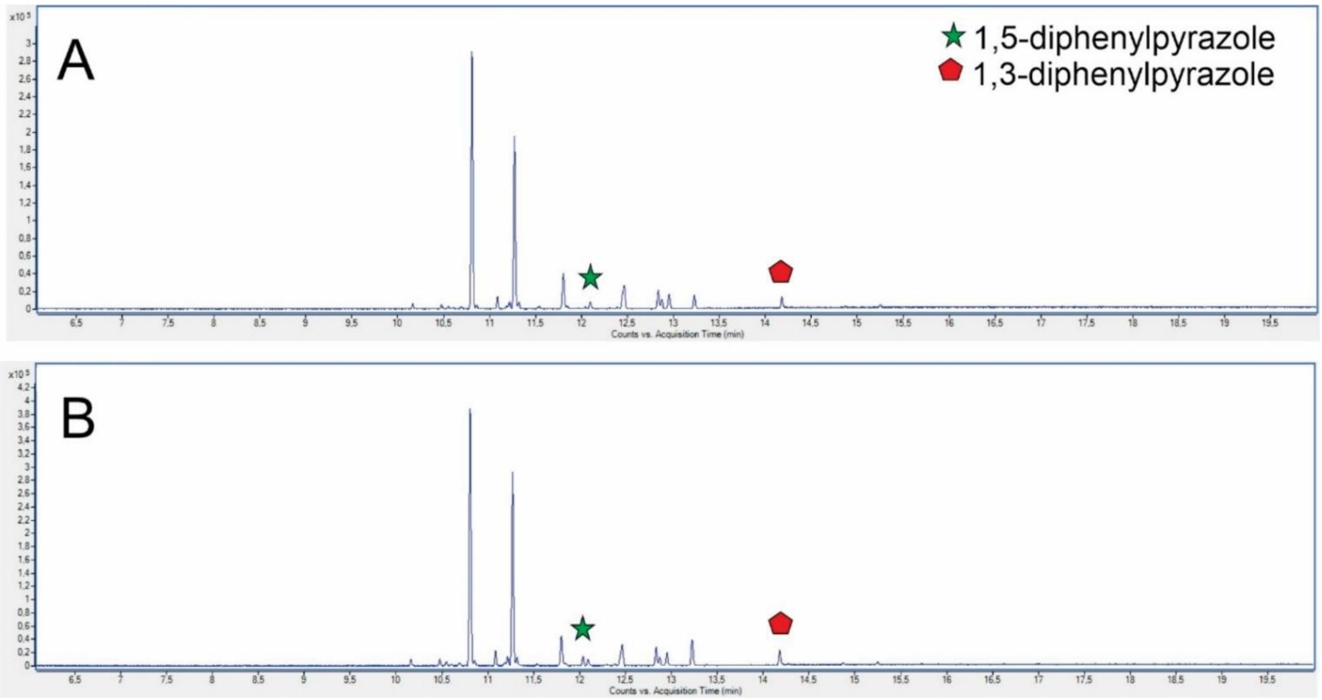

Figure S6. A - capillary filled with argon, B - capillary filled with air. GC–MS data of the reaction mixture after synthesis at 500 °C for 5 minutes.

Various solvents for high-temperature organic synthesis:

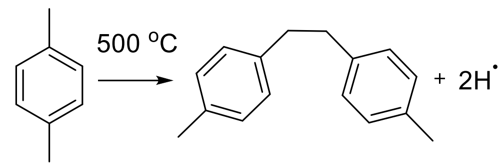

Scheme S1. Formation of expected byproduct.

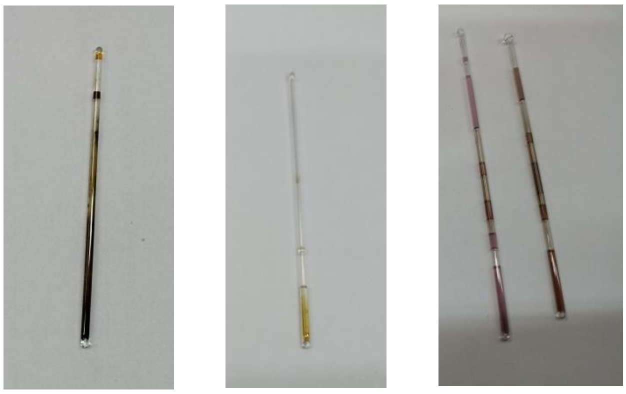

A B C

Figure S7. A - photo of the capillary after the reaction without solvent, B - photo of a capillary after the reaction in p-xylene, C - photo of a capillary with 1,2-dichloromethane after heating.

Analysis of the stability of the isomerization reaction product and the starting 1,5-diphenylpyrazole:

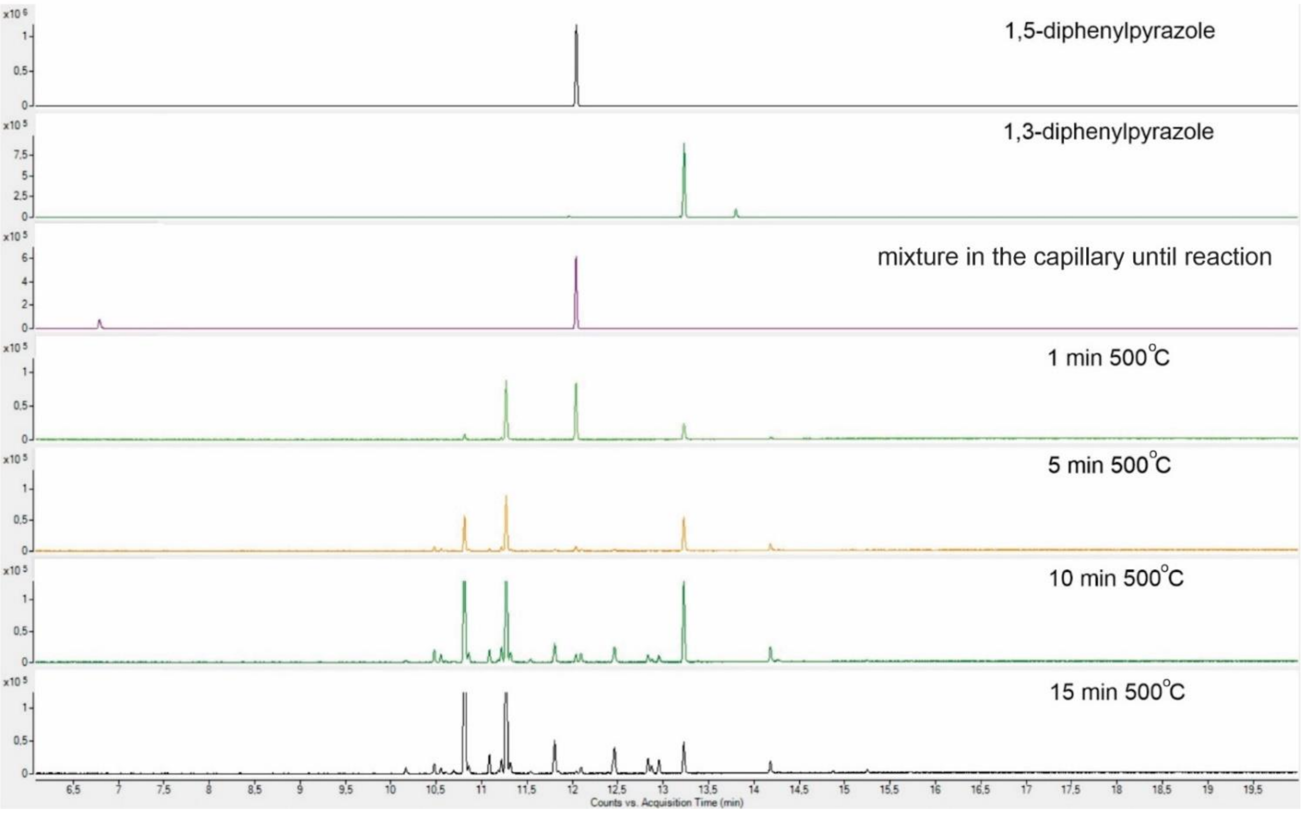

Figure S8. GC data for the isomerization of 1,5-diphenylpyrazole.

TGA analysis:

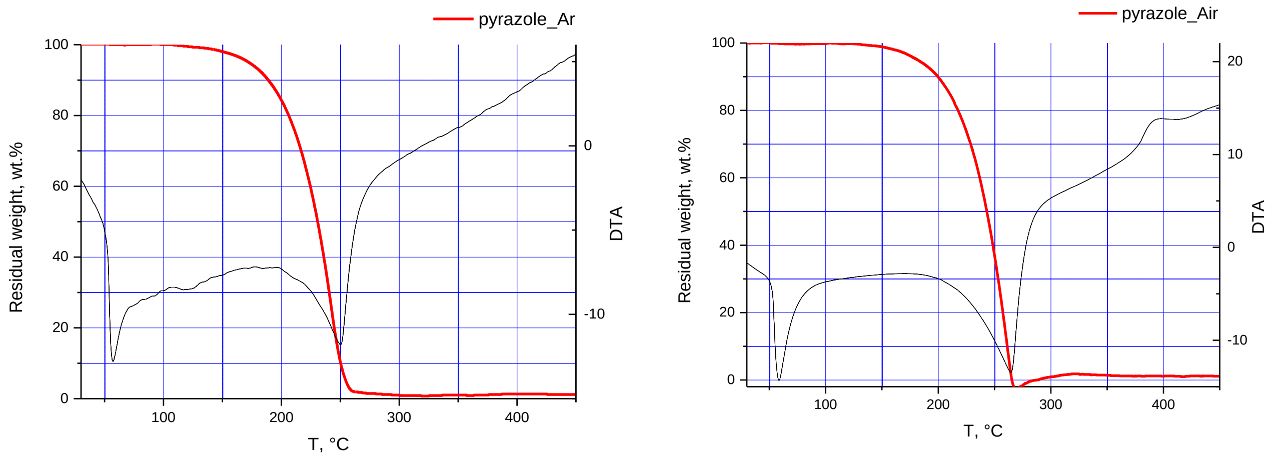

A B

Figure S9. TGA results for heating 1,5-diphenylpyrazole in argon (A) and air (B).

Catalyst search

The capillary was filled with a solution of 25 µL of *p*-xylene in which 3 mg of 1,5-diphenylpyrazole was dissolved. A total of 0.5 mg of the substance being tested for catalytic activity was added to the capillary. The capillary was subsequently placed in an oven at 500 °C for 3 minutes. A reference capillary was placed in the oven together with a capillary with a substance being tested for catalytic activity without introducing the substance. After synthesis, the capillary was opened on both sides, and the contents were transferred into a vial, where 600 µL of acetone-d_6 was added. After stirring, the mixture was transferred to an NMR tube.

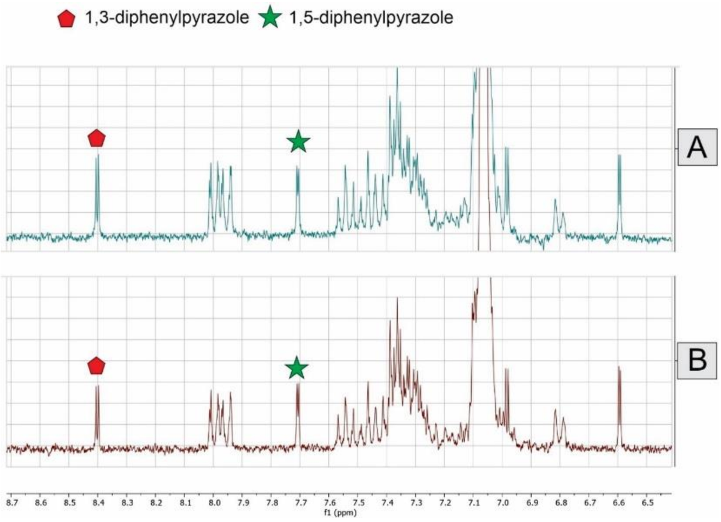

Figure S10. An example of a method for comparing and searching for substances with catalytic activity. A – ^{1}H NMR experiment without additives. B – ^{1}H NMR experiment with the addition of CuI.

Tested substances for catalytic activity. Alkali, alkaline earth metals (bases): NaOH, CsCl, $MgSO_4$, Zn(II) triflate, $Ba(OH)_2$, $Sr(OH)_2$.

Tested substances for catalytic activity. 3, 13 Group of elements: Sc(III) triflate, Dy(III) triflate, Bi(III) triflate, Yb(III) triflate, Ce(III) triflate, $EuCl_3$, In(III) triflate, Al(III) triflate.

Tested substances for catalytic activity. 11, 12 Group of elements: $Cu(OAc)_2$, CuSCN, CuI, $CuSO_4$, CuO, Cu, Cu_2O, CuBr, AgCN, HgI_2, Au salts.

Tested substances for catalytic activity. 6, 7, 14 Group of elements: NH_4ReO_4, $CrCl_3$, MnO_2, MoS_2, $Pb(C_2H_5O_2)_2$, $SnCl_2$.

Tested substances for catalytic activity. 8, 9, 10 Group of elements: Pd/C (10%), Pd(II) acetate, Pd(II) chloride, $RuCl_3$, $Ni(ClO_4)_2$, $CoBr_2$, Fe(II) acetate.

Tested substances for catalytic activity: $Et_2O•BF_3$, HCl, DBU.

All of the above compounds did not possess desired catalytic activity.

Kinetics measurement

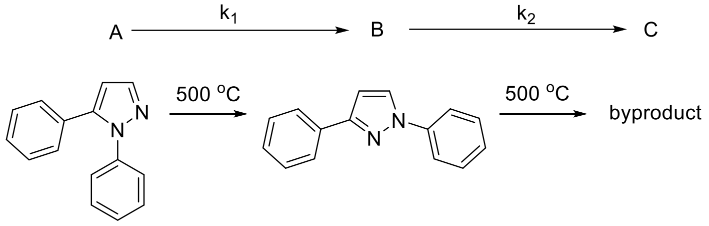

Formula S1. Kinetics of a sequential reaction for the isomerization of pyrazole.

$$\begin{cases} \frac{d[\mathrm{A}]}{dt} = -k_1[\mathrm{A}] \\ \frac{d[\mathrm{A}]}{dt} = k_1[\mathrm{A}] - k_2[\mathrm{B}] \\ \frac{d[\mathrm{C}]}{dt} = k_2[\mathrm{C}] \end{cases}$$

$[B]_o=[C]_o=0$

$$\begin{cases} [\mathrm{A}] = \mathrm{A}_0 \exp(-k_1 t) \\ [\mathrm{B}] = \mathrm{A}_0 \frac{k_1}{k_2 - k_1} [\exp(-k_1 t) - \exp(-k_2 t)] \end{cases}$$

$$t_{max} = \frac{\ln\left(\frac{k_2}{k_1}\right)}{k_2 - k_1}$$

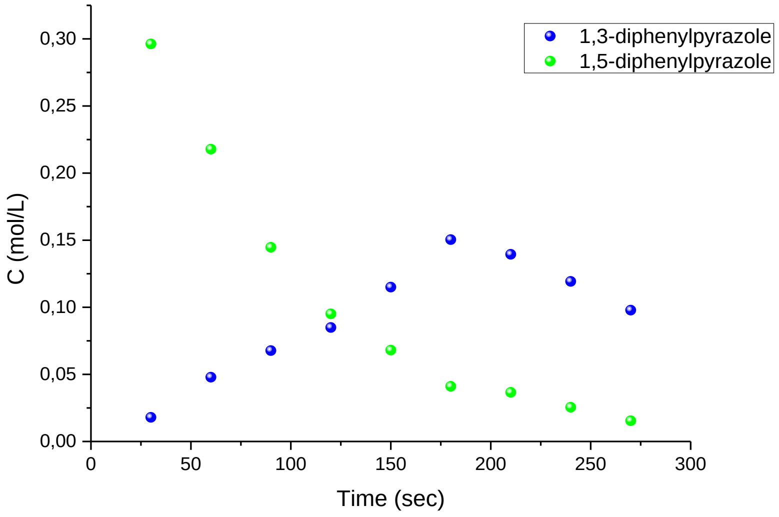

Figure S11. Kinetic curves of changes in the concentration of 1,3-diphenylpyrazole and 1,5-diphenylpyrazole at 500 °C.

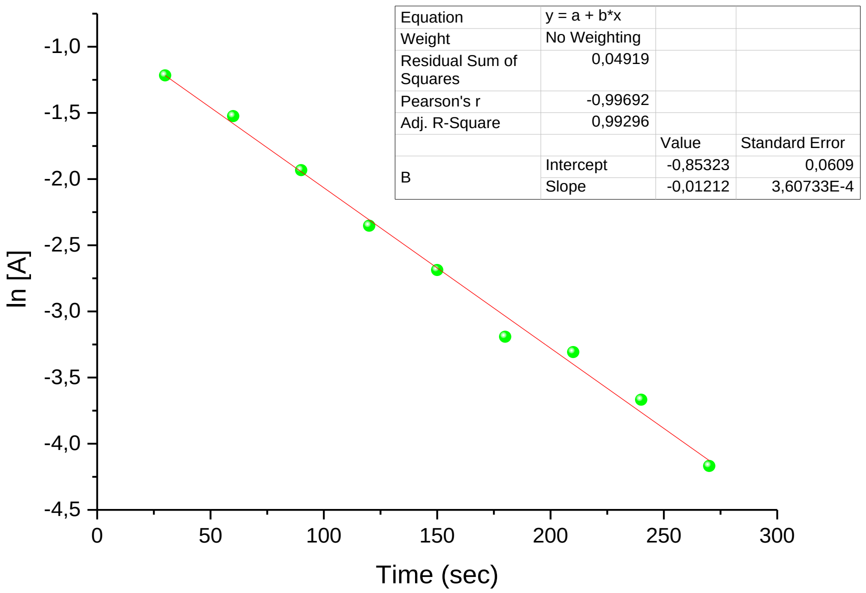

Figure S12. Logarithmic dependence of the change in the concentration of 1,3-diphenylpyrazole, the product of the isomerization reaction at 500 °C

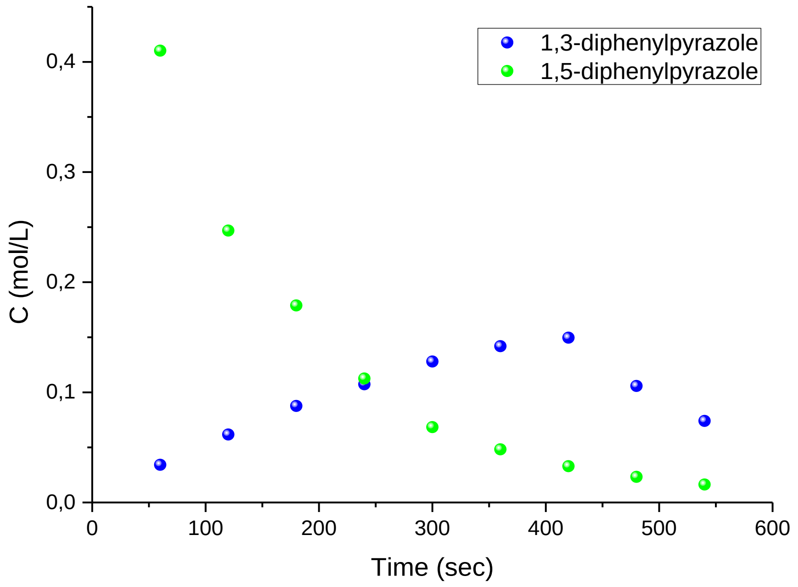

Figure S13. Kinetic curves of changes in the concentration of 1,3-diphenylpyrazole and 1,5-diphenylpyrazole at 480 °C.

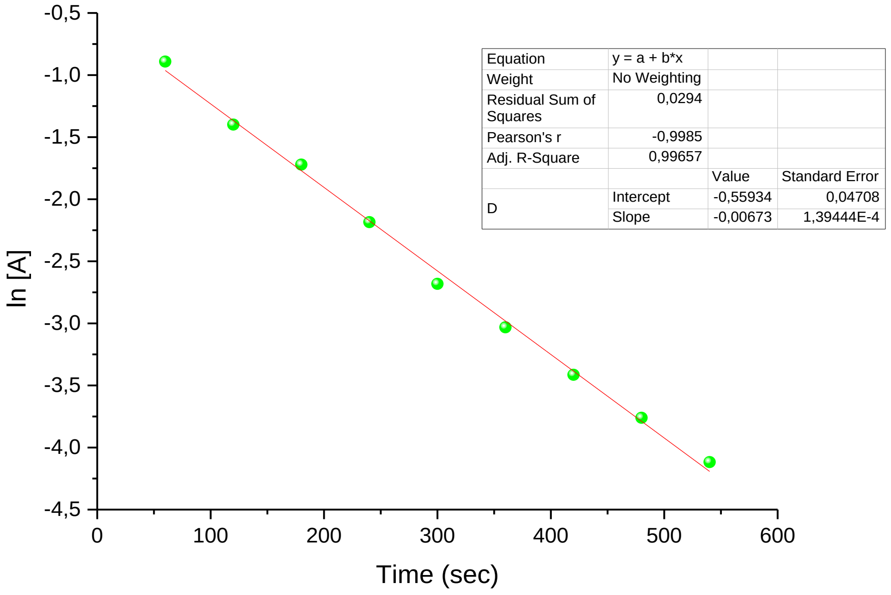

Figure S14. Logarithmic dependence of changes in the concentration of 1,3-diphenylpyrazole, the product of the isomerization reaction at 480 °C.

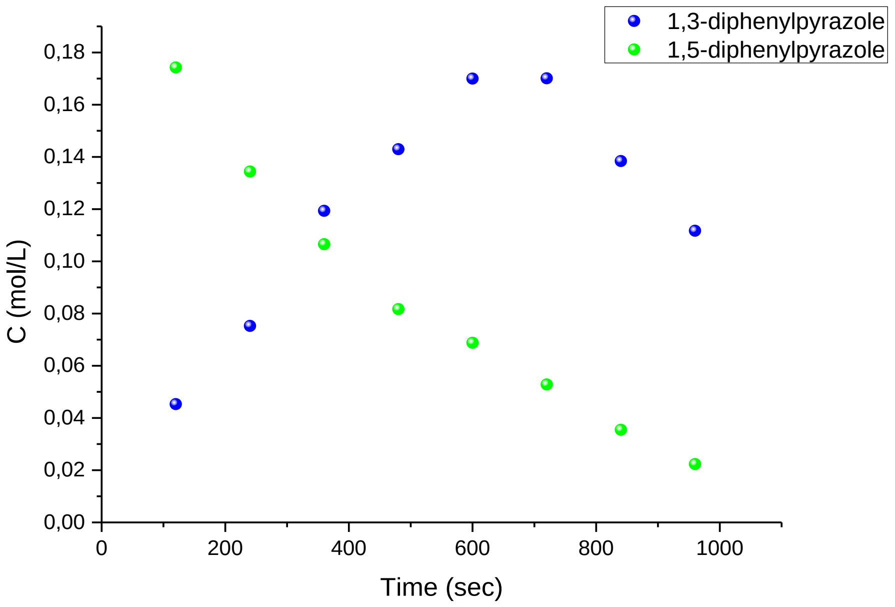

Figure S15. Kinetic curves of changes in the concentration of 1,3-diphenylpyrazole and 1,5-diphenylpyrazole at 460 °C.

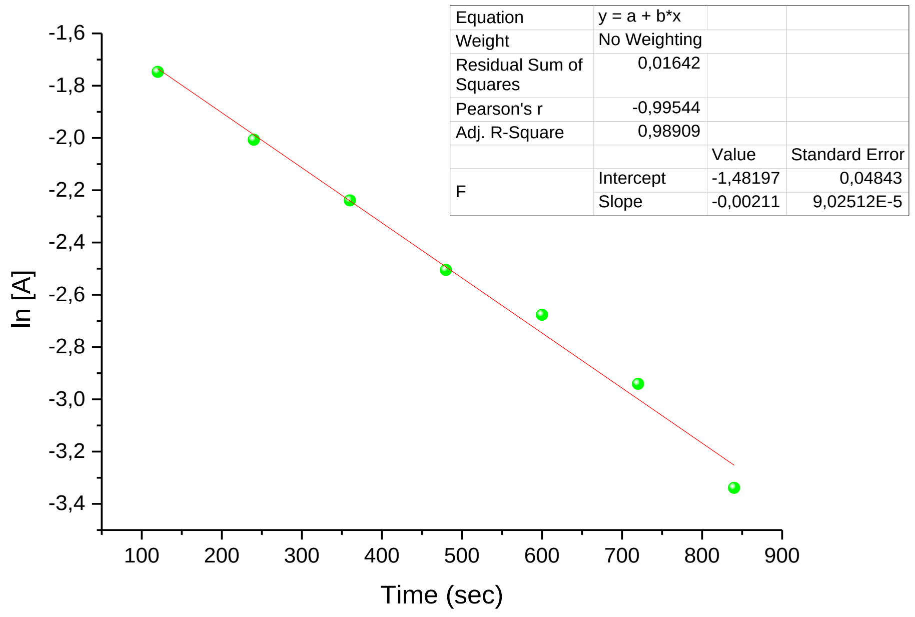

Figure S16. Logarithmic dependence of changes in the concentration of 1,3-diphenylpyrazole, the product of the isomerization reaction at 460 °C.

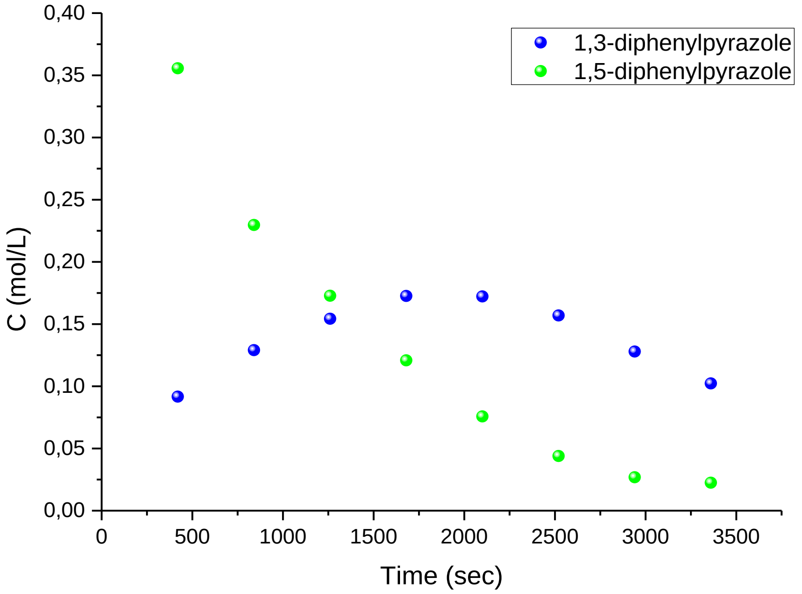

Figure S17. Kinetic curves of changes in the concentration of 1,3-diphenylpyrazole and 1,5-diphenylpyrazole at 440 °C.

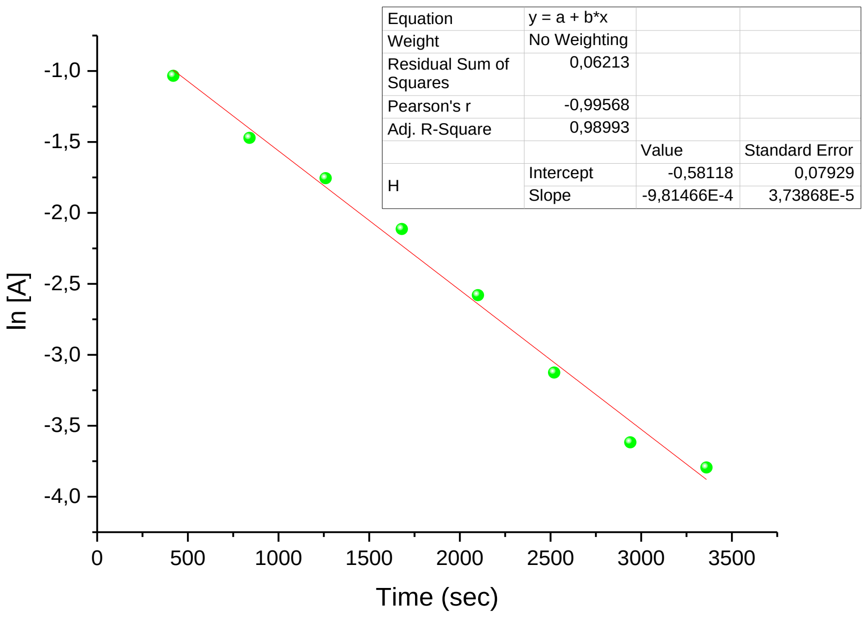

Figure S18. Logarithmic dependence of changes in the concentration of 1,3-diphenylpyrazole, the product of the isomerization reaction at 440 °C.

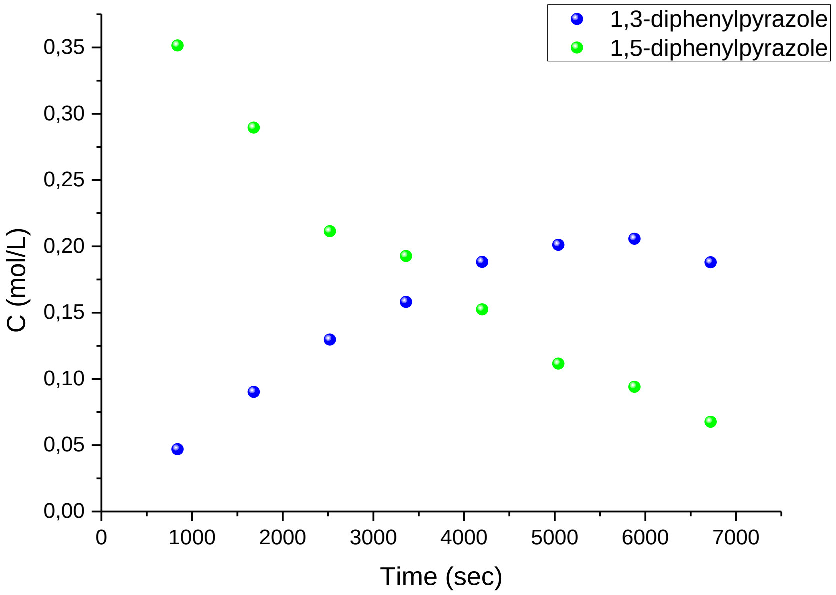

Figure S19. Kinetic curves of changes in the concentration of 1,3-diphenylpyrazole and 1,5-diphenylpyrazole at 420 °C.

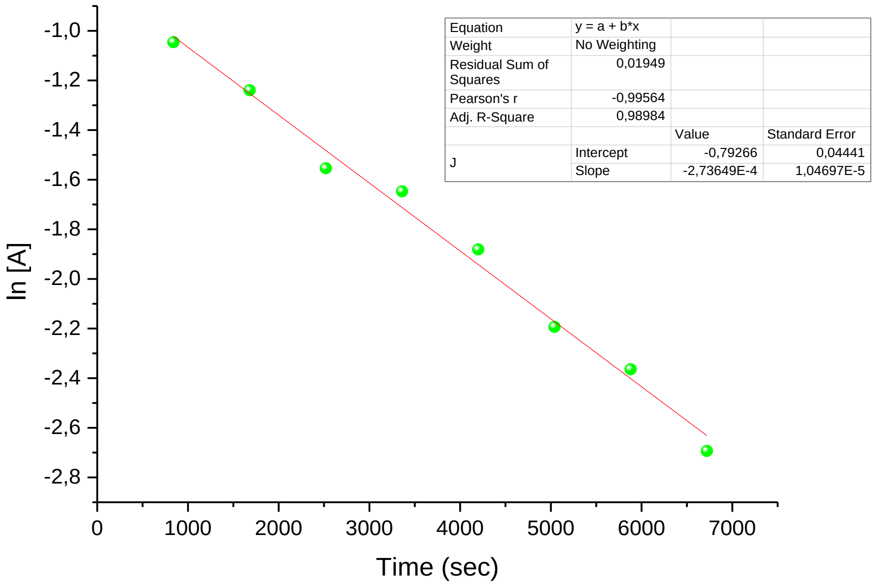

Figure S20. Logarithmic dependence of changes in the concentration of 1,3-diphenylpyrazole, the product of the isomerization reaction at 420 °C.

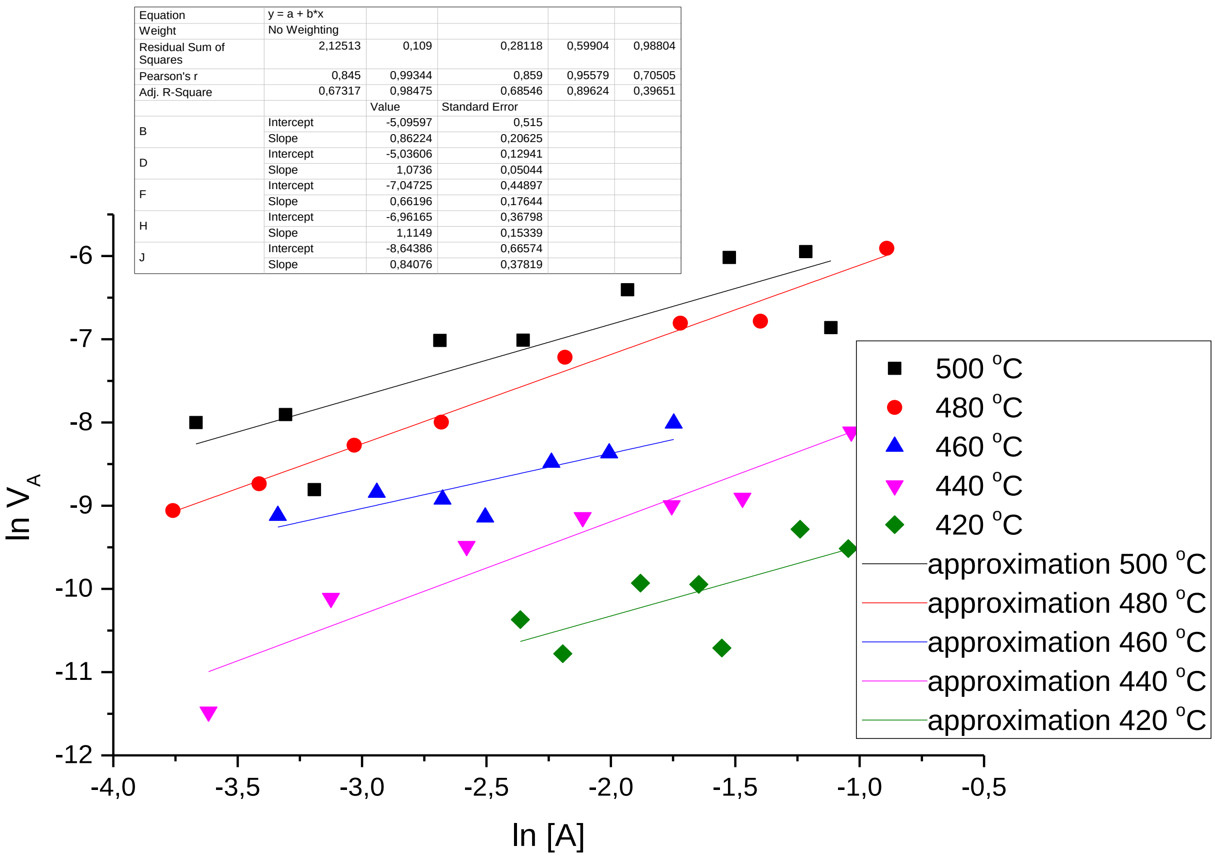

Equation	y = a + b*x				
Weight	No Weighting				
Residual Sum of Squares	2,12513	0,109	0,28118	0,59904	0,98804
Pearson's r	0,845	0,99344	0,859	0,95579	0,70505
Adj. R-Square	0,67317	0,98475	0,68546	0,89624	0,39651
		Value	Standard Error		
B	Intercept	-5,09597	0,515		
	Slope	0,86224	0,20625		
D	Intercept	-5,03606	0,12941		
	Slope	1,0736	0,05044		
F	Intercept	-7,04725	0,44897		
	Slope	0,66196	0,17644		
H	Intercept	-6,96165	0,36798		
	Slope	1,1149	0,15339		
J	Intercept	-8,64386	0,66574		
	Slope	0,84076	0,37819		

Figure S21. Calculation of the reaction order for 1,3-diphenylpyrazole.

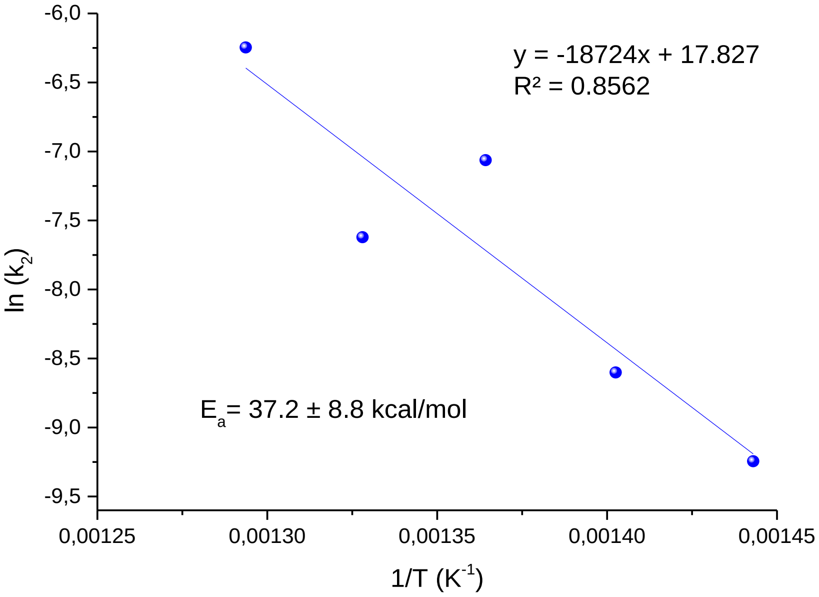

Figure S22. The activation energy of the degradation of 1,3-diphenylpyrazole was calculated on the basis of the change in the concentration of the isomerization reaction product.

Mechanistic study

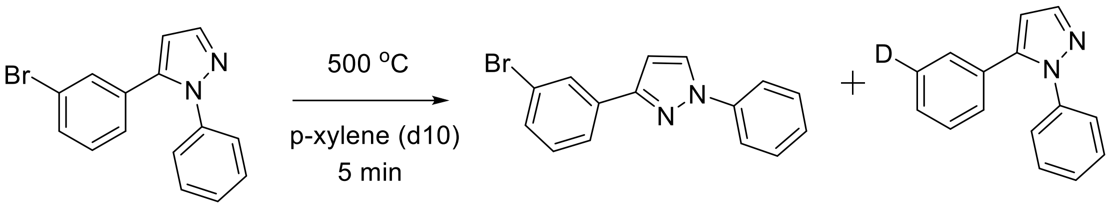

Scheme S2. The behavior of the reaction mixture in a deuterated solvent was studied.

The reaction was carried out in *p*-xylene-*d10*. After 5 min of reaction, the reaction mixture was analyzed via ESI-HRMS. MS detected the formation of deuterated compounds, but nondeuterated 1,5-diphenylpyrazole was not detected.

Experimental peak $[M]^+$ = 221.1061 Da, calculated for $C_{15}H_{11}N_2D$ = 221.1058, Δ = 1.36 ppm.
Experimental peak $[M]^+$ = 222.1113 Da, calculated for $C_{15}H_{10}N_2D_2$ = 222.1121, Δ = 3.60 ppm.
Experimental peak $[M]^+$ = 223.1178 Da, calculated for $C_{15}H_9N_2D_3$ = 223.1183, Δ = 1.79 ppm.
Experimental peak $[M]^+$ = 224.1239 Da, calculated for $C_{15}H_8N_2D_4$ = 224.1246, Δ = 3.12 ppm.
Experimental peak $[M]^+$ = 225.1303 Da, calculated for $C_{15}H_7N_2D_5$ = 225.1309, Δ = 2.67 ppm.

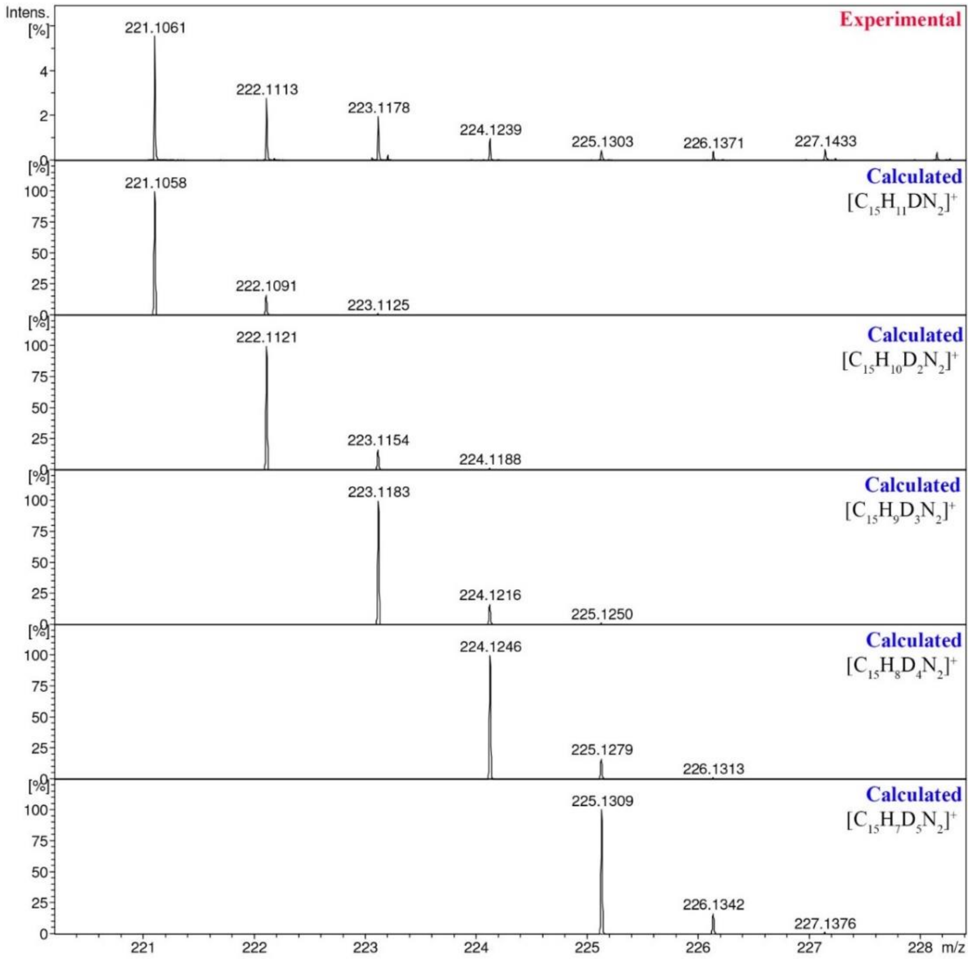

Figure S23. Experimental and theoretical ESI-(+) HRMS spectra of 1,5-diphenylpyrazole in a p-xylene d10 solution.

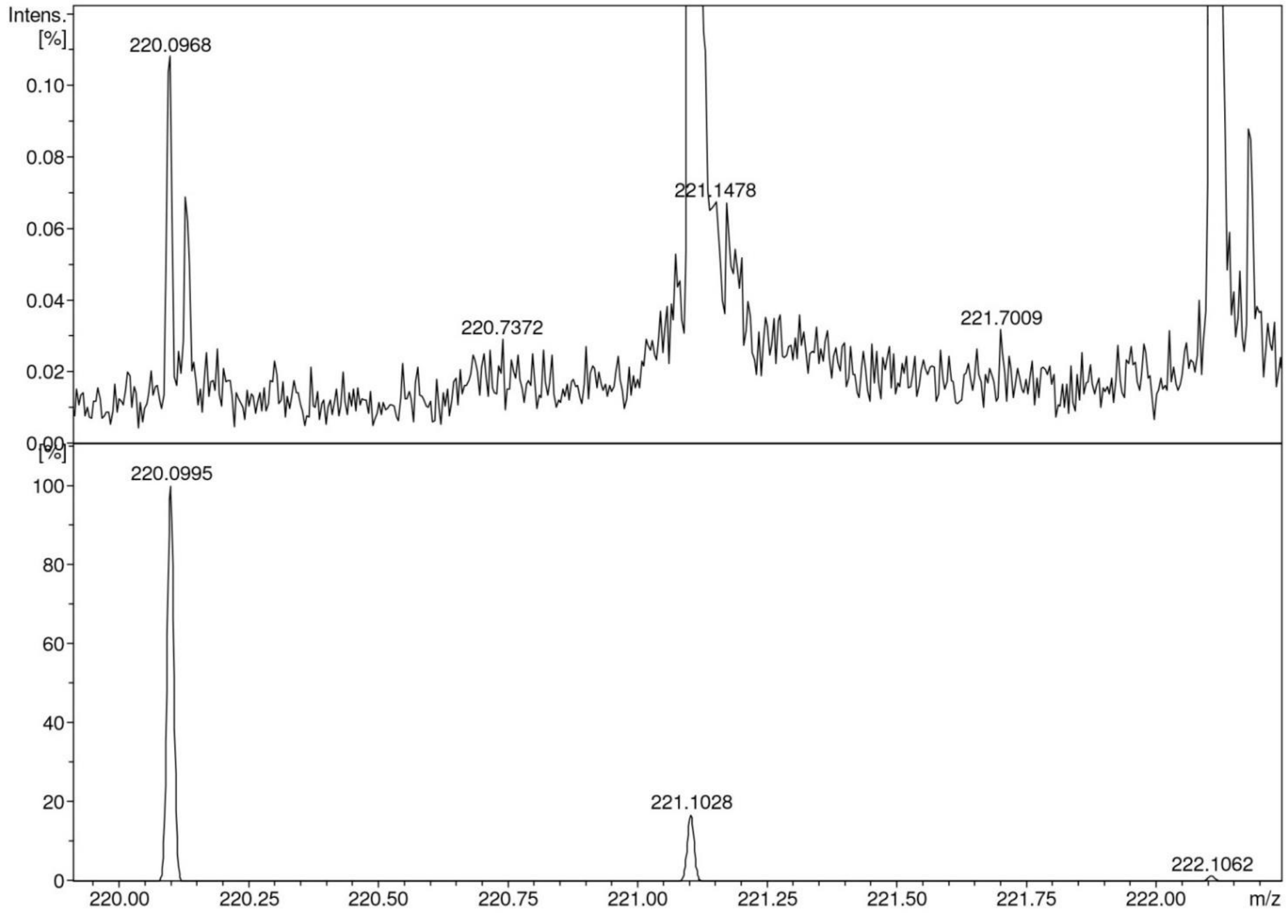

Figure S24. ESI-HRMS The absence of deuterated 1,5-diphenyl pyrazole is shown. Experimental peak $[M]^+$ = 220.0968 Da, calculated for $C_{15}H_{11}N_2D$ = 220.0995, Δ = 12.27 ppm.

Preparation of pyrazoles and isomerization reactions

Synthesis of enaminones. General procedure:

DMFDA (6.5 mmol) was added to a solution of acetophenone (5 mmol) in dry toluene (4 mL) under argon. The reaction mixture was heated to 110 °C until TLC was complete (2 days). After cooling, the solvent was evaporated under reduced pressure, and the resulting residue was purified via crystallization.[15]

Synthesis of pyrazoles (1a, 2a, 3a). General procedure:

Hydrazine hydrochloride (1 mmol) was added to a mixture of enaminoketone (0.9 mmol) and Na_2CO_3 (0.6 mmol) in MeOH (10 mL) and H_2O (2 mL) under stirring at room temperature. The resulting mixture was acidified with glacial acetic acid to pH 4 and heated to 135 °C for 2 hours. After cooling, the suspension was diluted with EtOAc (30 mL), the organic layer was washed with H_2O (3 × 10 mL), and the solvent was evaporated under reduced pressure. The resulting residue was purified via chromatography on a silica gel with EtOAc/hexane as the eluent.[16,17]

Synthesis of isomerization pyrazoles (1b, 2b, 3b, 4b, 5b, 6b). General procedure:

Isomerization was carried out according to a previously described procedure (HTCS). The capillary with the substance in the p-xylene solution was sealed on both sides. The reaction was carried out at 500 °C for 5 minutes. After cooling, the capillaries broke. The suspension was diluted with EtOAc (30 mL), the organic layer was washed with H_2O (3 × 10 mL), and the solvent was evaporated under reduced pressure. The resulting residue was purified via chromatography on a silica gel with EtOAc/hexane as the eluent.

Product characterization:

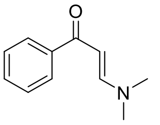

*precursor**1a**: (2E)-3-(Dimethylamino)-1-phenyl-2-propen-1-one*

Yield 647 mg (74%); yellow solid; chromatography after crystallization with Et_2O. (EtOAc/Hexanes = 1:10). **^1H NMR (300 MHz, $CDCl_3$, ppm)** δ 7.90 - 7.86 (m, 2H) 7.83 - 7.79 (d, 1H, J = 12.3 Hz), 7.44 - 7.37 (m, 3H), 5.72 - 5.68 (d, 1H, J = 12.4 Hz), 3.09 (s, 3H), 2.92 (s, 3H). **^{13}C {^1H} NMR (75 MHz, $CDCl_3$, ppm)** δ 188.6, 154.4, 140.5, 131.0, 128.2 (2C), 127.6 (2C), 92.3. **ESI-(+) HRMS:** *m/z* calcd for $C_{11}H_{13}NO$ $[M+H]^+$, 176.1070; found 176.1062 (Δ = 4.54 ppm).

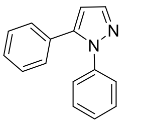

***1a**: 1,5-Diphenyl-1H–pyrazole*

Yield 160 mg (81%); yellow solid; chromatography (EtOAc/hexanes = 1:10). **^1H NMR (300 MHz, $CDCl_3$, ppm)** δ 7.75 - 7.75 (d, 1H, J = 1.4 Hz), 7.36 - 7.23 (m, 10H), 6.54 - 6.53 (d, 1H, J = 1.4 Hz). **^{13}C {^1H} NMR (75 MHz, $CDCl_3$, ppm)** δ 143.2, 140.4, 140.2, 130.7, 129.0 (2C), 128.9 (2C), 128.6 (2C), 128.4, 127.6, 125.4 (2C), 108.0. **ESI-(+) HRMS:** *m/z* calcd for $C_{15}H_{12}N_2$ $[M+H]^+$, 221.1073; found 221.1076 (Δ = 1.35 ppm).

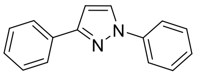

***1b**: 1,3-Diphenyl-1H-pyrazole*

Yield 31.1 mg (50%); yellow solid; chromatography (EtOAc/hexanes = 1:10). **^1H NMR (300 MHz, $CDCl_3$, ppm)** δ 7.97 - 7.96 (d, 1H, J = 2.5 Hz), 7.94 - 7.90 (m, 2H), 7.80 - 7.76 (m, 2H), 7.51 - 7.40 (m, 4H), 7.37 - 7.26 (m, 2H), 6.79 - 6.78 (d, 1H, J = 2.5 Hz). **^{13}C {^1H} NMR (75 MHz, $CDCl_3$, ppm)** δ 153.1, 140.4, 133.3, 129.6, 128.8 (2C), 128.2 (2C), 128.1, 126.5, 126.0 (2C), 119.2 (2C), 105.2. **ESI-(+) HRMS:** *m/z* calcd for $C_{15}H_{12}N_2$ $[M+H]^+$, 221.1073; found 221.1065 (Δ = 3.62 ppm).

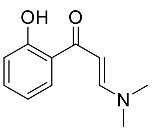

*Precursor**2a**: (2E)-3-(Dimethylamino)-1-(2-hydroxyphenyl)-2-propen-1-one*

Yield 620 mg (65%); white solid; after crystallization, Et_2O. **^1H NMR (300 MHz, $CDCl_3$, ppm)** δ 7.91 (s, 1H), 7.71 - 7.67 (dd, 1H, J = 8.0 Hz, J = 1.6 Hz), 7.38 - 7.32 (td, 1H, J = 7.8 Hz, J = 1.6 Hz), 6.95 - 6.92 (dd, 1H, J = 8.4 Hz, J = 8.4 Hz, J = 0.8 Hz), 6.86 - 6.81 (td, 1H, J = 7.5 Hz, J = 1.1 Hz), 5.81 (s, 1H), 3.10 (s, 6H). **^{13}C {^1H} NMR (75 MHz, $CDCl_3$, ppm)** δ 191.7, 163.1, 154.9, 134.1, 128.4, 120.5, 118.4, 118.1, 90.3, 45.5, 37.6. **ESI-(+) HRMS:** *m/z* calcd for $C_{11}H_{13}NO_2$ $[M+H]^+$, 192.1019; found 192.1024 (Δ = 2.60 ppm).

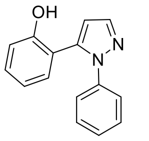

***2a**: 2-(1-Phenyl-1*H*-pyrazol-5-yl)phenol*

Yield 155 mg (73%); chromatography (EtOAc/hexanes = 1:10). **^1H NMR (300 MHz, DMSO-d_6, ppm)** δ 9.56 (s, 1H), 7.72 - 7.71 (d, 1H, J = 1.7 Hz), 7.71 - 7.17 (m, 6H), 7.11 - 7.08 (dd, 1H, J = 7.7 Hz, J = 1.6 Hz), 6.82 - 6.77 (m, 2H), 6.47 - 6.46 (d, 1H, J = 1.8 Hz). **^{13}C {^1H} NMR (75 MHz, DMSO-d_6, ppm)** δ 154.9, 140.7, 139.7, 139.7, 131.0, 130.2, 128.6 (2C), 126.7, 123.2 (2C), 118.9, 118.8, 115.7, 108.8. **ESI-(+) HRMS:** *m/z* calcd for $C_{15}H_{12}N_2O$ $[M+H]^+$, 237.1022; found 237.1024 (Δ = 0.84 ppm).

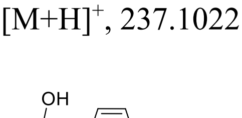

***2b**: 3-(1-Phenyl-1*H*-pyrazol-2-yl)phenol*

Yield 5.6 mg (34%); white solid; chromatography (EtOAc/hexanes = 1:10). **^1H NMR (300 MHz, $CDCl_3$, ppm)** δ 10.80 (s, 1H), 7.99 - 7.98 (d, 1H, J = 2.7 Hz), 7.71 - 7.67 (m, 2H), 7.64 - 7.61 (dd, 1H, J = 7.7 Hz, J = 1.6 Hz), 7.53 - 7.47 (t, 2H, J = 8.3 Hz), 7.36 - 7.23 (m, 2H), 7.10 - 7.05 (d, 1H, J = 8.3 Hz), 6.97 - 6.92 (t, 1H, J = 7.4 Hz), 6.87 - 6.86 (d, 1H, J = 2.7 Hz). **^{13}C {^1H} NMR (75 MHz, $CDCl_3$, ppm)** δ 156.2, 153.1, 139.4, 129.8, 129.8 (2C), 127.9, 127.0, 126.7, 119.5, 119.0 (2C), 117.3, 116.4, 104.70. **ESI-(+) HRMS:** *m/z* calcd for $C_{15}H_{12}N_2O$ $[M+H]^+$, 237.1022; found 237.1023 (Δ = 0.42 ppm).

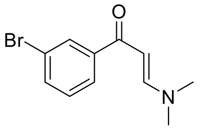

*Precursor**3a**: (2E)-1-(3-Bromophenyl)-3-(dimethylamino)-2-propen-1-one*

Yield 873 mg (69%); brown solid; after crystallization, Et_2O. **^{1}H NMR (300 MHz, $CDCl_3$, ppm)** δ 8.01 (t, 1H, J = 1.7 Hz), 7.87 - 7.79 (m, 2H), 7.58 - 7.55 (ddd, 1H, J = 8.1 Hz, J = 0.9 Hz, J = 0.8 Hz), 7.30 - 7.25 (t, 1H, J = 7.8 Hz), 5.62 (d, 2H, J = 12.3 Hz), 3.16 (s, 3H), 2.95 (s, 3H). **^{13}C {^{1}H} NMR (75 MHz, $CDCl_3$, ppm)** δ 186.9, 154.8, 142.7, 133.8, 130.7, 129.9, 126.2, 122.6, 91.9, 45.2, 37.5. **ESI-(+) HRMS:** *m/z* calcd for $C_{11}H_{12}NOBr$ $[M+H]^+$, 254.0175; found 254.0180 (Δ = 1.97 ppm).

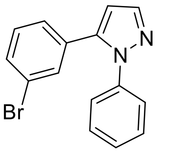

***3a**: 5-(3-Bromophenyl)-1-phenyl-1H-pyrazole*

Yield 193 mg (72%); yellow oil; chromatography (EtOAc/hexanes = 1:10). **^{1}H NMR (300 MHz, DMSO-*d*$_6$, ppm)** δ 7.79 - 7.78 (d, 1H, J = 1.8 Hz), 7.56 - 7.53 (ddd, 1H, J = 8.0 Hz, J = 3.0 Hz, J = 1.0 Hz) 7.48 - 7.37 (m, 4H), 7.34 - 7.27 (3H), 7.19 - 7.16 (m, 1H), 6.76 - 6.75 (d, 1H, J = 1.8 Hz). **^{13}C {^{1}H} NMR (75 MHz, DMSO-*d*$_6$, ppm)** δ 140.8, 140.3, 139.5, 132.3, 131.0, 130.8, 130.6, 129.1 (2C), 127.9, 127.4, 125.3 (2C), 121.7, 108.4. **ESI-(+) HRMS:** *m/z* calcd for $C_{15}H_{11}N_2Br$ $[M+H]^+$, 299.0178; found 299.0175 (Δ = 1.00 ppm).

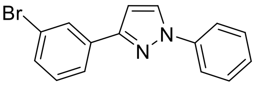

***3b**:3-(3-Bromophenyl)-1-phenyl-1H-pyrazole*

Yield 6.5 mg (31%); brown oil; chromatography (EtOAc/hexanes = 1:10). **^{1}H NMR (300 MHz, DMSO-*d*$_6$, ppm)** δ 8.61 - 8.60 (d, 1H, J = 2.6 Hz), 8.14 - 8.13 (t, 1H, J = 1.6 Hz), 7.97, - 7.92 (m, 3H), 7.58 - 7.51 (m, 3H), 7.46 - 7.41 (t, 1H, J = 7.9 Hz), 7.37 - 7.32 (t, 1H, J = 7.3 Hz), 7.15 - 7.14 (d, 1H, J = 2.6 Hz).**^{13}C {^{1}H} NMR (75 MHz, DMSO-*d*$_6$, ppm)** δ 150.3, 139.5, 135.5, 131.0, 130.7 (2C), 129.7, 129.6, 127.8, 126.4, 124.4, 122.2, 118.4 (2C), 105.8. **ESI-(+) HRMS:** *m/z* calcd for $C_{15}H_{11}N_2Br$ $[M+H]^+$, 299.0178; found 299.0187 (Δ = 3.00 ppm).

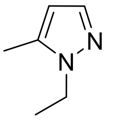

***4a**:1-Ethyl-5-methyl-1H-pyrazole*

^{1}H NMR (300 MHz, $CDCl_3$) δ 7.44 (d, 1H, J = 1.7 Hz), 6.05 (m, 1H), 4.20 - 4.12 (q, 2H, J = 14.6 Hz), 2.31 (s, 1H), 1.46 - 1.41 (t, 3H, J = 7.3 Hz). **^{13}C {^{1}H} NMR (75 MHz, $CDCl_3$)** δ 138.2, 137.3, 105.2, 43.8, 15.4, 11.0. **ESI-(+) HRMS:** *m/z* calcd for $C_6H_{10}N_2$ $[M+H]^+$, 111.0917; found 111.0920 (Δ = 2.70 ppm).

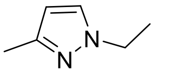

***4b**:1-Ethyl-3-methyl-1H-pyrazole*

Yield 1.2 mg (15%); brown oil; chromatography (EtOAc/hexanes = 1:10). **^{1}H NMR (300 MHz, $CDCl_3$)** δ 7.29 - 7.28 (d, 2H, J = 2.2 Hz), 6.02 (d, 2H, J = 2.2 Hz), 4.17 - 4.09 (q, 2H, J = 14.7 Hz), 2.30 (s, 3H), 1.49 - 1.45 (t, 3H, J = 7.3 Hz). **^{13}C {^{1}H} NMR (75 MHz, $CDCl_3$)** δ 148.3, 128.9, 104.8, 46.6, 15.7, 13.6. **ESI-(+) HRMS:** *m/z* calcd for $C_6H_{10}N_2$ $[M+H]^+$, 111.0917; found 111.0922 (Δ = 4.50 ppm).

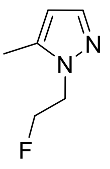

***5a**:1-(2-Fluoroethyl)-5-methyl-1H-pyrazole*

^{1}H NMR (300 MHz, $CDCl_3$) δ 7.43 (d, 1H, J = 1.5 Hz), 6.02 - 6.01 (m, 1H), 4.86 - 4.67 (d, 2H, J = 4.8 Hz), 4.39 - 4.27 (d, 2H, J = 4.8 Hz), 2.31 (s, 3H). **^{13}C {^{1}H} NMR (75 MHz, $CDCl_3$)** δ 139.3, 139.2, 105.5, 83.6, 81.3, 49.3, 49.0, 10.97, 11.0. **ESI-(+) HRMS:** *m/z* calcd for $C_6H_6FN_2$ $[M+H]^+$, 129.0823; found 129.0827 (Δ = 3.10 ppm).

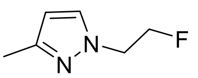

***5b**:1-(2-Fluoroethyl)-3-methyl-1H-pyrazole*

Yield 1.62 mg (7%); brown oil; chromatography (EtOAc/hexanes = 1:10). **^{1}H NMR (300 MHz, $CDCl_3$)** δ 7.35 (d, 2H, J = 2.2 Hz), 6.05 - 6.04 (d, 2H, J = 2.2 Hz), 4.82 - 4.63 (d,

2H, J = 4.7 Hz), 4.39 - 2.28 (d, 2H, J = 4.7 Hz), 1.63 (s, 3H). **^{13}C {^{1}H} NMR (75 MHz, $CDCl_3$)** δ 149.4, 131.0, 105.6, 83.3, 81.0, 52.4, 52.1, 13.7. **ESI-(+) HRMS:** *m/z* calcd for $C_6H_6FN_2$ $[M+H]^+$, 129.0823; found 129.0819 (Δ = 3.10 ppm).

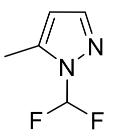

***6a**:1-(Difluoromethyl)-5-methyl-1H-pyrazole*

^{1}H NMR (300 MHz, $CDCl_3$) δ 7.49 (d, 1H, J = 1.1 Hz), 7.48 - 7.02 (t, 1H, J = 59.5 Hz), 6.15 (s, 1H), 2.48 - 2.47 (m, 2H). **^{13}C {^{1}H} NMR (75 MHz, $CDCl_3$)** δ 141.4, 139.3, 115.9, 112.6, 109.3, 109.2, 10.8. **ESI-(+) HRMS:** *m/z* calcd for $C_5H_6F_2N_2$ $[M+H]^+$, 133.0572; found 133.0568 (Δ = 3.01 ppm).

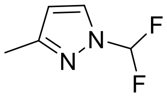

***6b**:1-(Difluoromethyl)-3-methyl-1H-pyrazole*

Yield 1.56 mg (6%); brown oil; chromatography (EtOAc/hexanes = 1:10). **^{1}H NMR (300 MHz, $CDCl_3$)** δ 7.70 - 7.69 (d, 1H, J = 2.6 Hz), 7.31 - 6.91 (t, 1H, J = 61.0 Hz), 6.24 - 6.23 (d, 1H, J = 2.6 Hz), 2.31 (s, 3H). **^{13}C {^{1}H} NMR (75 MHz)** δ 151.6, 127.3, 114.1, 110.8, 108.9, 107.5, 13.6. **ESI-(+) HRMS:** *m/z* calcd for $C_5H_6F_2N_2$ $[M+H]^+$, 133.0572; found 133.0578 (Δ = 4.51 ppm).

^{1}H NMR, ^{13}C NMR spectra and HRMS

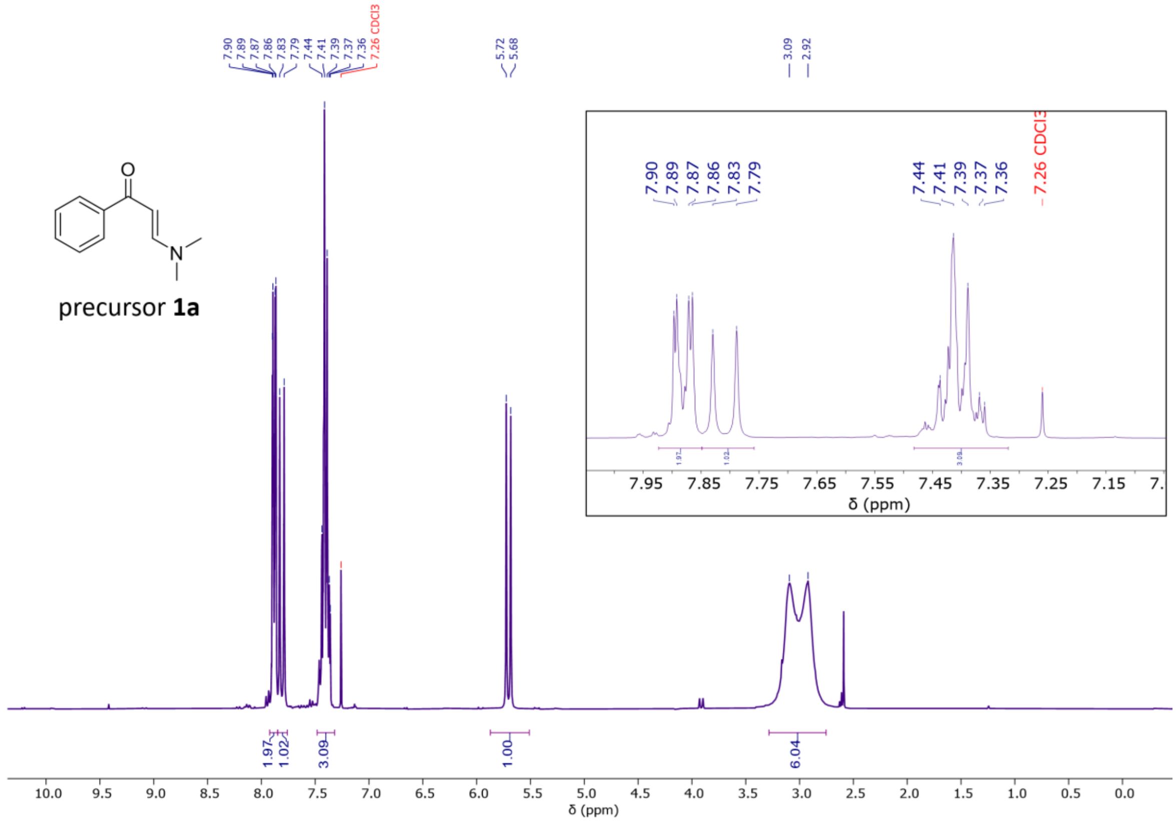

Figure S25. ^{1}H NMR spectrum of compound (*2E*)-3-(Dimethylamino)-1-phenyl-2-propen-1-one.

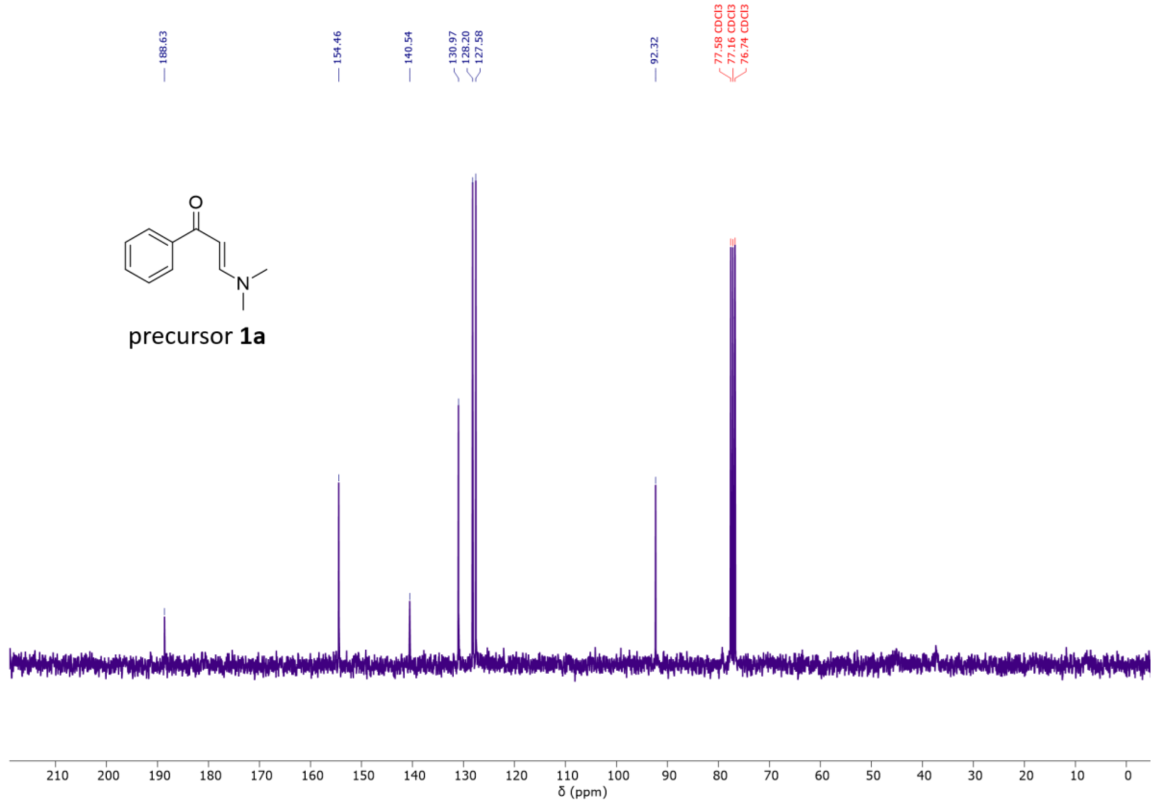

Figure S26. ^{13}C {^{1}H} NMR spectrum of compound (*2E*)-3-(Dimethylamino)-1-phenyl-2-propen-1-one.

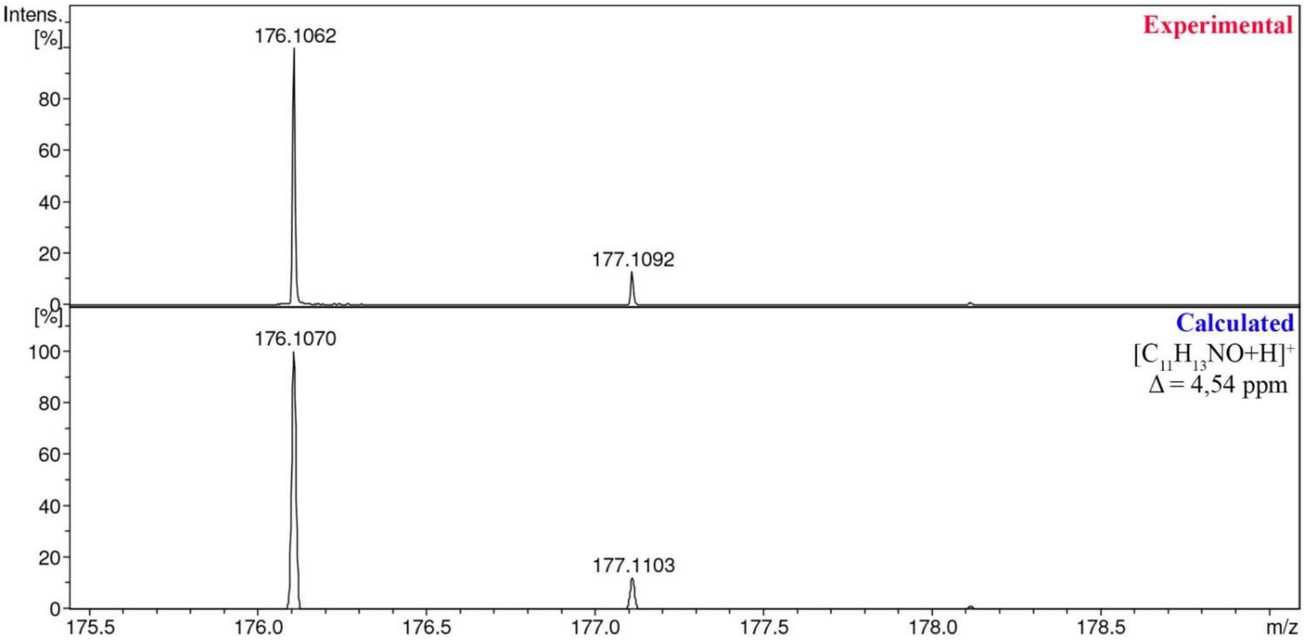

Figure S27. ESI-HRMS of compound (*2E*)-3-(Dimethylamino)-1-phenyl-2-propen-1-one.

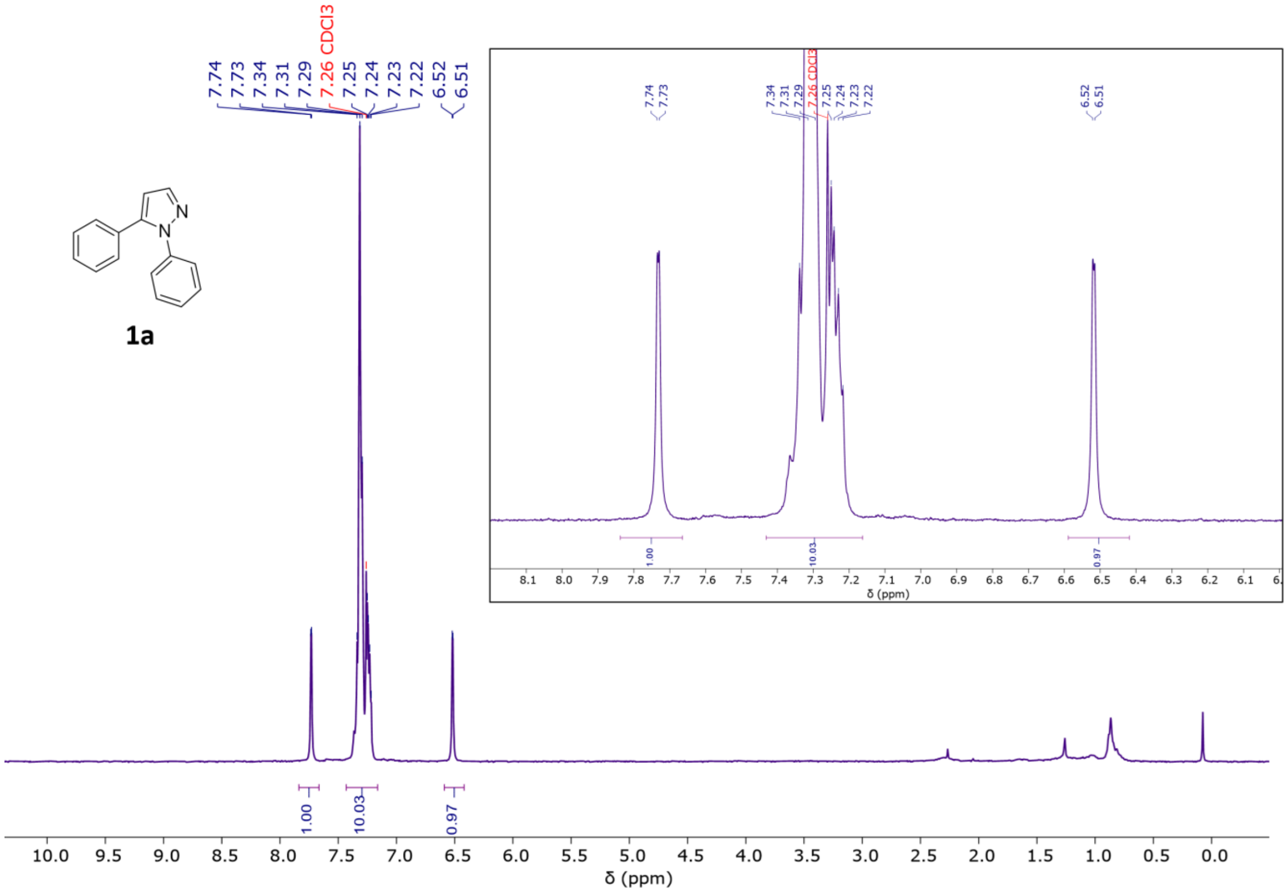

Figure S28. ^{1}H NMR spectrum of compound 1,5-Diphenyl-*1H*-pyrazole.

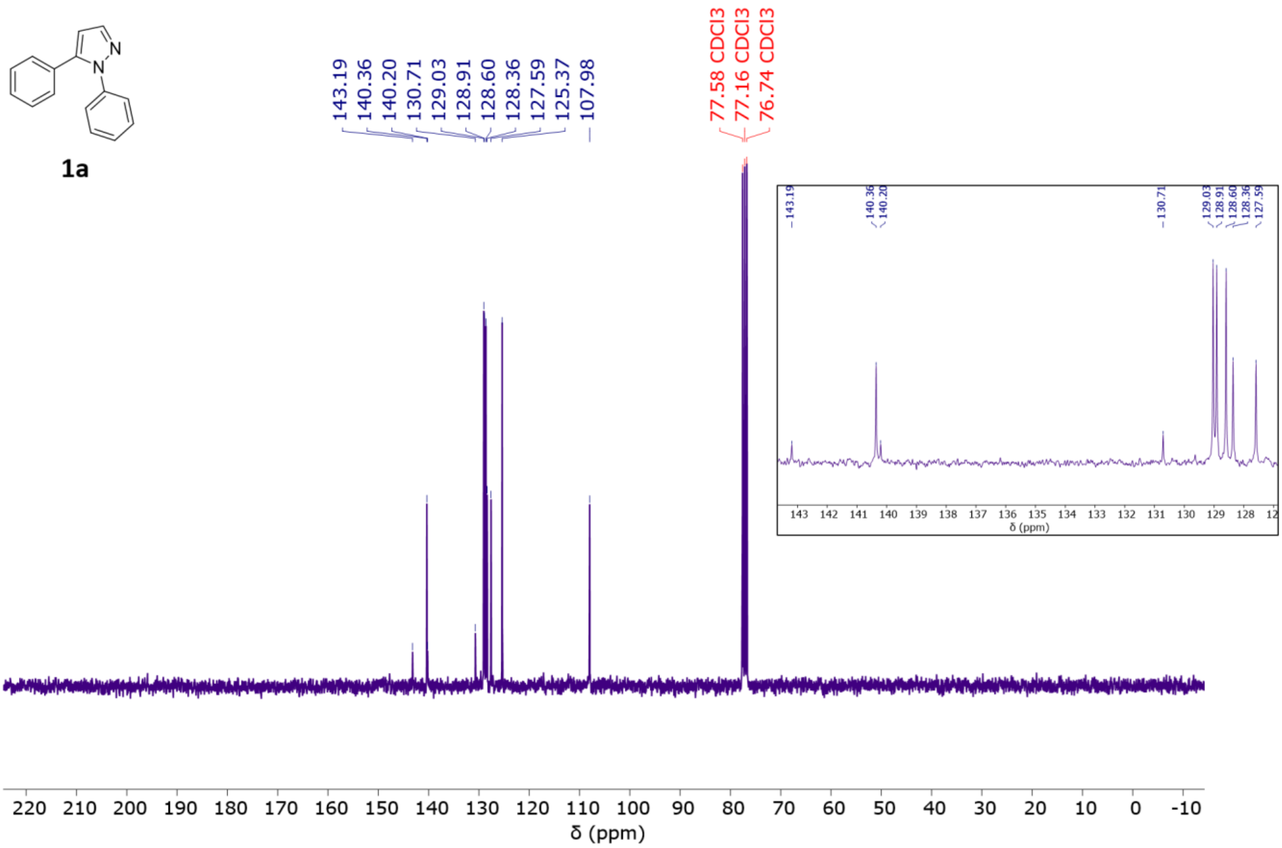

Figure S29. ^{13}C {^{1}H} NMR spectrum of compound 1,5-Diphenyl-*1H*-pyrazole.

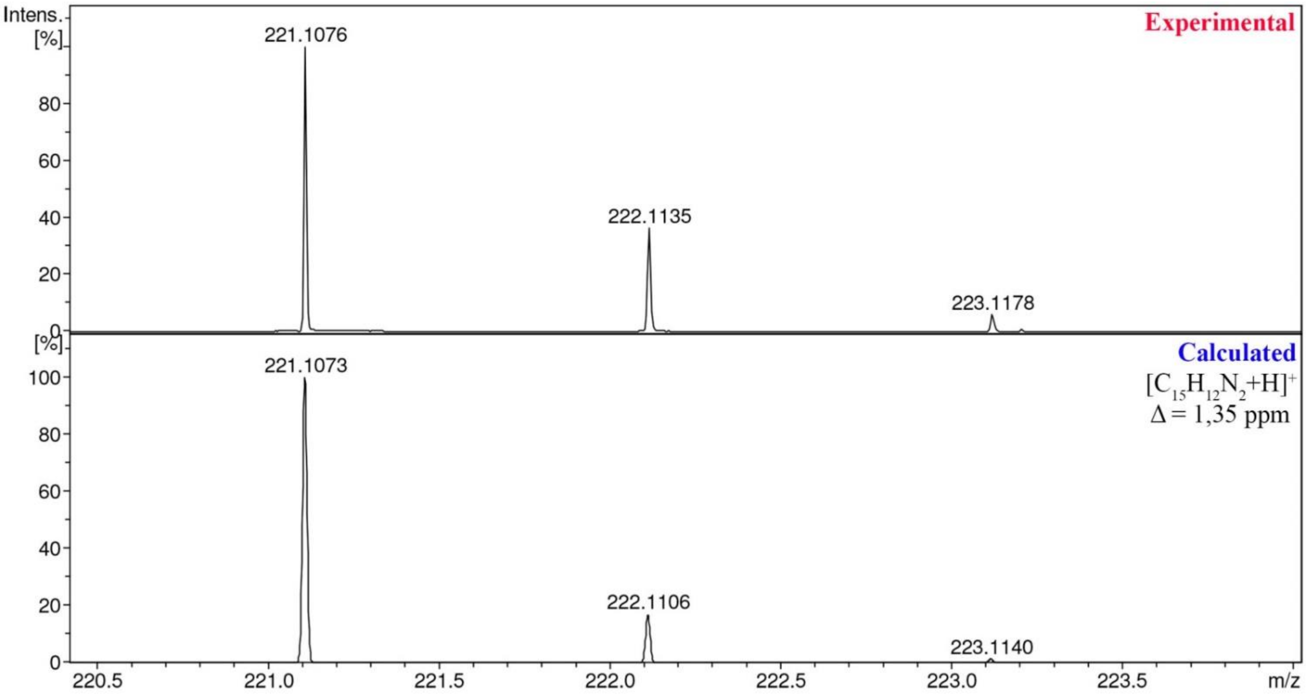

Figure S30. ESI-HRMS of compound 1,5-Diphenyl-*1H*-pyrazole.

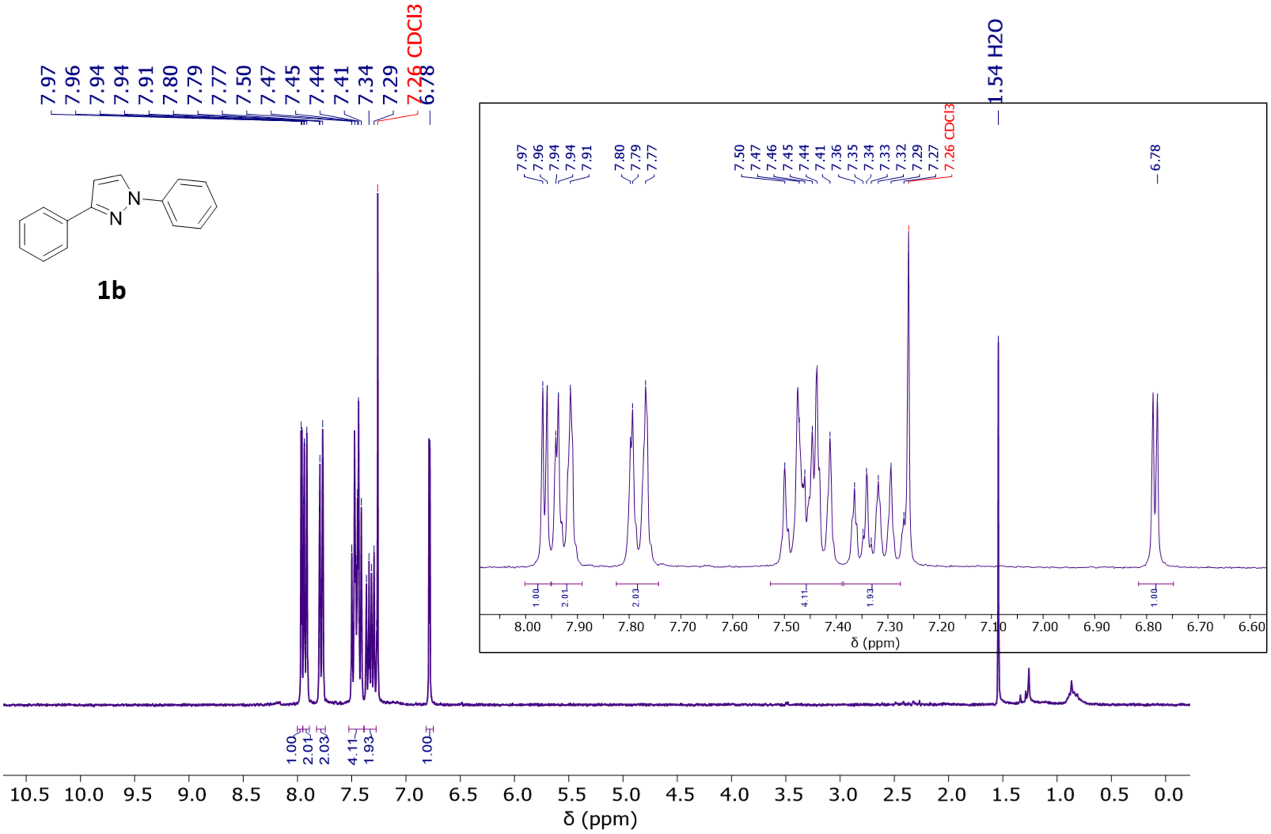

Figure S31. ^{1}H NMR spectrum of compound 1,3-Diphenyl-*1H*-pyrazole.

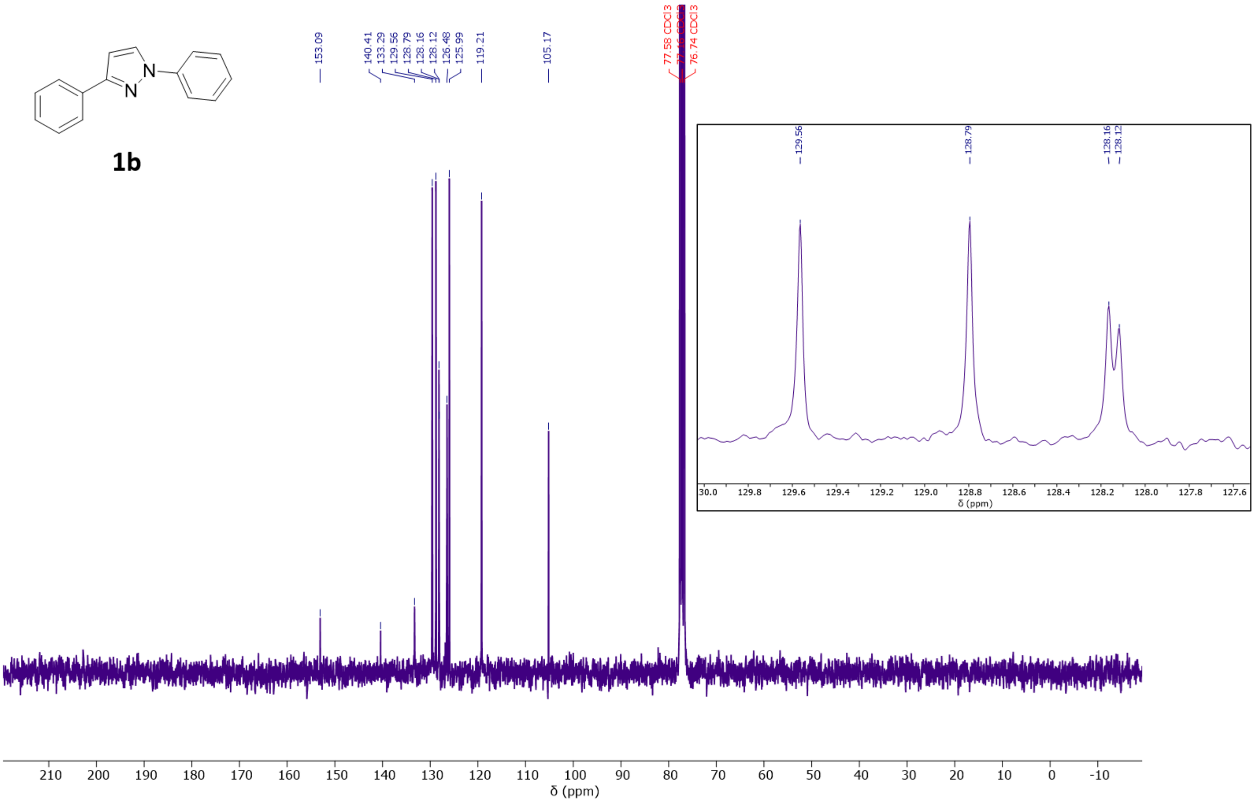

Figure S32. ^{13}C {^{1}H} NMR spectrum of compound 1,3-Diphenyl-*1H*-pyrazole.

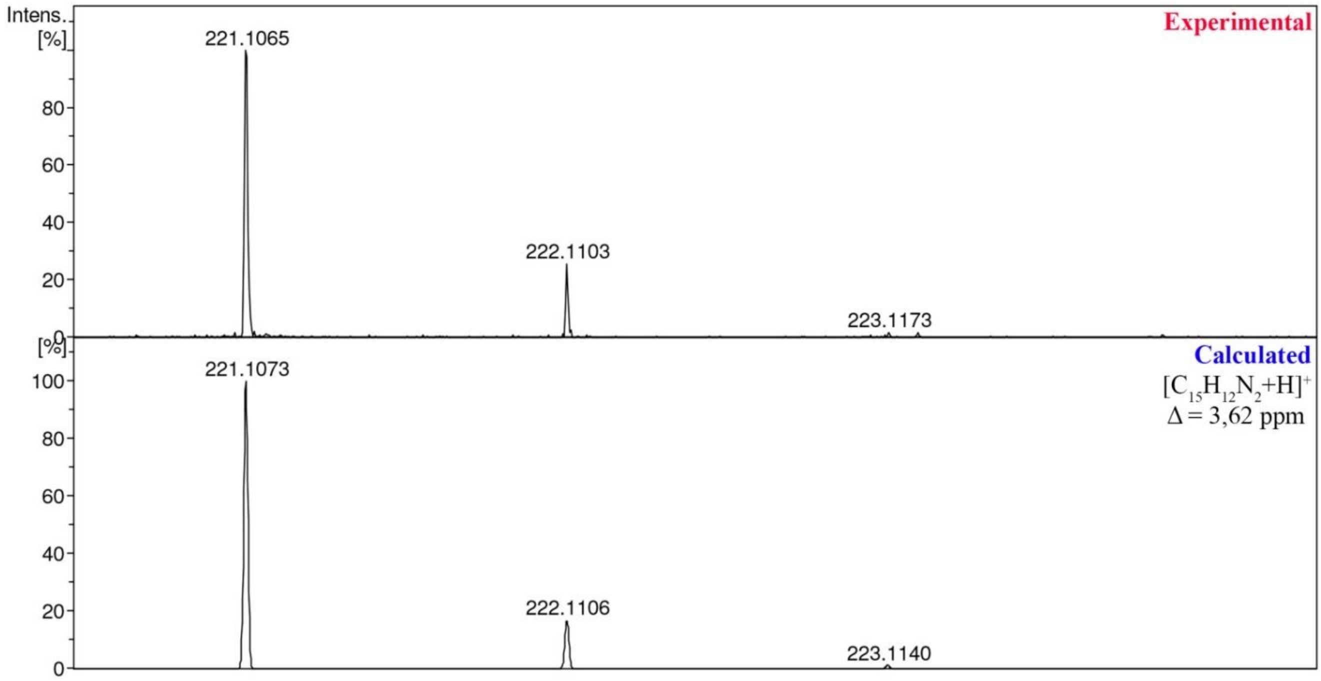

Figure S33. ESI-HRMS of compound 1,3-Diphenyl-*1H*-pyrazole.

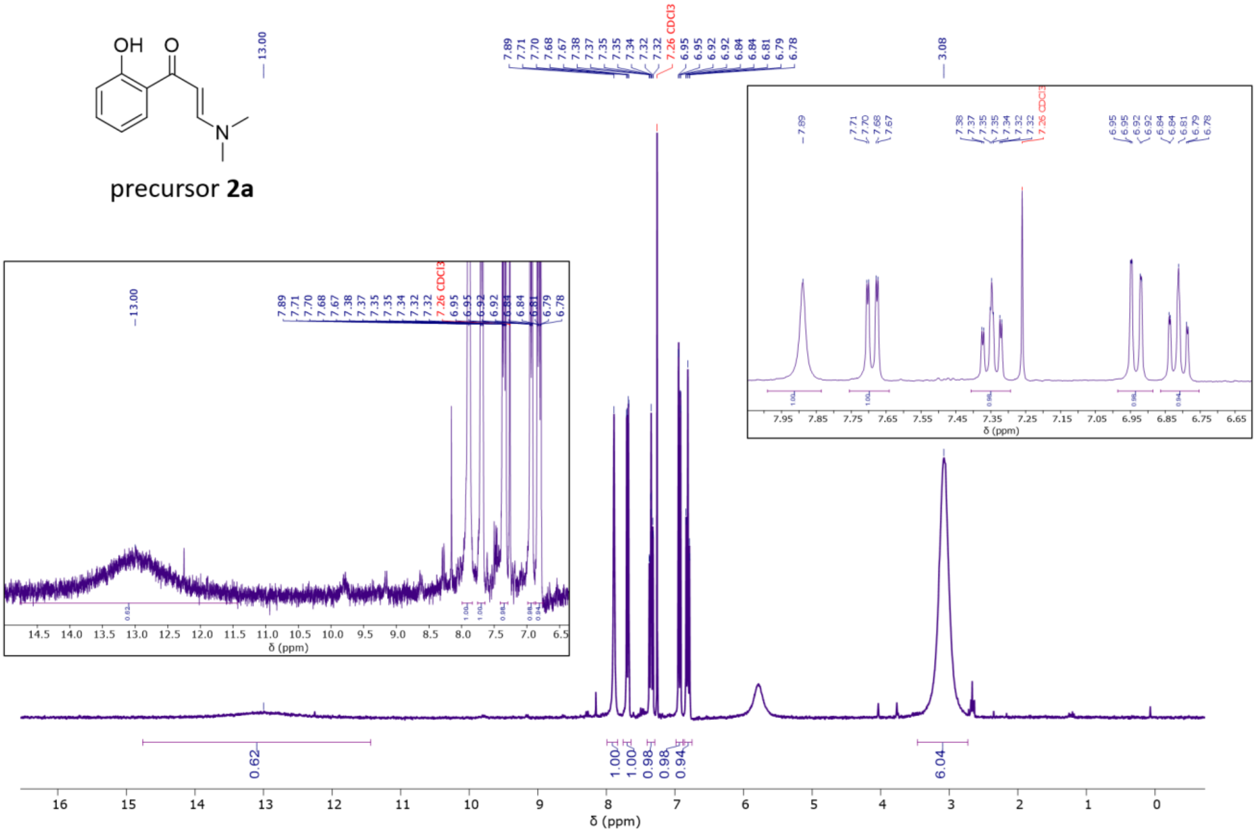

Figure S34. ^{1}H NMR spectrum of compound (*2E*)-3-(Dimethylamino)-1-(2-hydroxyphenyl)-2-propen-1-one.

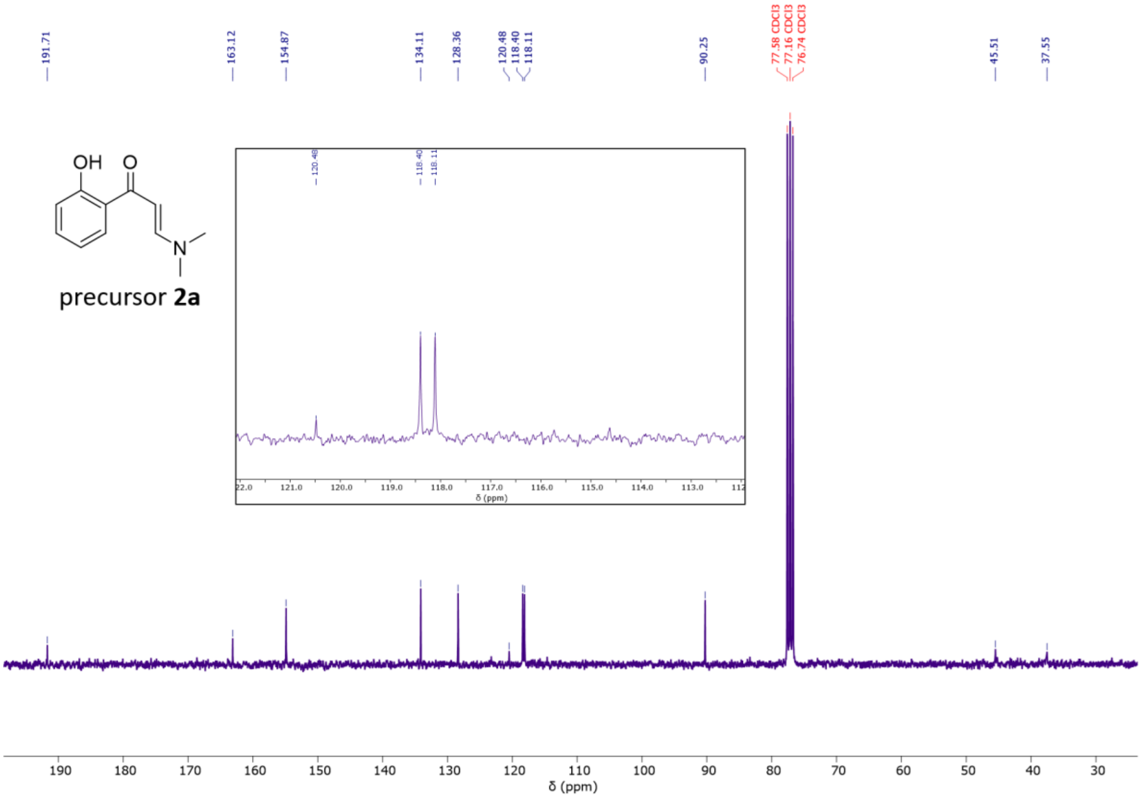

Figure S35. ^{13}C {^{1}H} NMR spectrum of compound (*2E*)-3-(Dimethylamino)-1-(2-hydroxyphenyl)-2-propen-1-one.

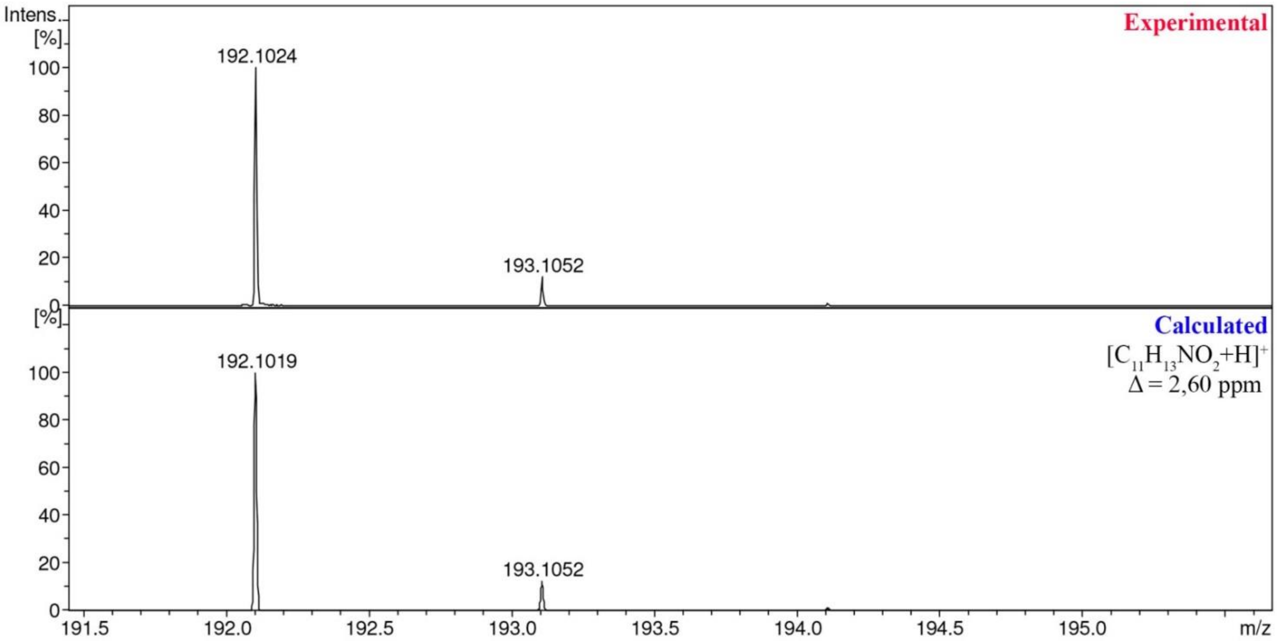

Figure S36. ESI-HRMS of compound (*2E*)-3-(Dimethylamino)-1-(2-hydroxyphenyl)-2-propen-1-one.

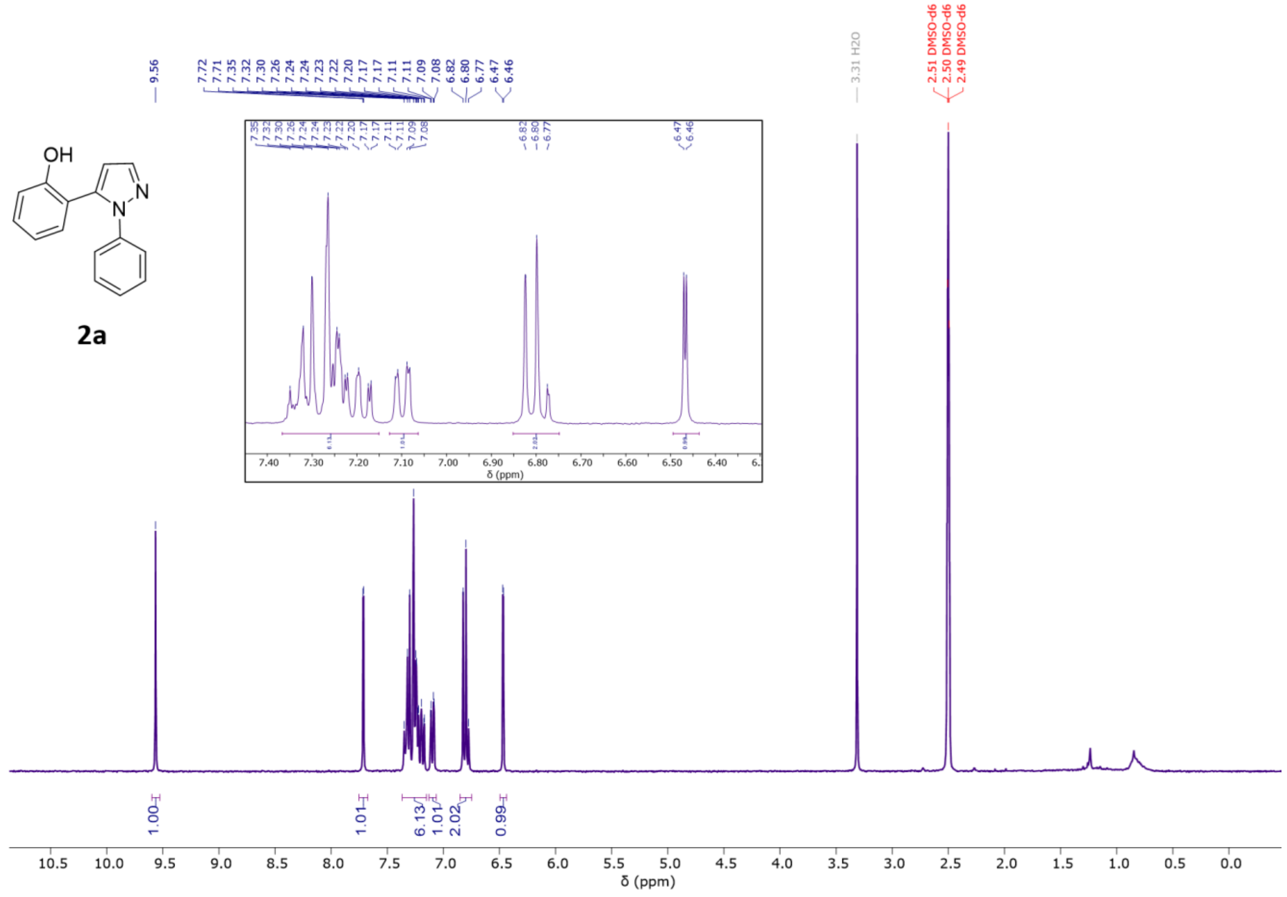

Figure S37. ^{1}H NMR spectrum of compound 2-(1-Phenyl-1*H*-pyrazol-5-yl)phenol.

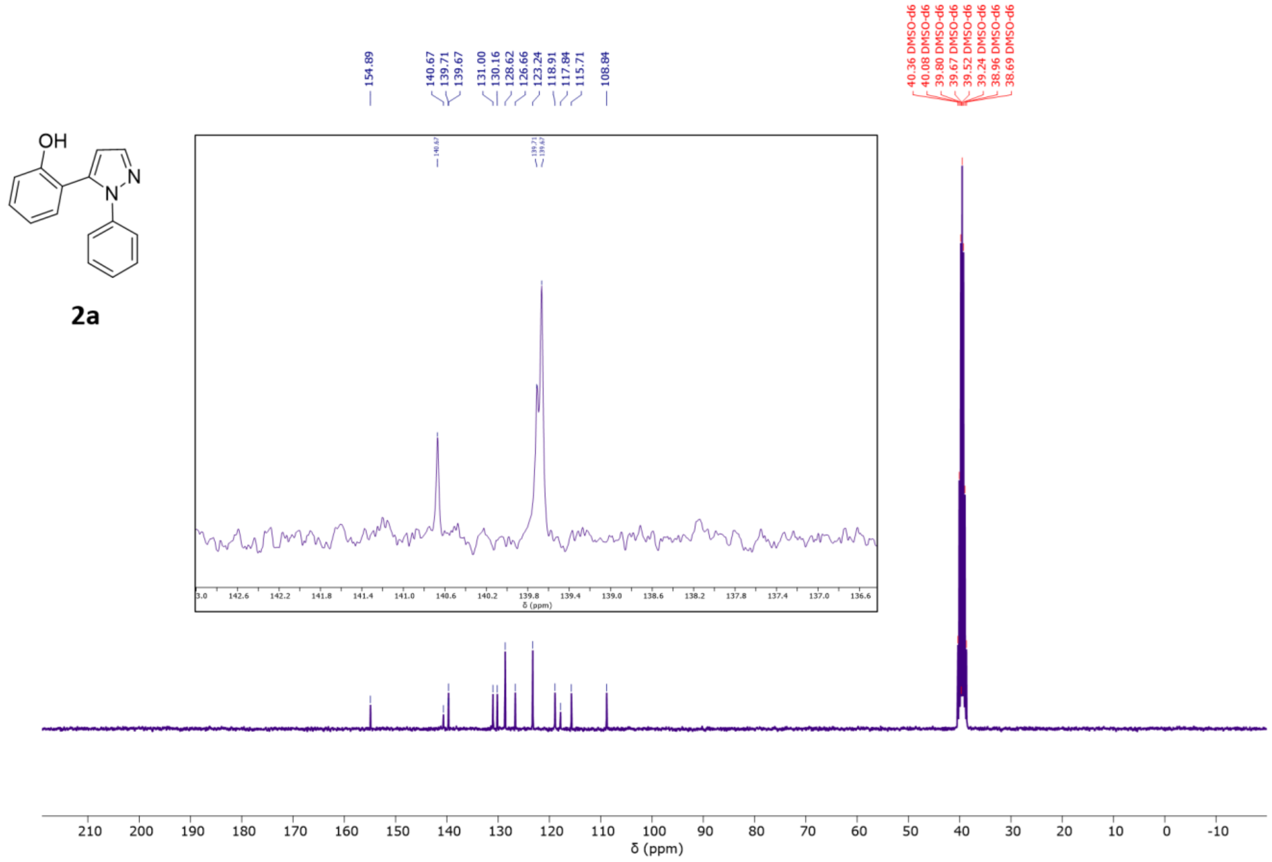

Figure S38. ^{13}C {^{1}H} NMR spectrum of compound 2-(1-Phenyl-1*H*-pyrazol-5-yl)phenol.

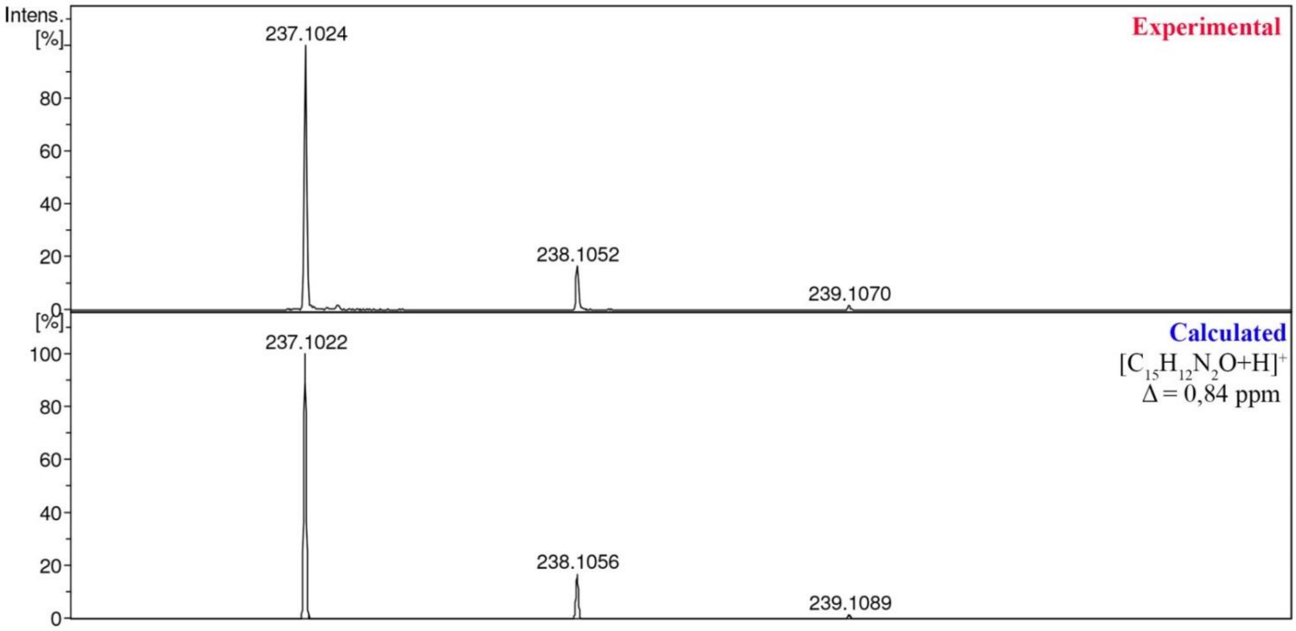

Figure S39. ESI-HRMS of compound 2-(1-Phenyl-1*H*-pyrazol-5-yl)phenol.

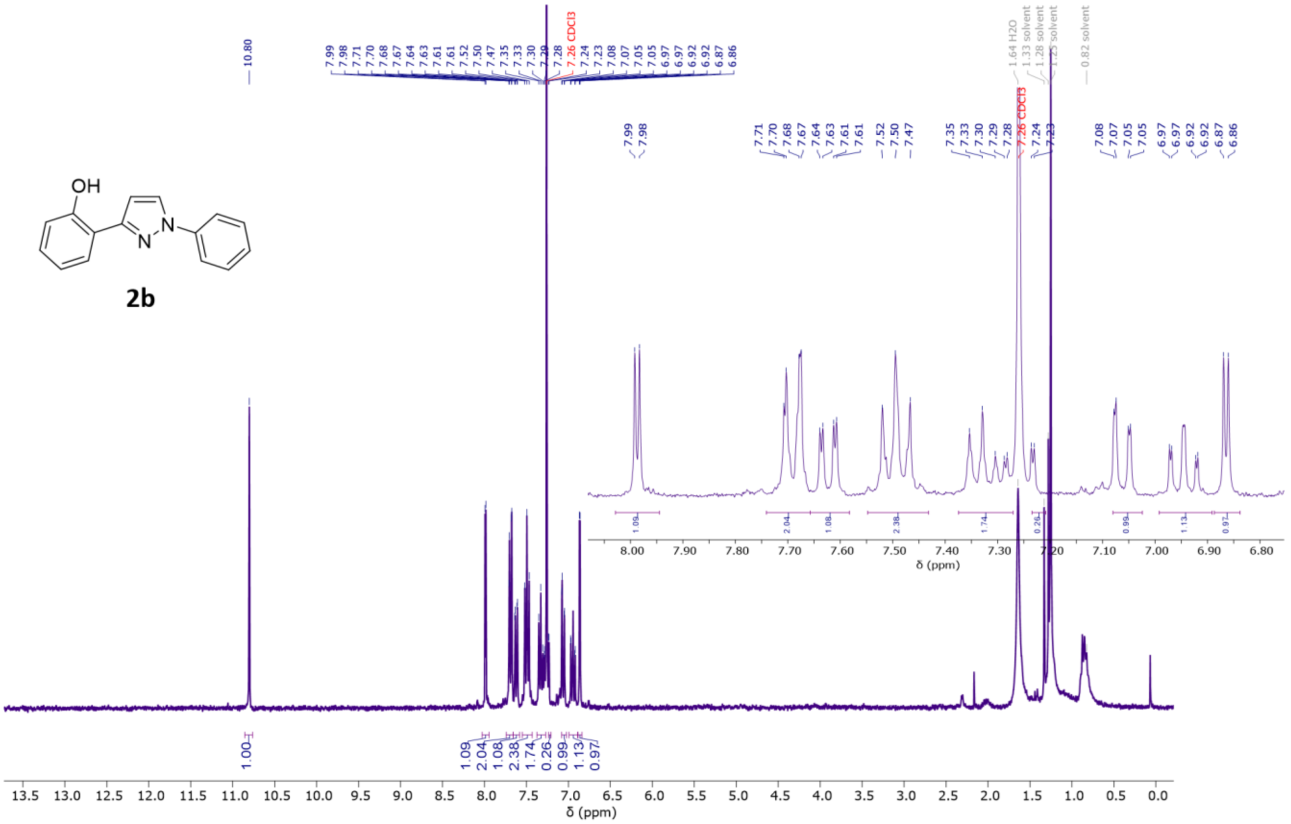

Figure S40. ^{1}H NMR spectrum of compound 2-(1-Phenyl-1*H*-pyrazol-3-yl)phenol.

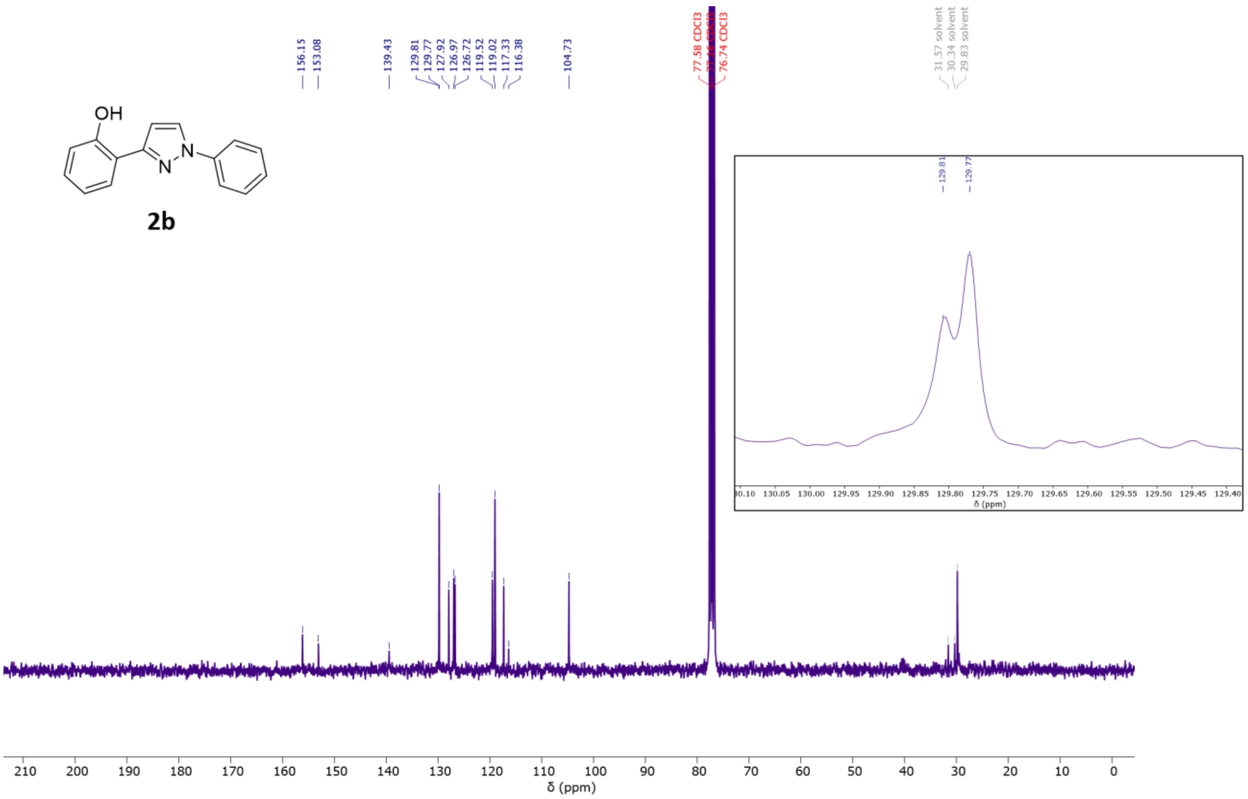

Figure S41. ^{13}C {^{1}H} NMR spectrum of compound 2-(1-Phenyl-1*H*-pyrazol-3-yl)phenol.

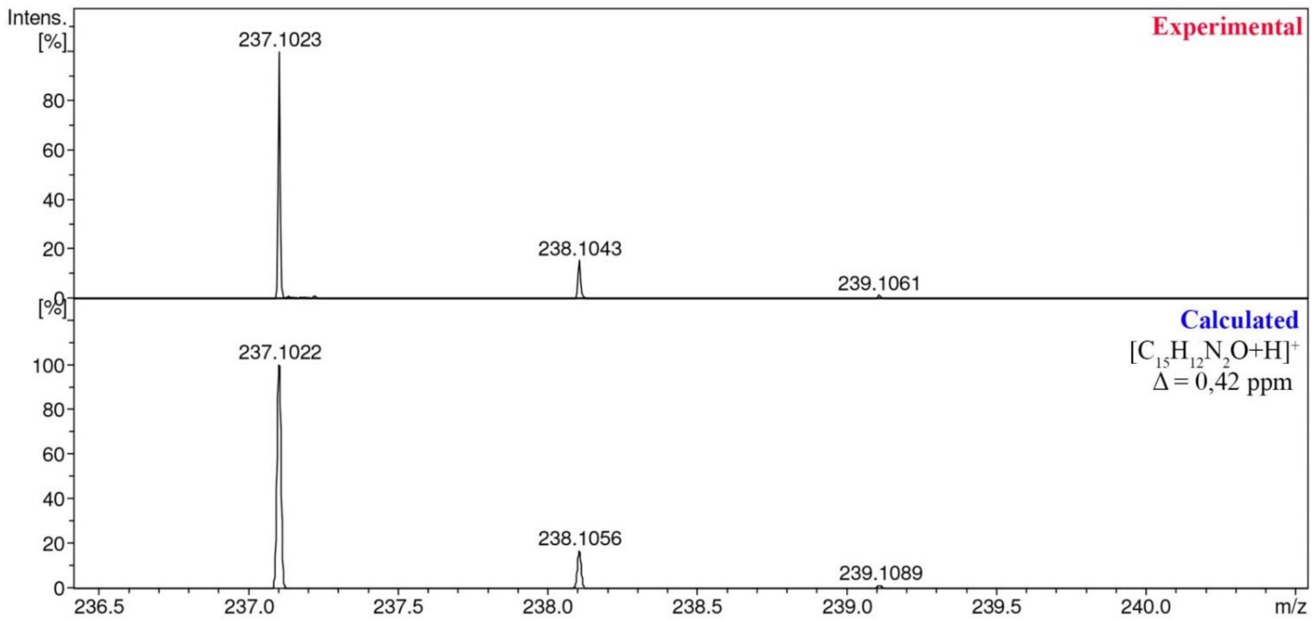

Figure S42. ESI-HRMS of compound 2-(1-Phenyl-1*H*-pyrazol-3-yl)phenol.

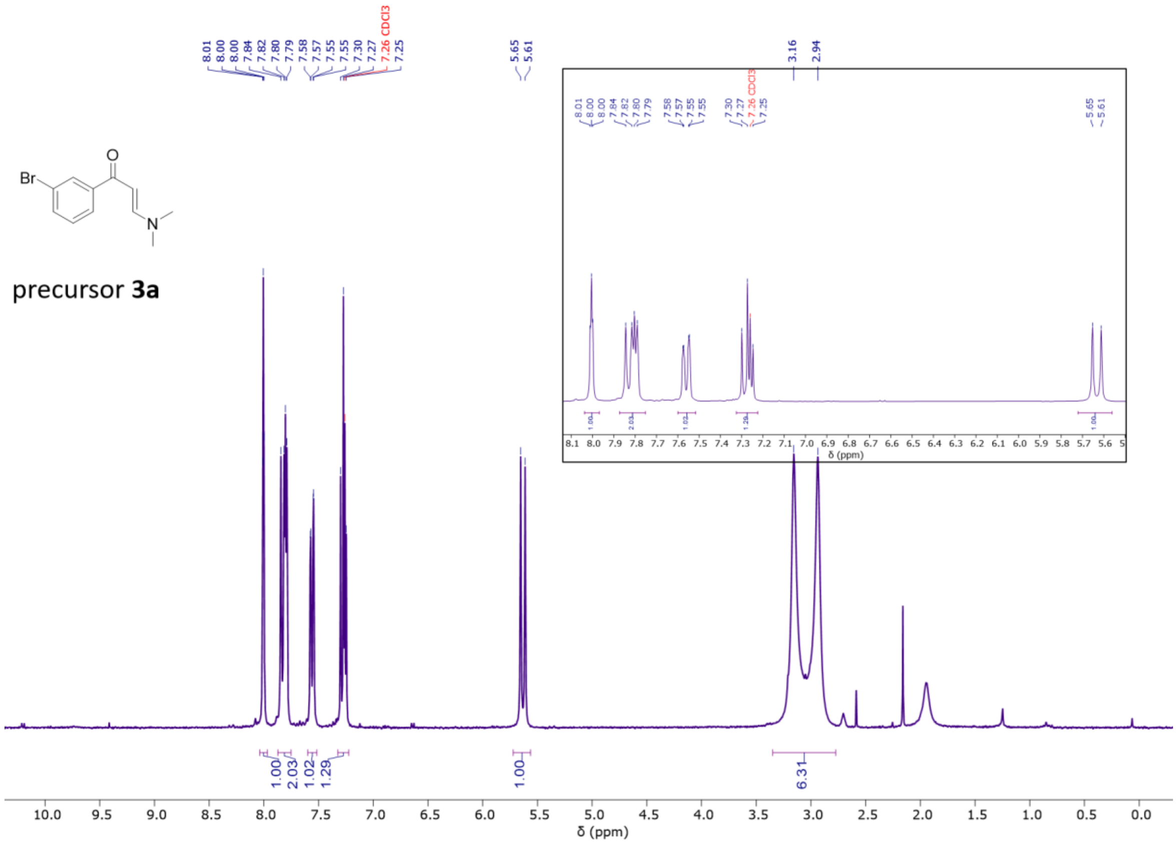

Figure S43. ^{1}H NMR spectrum of compound (*2E*)-1-(3-bromophenyl)-3-(dimethylamino)-2-propen-1-one.

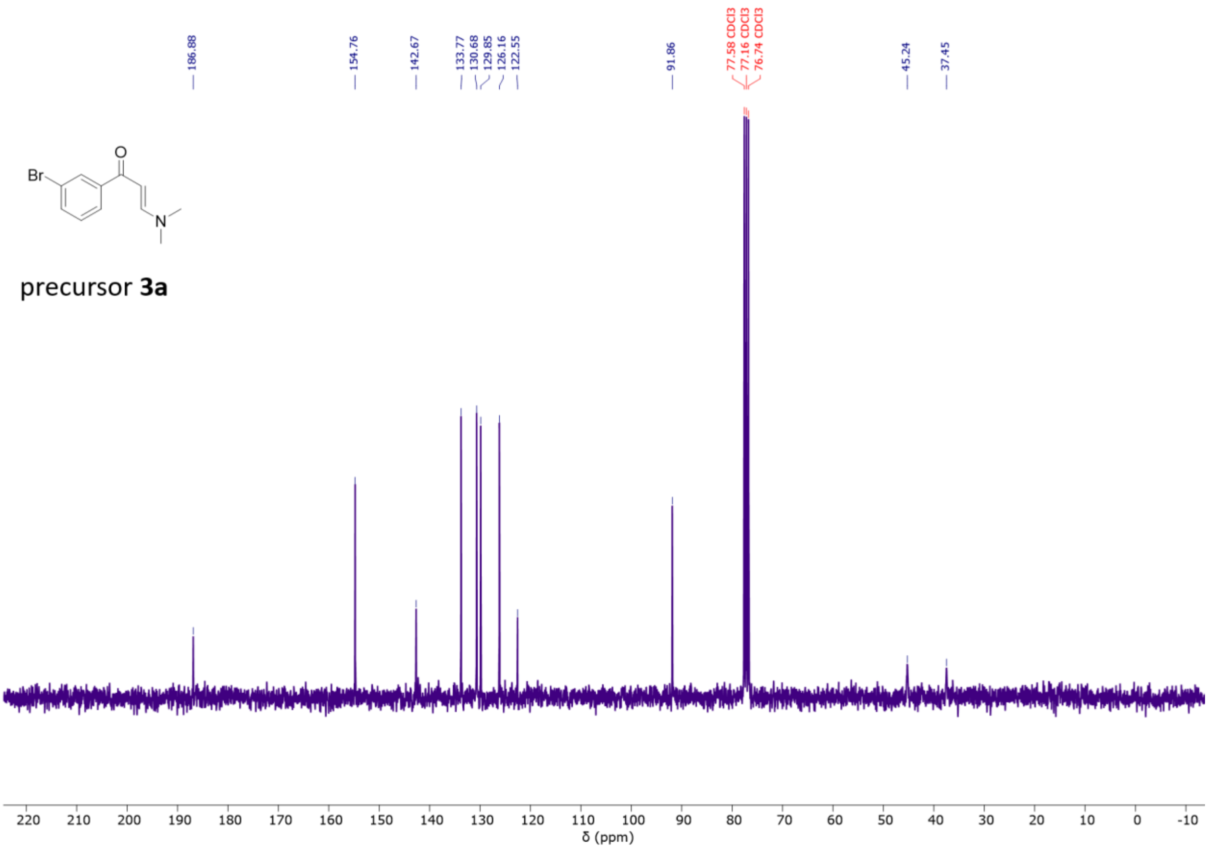

Figure S44. ^{13}C {^{1}H} NMR spectrum of compound (*2E*)-1-(3-bromophenyl)-3-(dimethylamino)-2-propen-1-one.

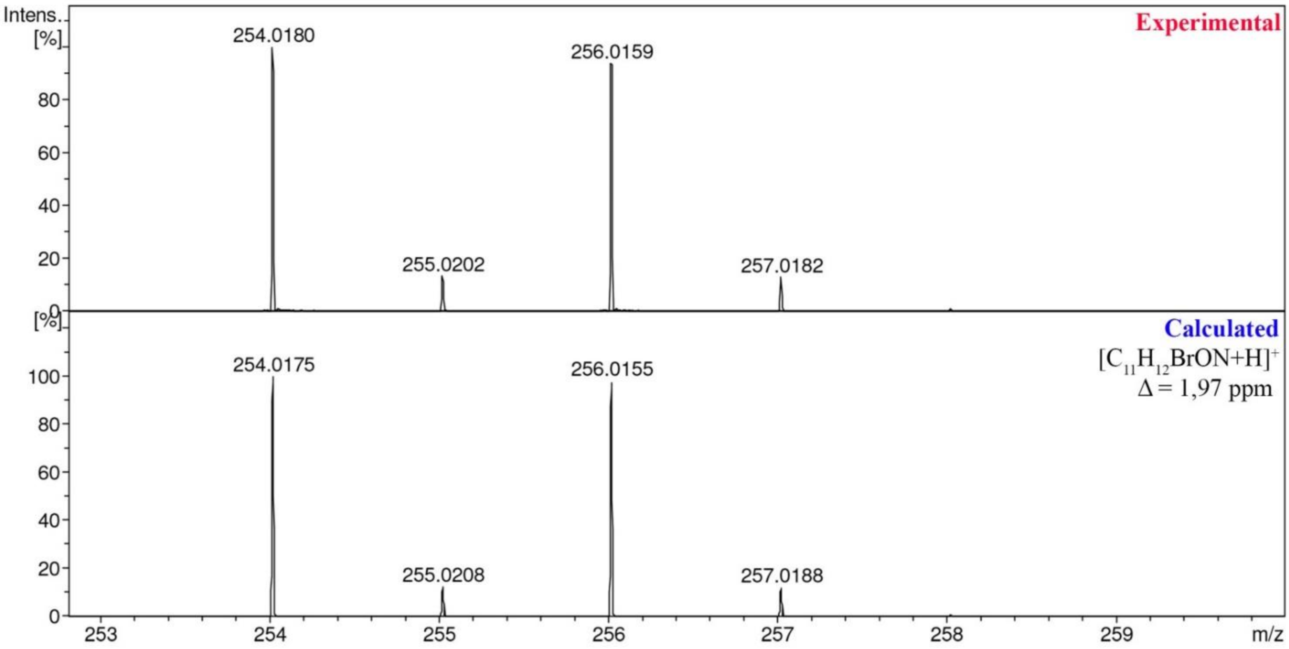

Figure S45. ESI-HRMS of compound (*2E*)-1-(3-bromophenyl)-3-(dimethylamino)-2-propen-1-one.

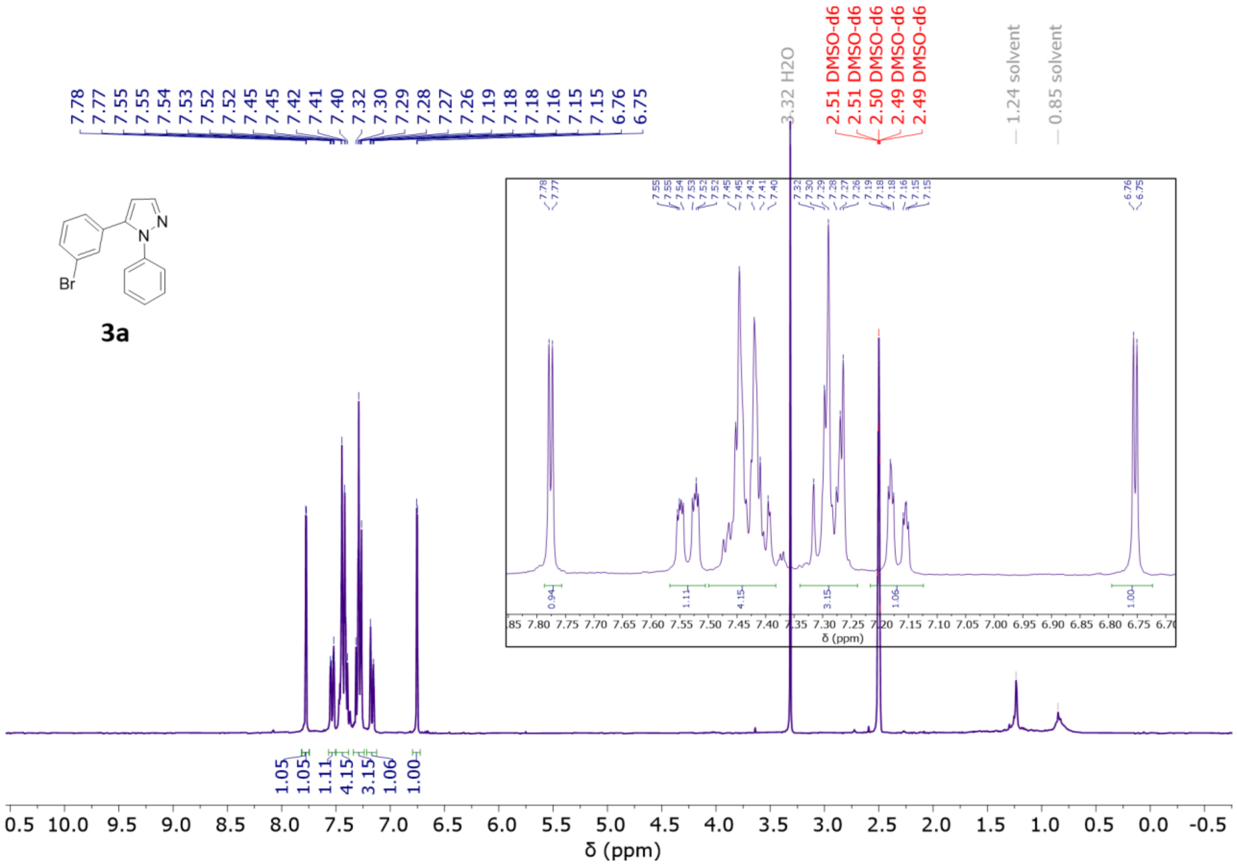

Figure S46. ^{1}H NMR spectrum of the compound 5-(3-bromophenyl)-1-phenyl-*1H*-pyrazole.

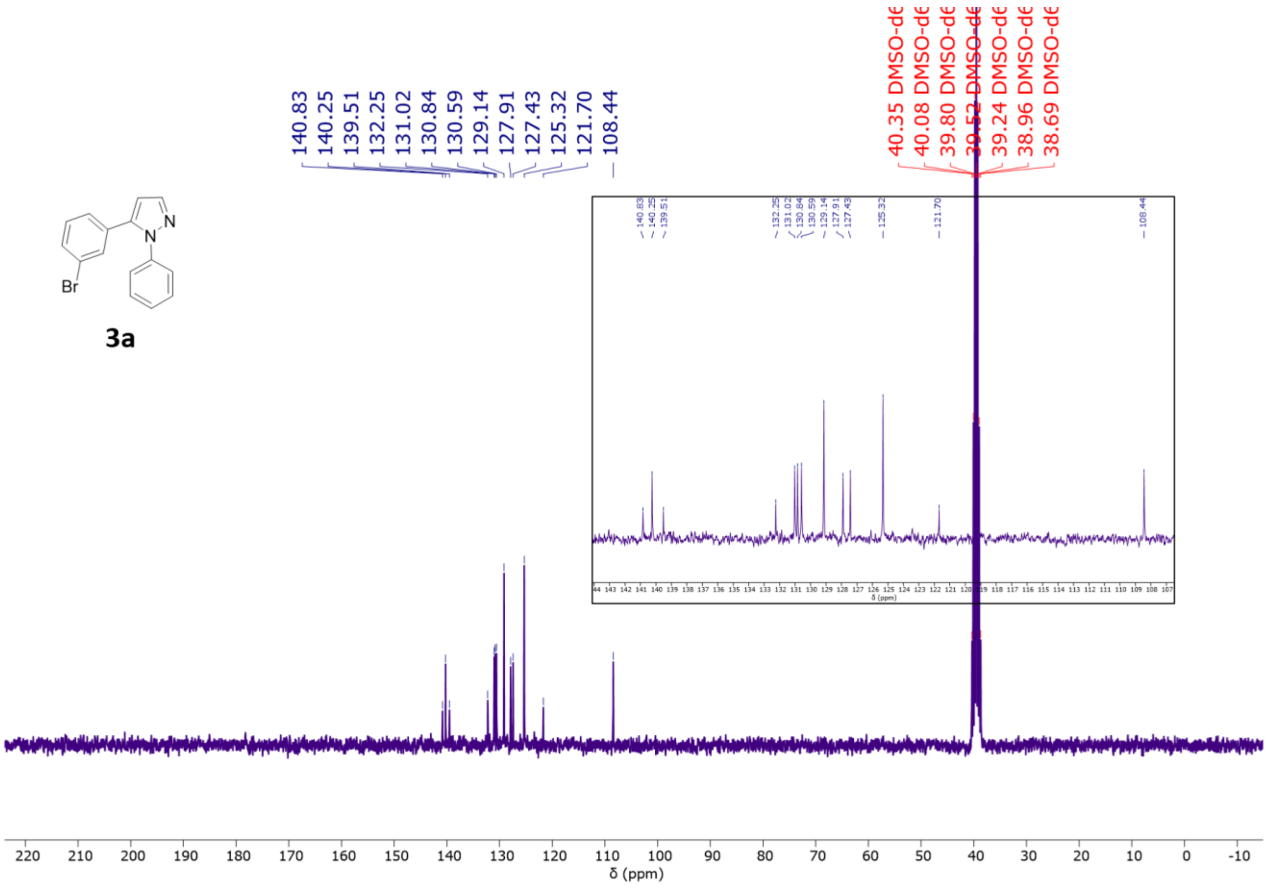

Figure S47. ^{13}C {^{1}H} NMR spectrum of the compound 5-(3-bromophenyl)-1-phenyl-*1H*-pyrazole.

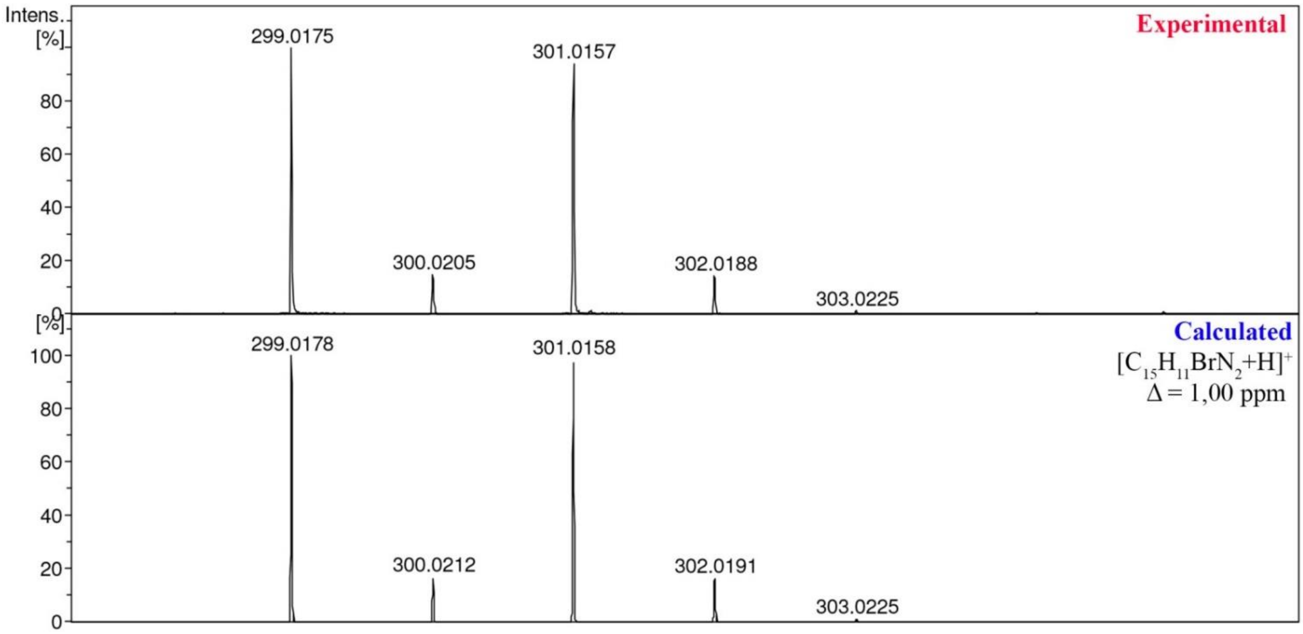

Figure S48. ESI-HRMS of the compound 5-(3-bromophenyl)-1-phenyl-*1H*-pyrazole.

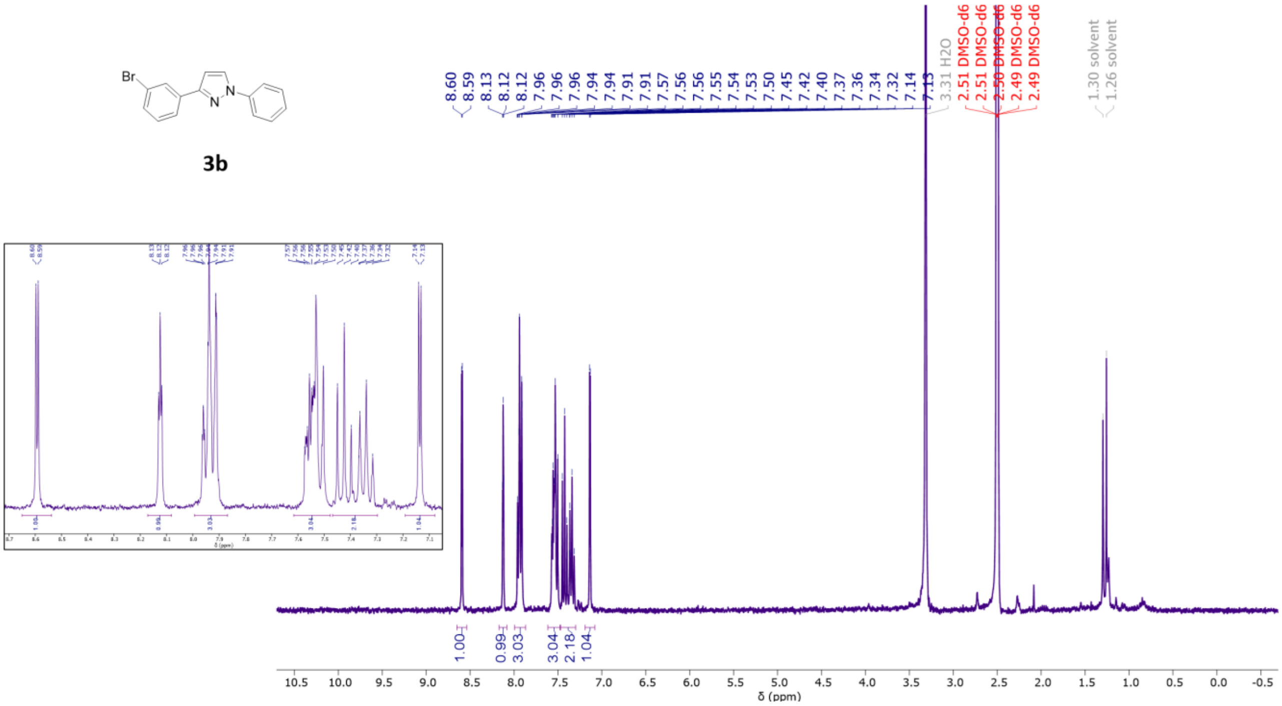

Figure S49. ^{1}H NMR spectrum of 3-(3-bromophenyl)-1-phenyl-*1H*-pyrazole.

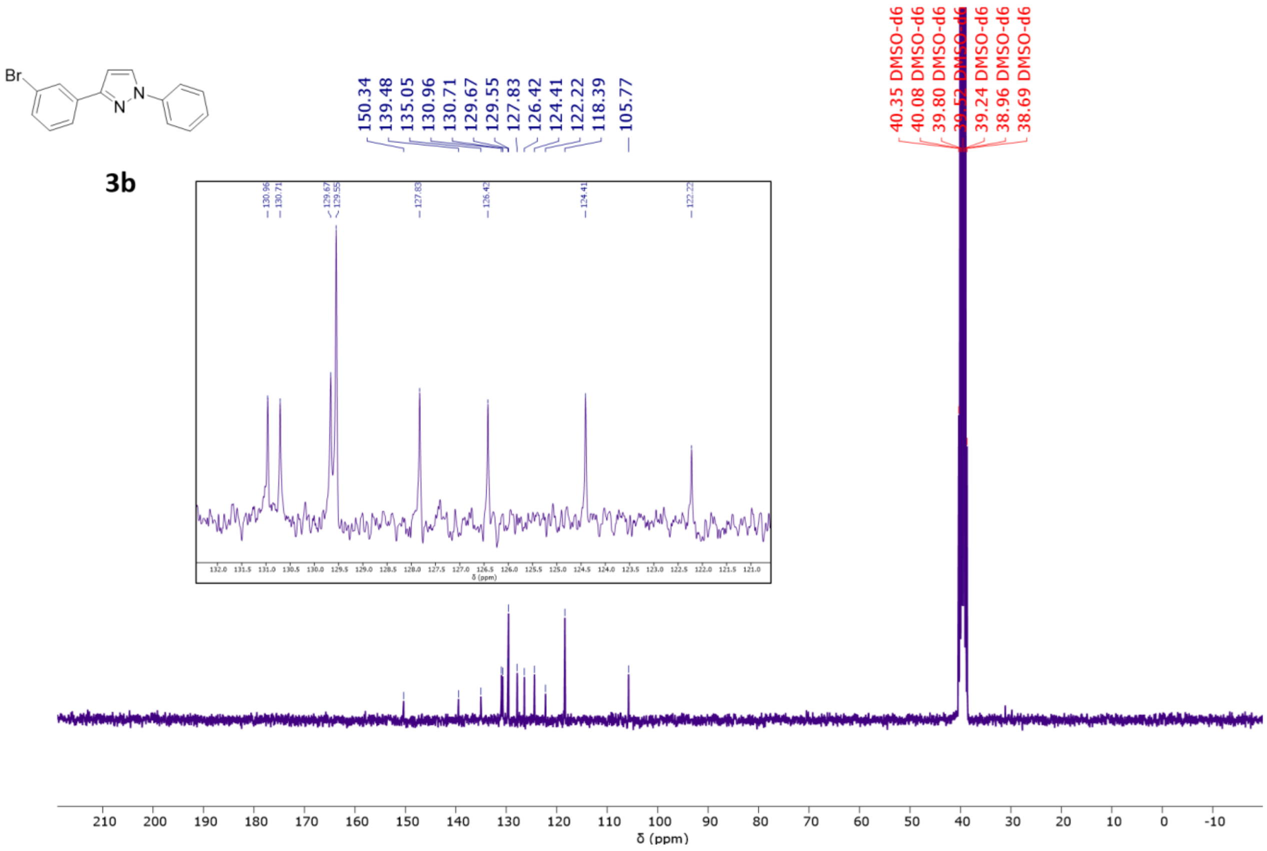

Figure S50. ^{13}C {^{1}H} NMR spectrum of 3-(3-bromophenyl)-1-phenyl-*1H*-pyrazole.

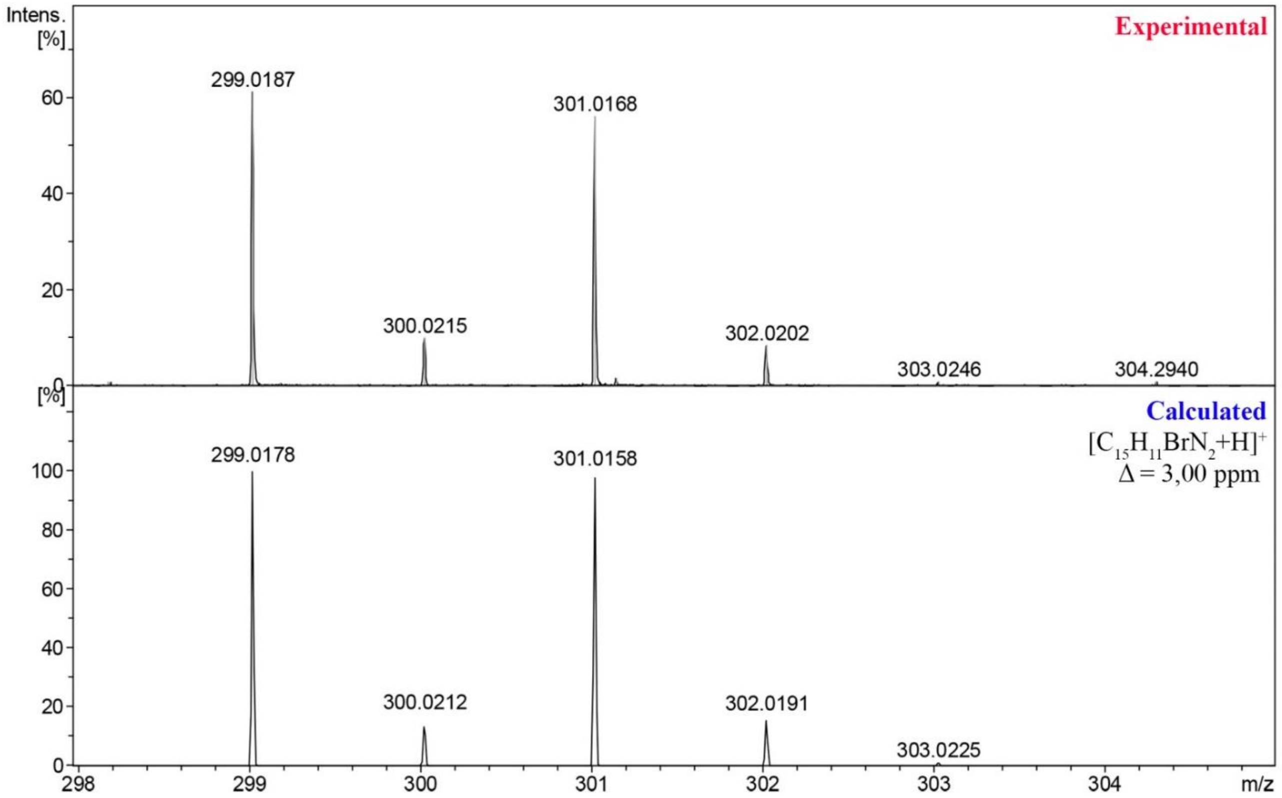

Figure S51. ESI-HRMS of compound 3-(3-Bromophenyl)-1-phenyl-*1H*-pyrazole.

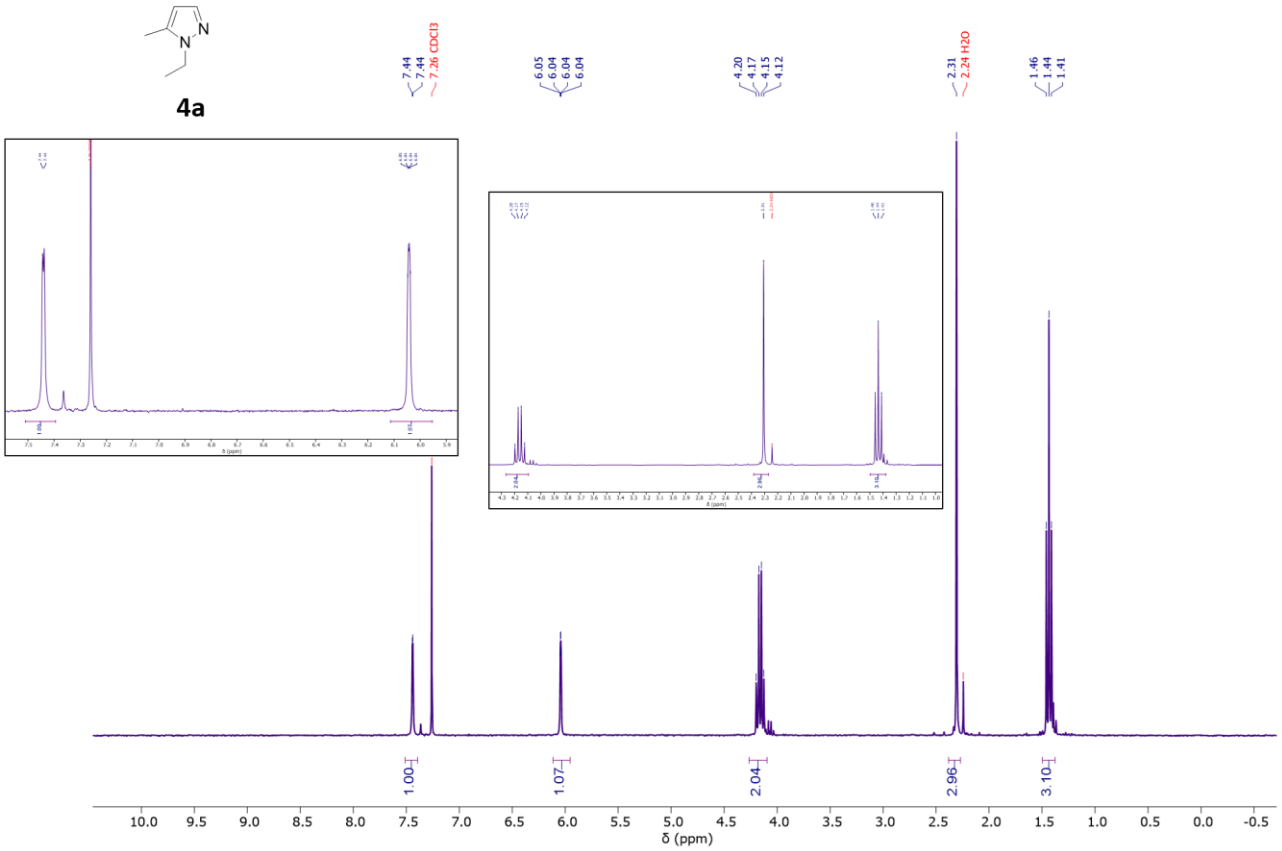

Figure S52. ^{1}H NMR spectrum of compound 1-ethyl-5-methyl-*1H*-pyrazole.

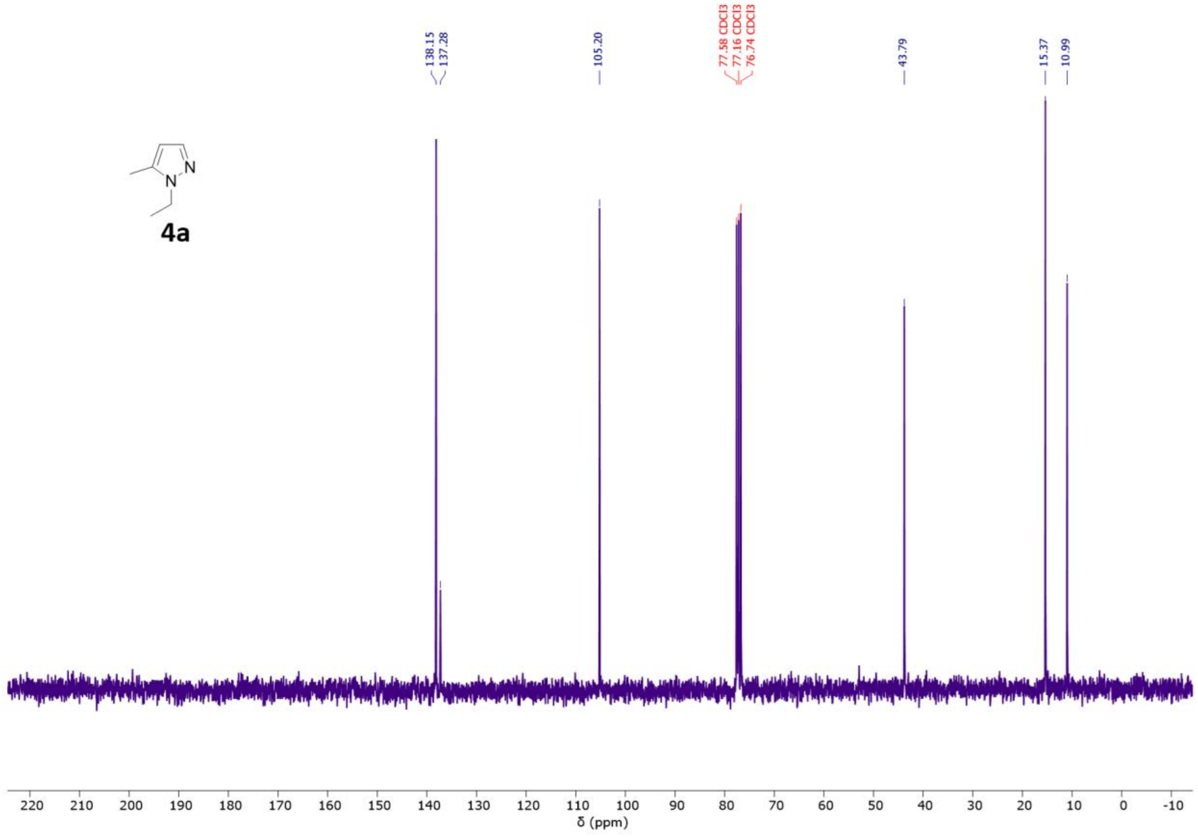

Figure S53. ^{13}C {^{1}H} NMR spectrum of compound 1-ethyl-5-methyl-*1H*-pyrazole.

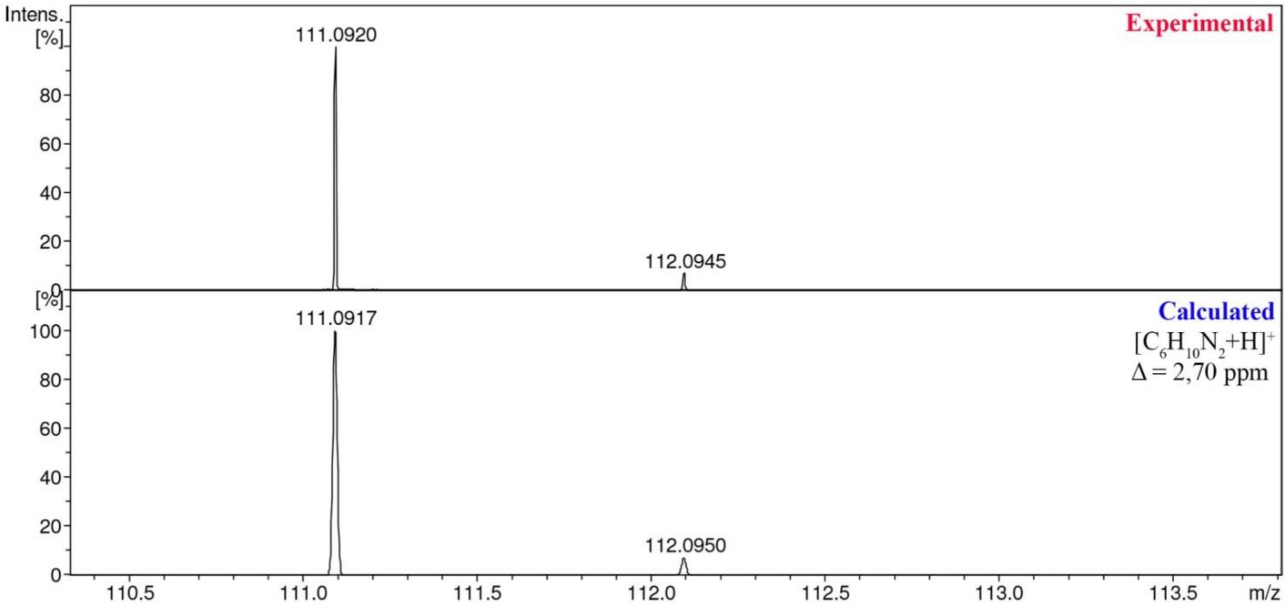

Figure S54. ESI-HRMS of compound 1-ethyl-5-methyl-*1H*-pyrazole.

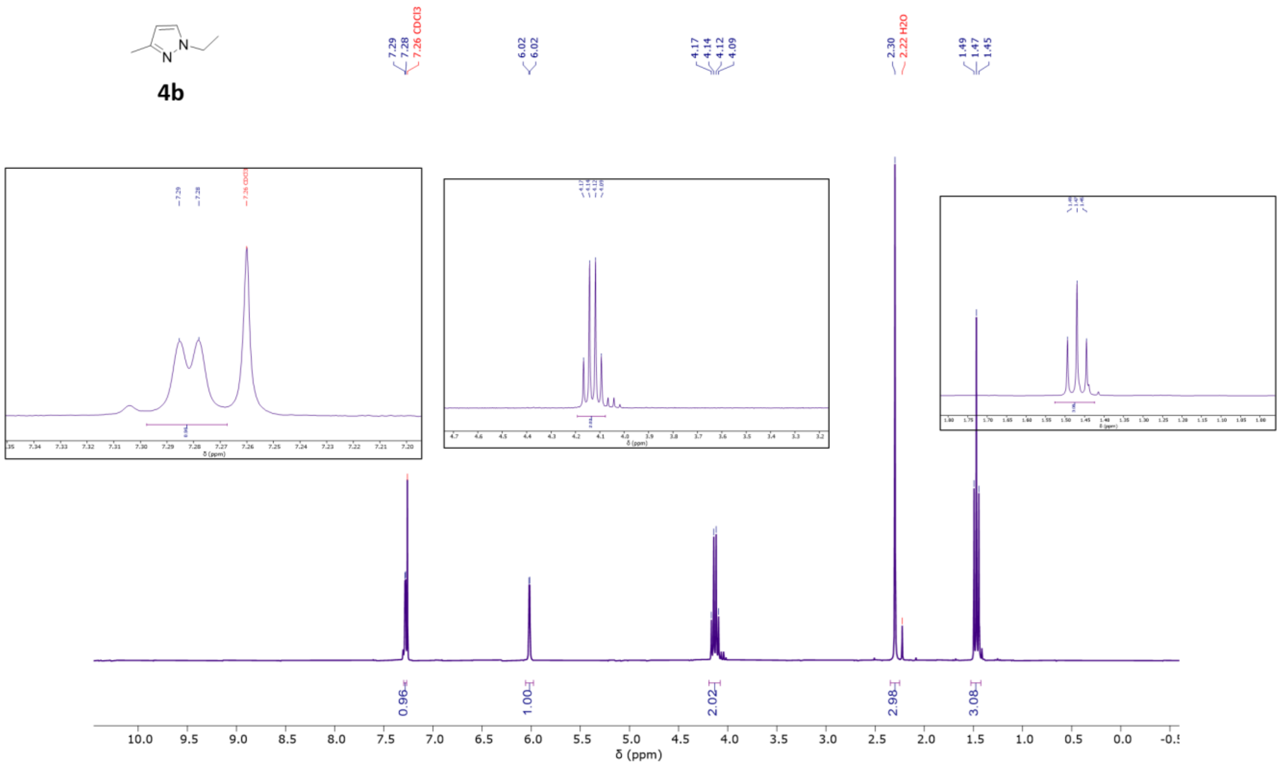

Figure S55. ^{1}H NMR spectrum of compound 1-ethyl-3-methyl-*1H*-pyrazole.

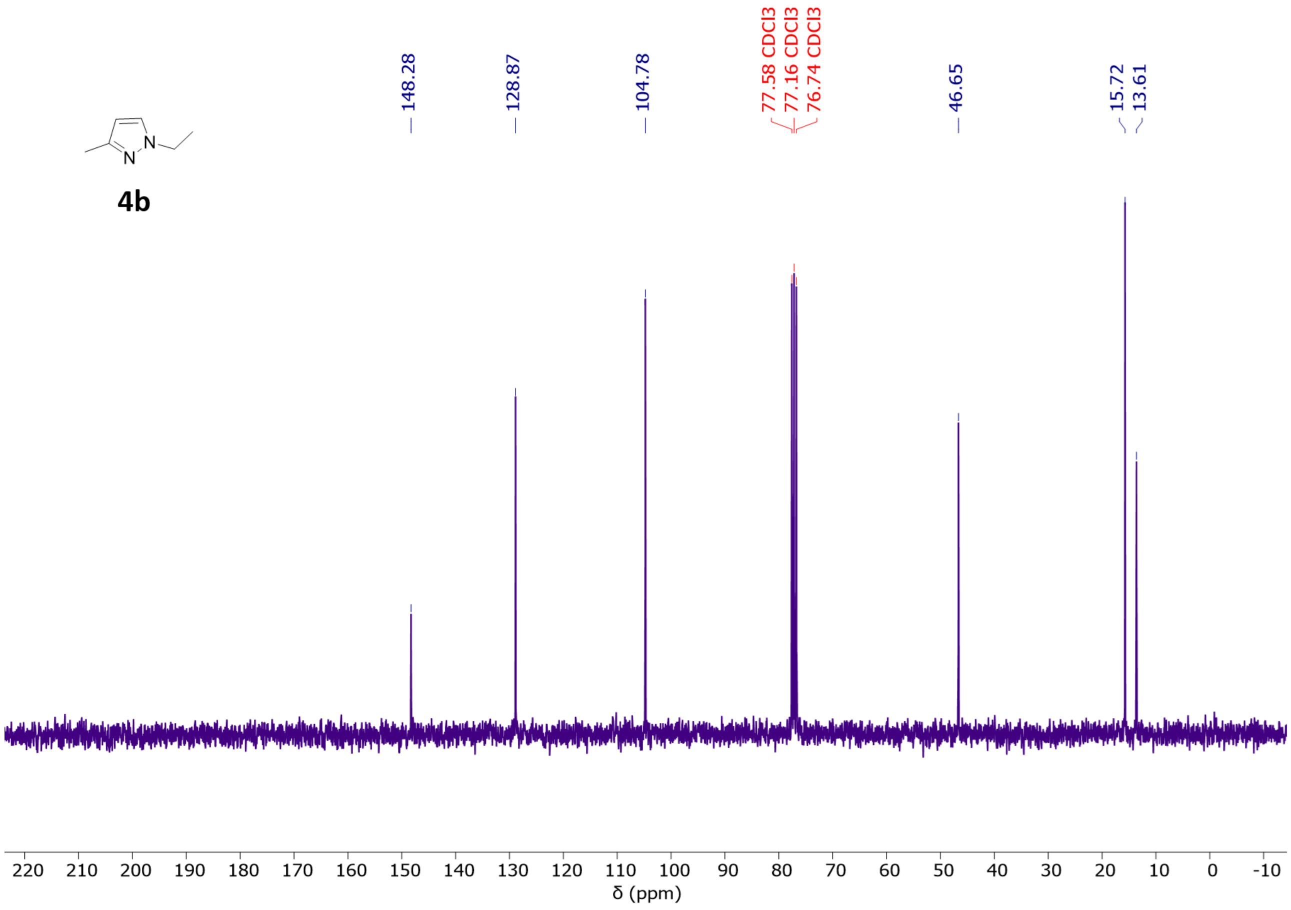

Figure S56. ^{13}C {^{1}H} NMR spectrum of compound 1-ethyl-3-methyl-*1H*-pyrazole.

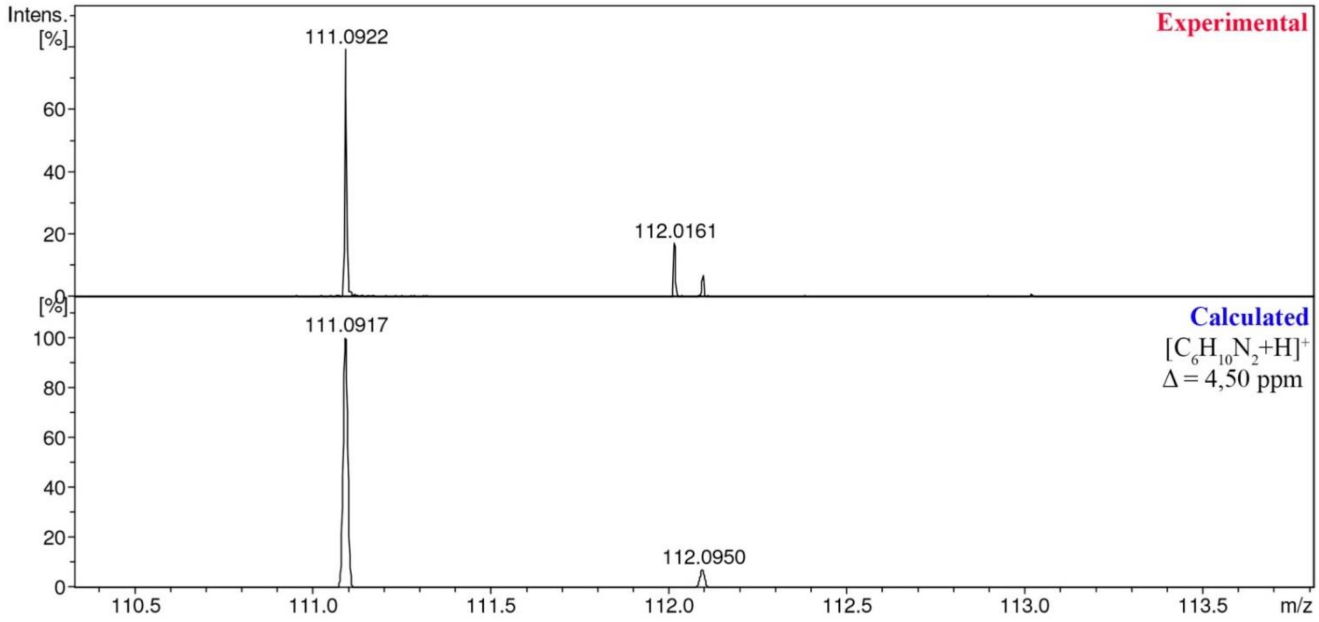

Figure S57. ESI-HRMS of compound 1-ethyl-3-methyl-*1H*-pyrazole.

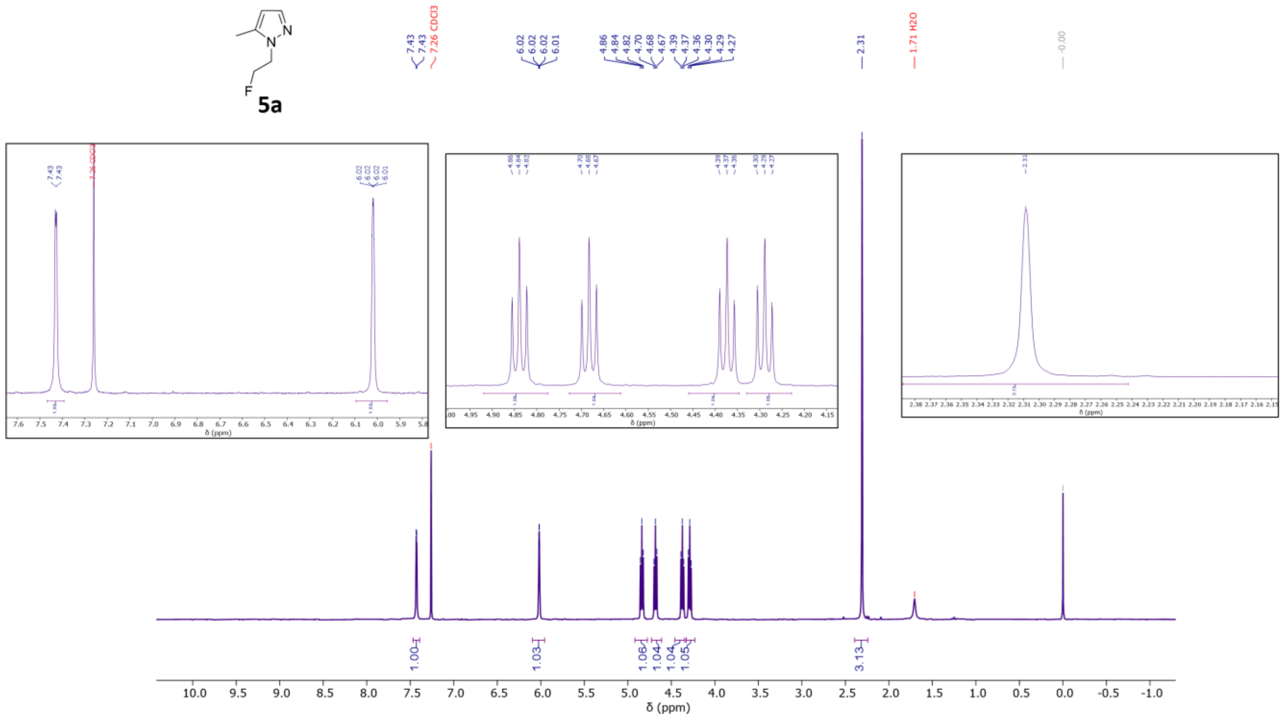

Figure S58. ^{1}H NMR spectrum of compound 1-(2-Fluoroethyl)-5-methyl-*1H*-pyrazole.

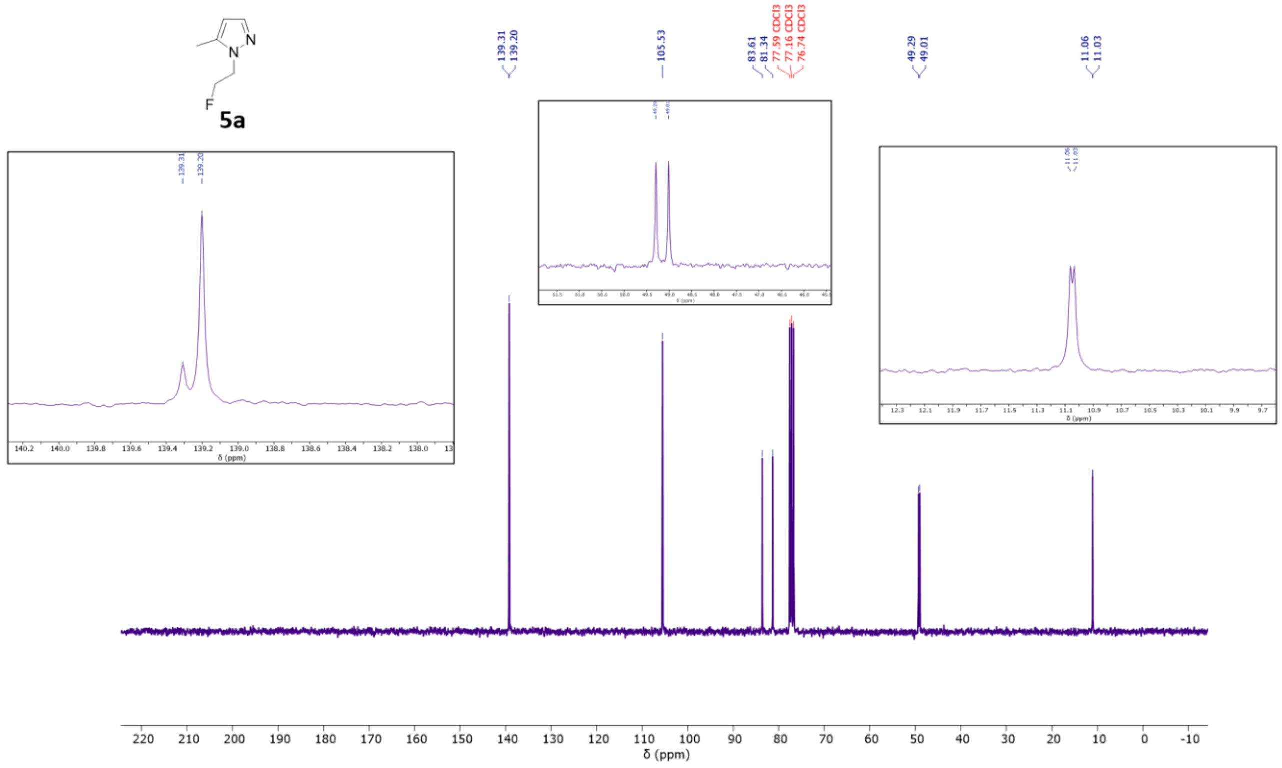

Figure S59. ^{13}C {^{1}H} NMR spectrum of compound 1-(2-fluoroethyl)-5-methyl-*1H*-pyrazole.

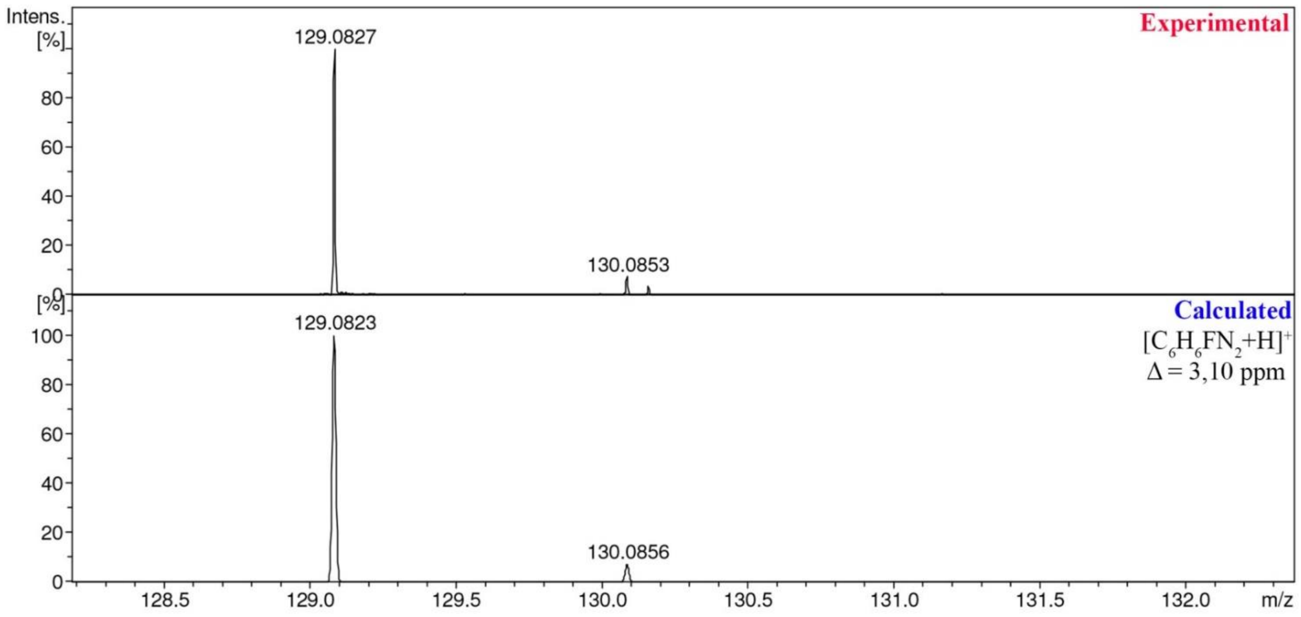

Figure S60. ESI-HRMS of the compound 1-(2-fluoroethyl)-5-methyl-*1H*-pyrazole.

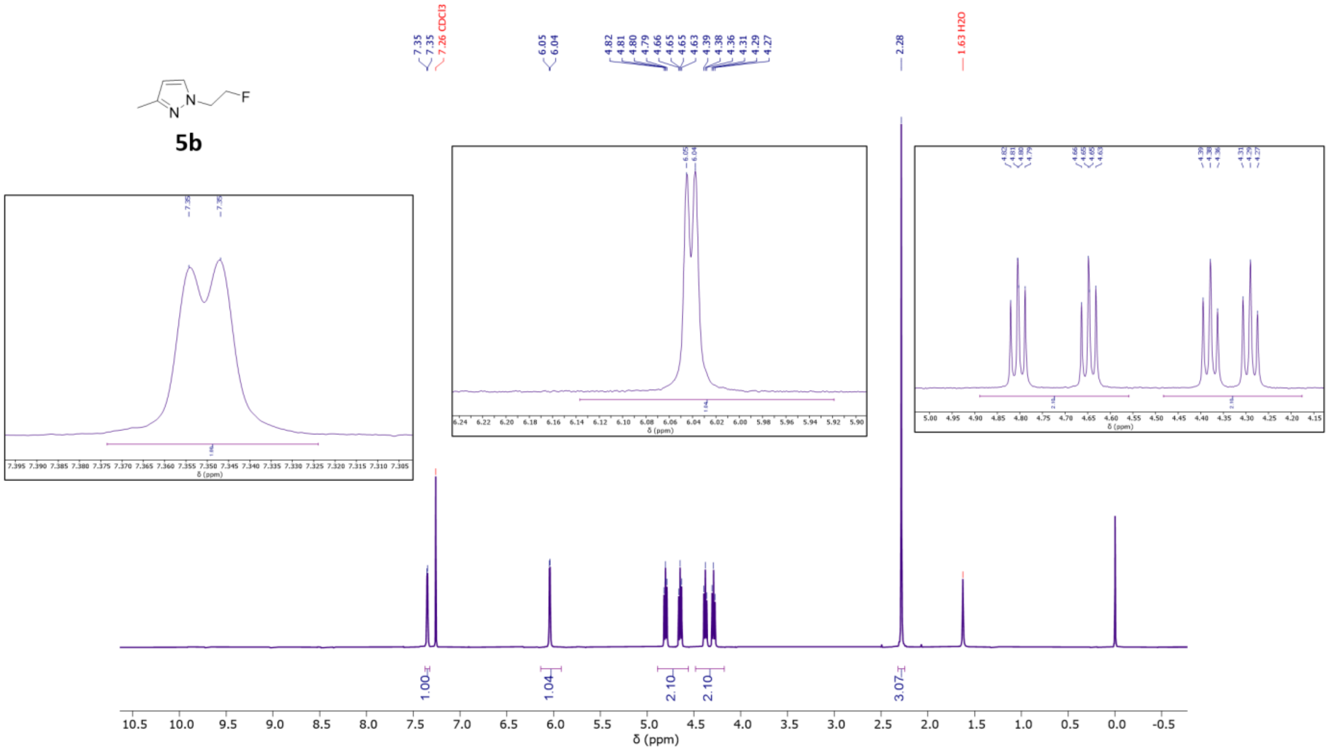

Figure S61. ^{1}H NMR spectrum of the compound 1-(2-fluoroethyl)-3-methyl-*1H*-pyrazole.

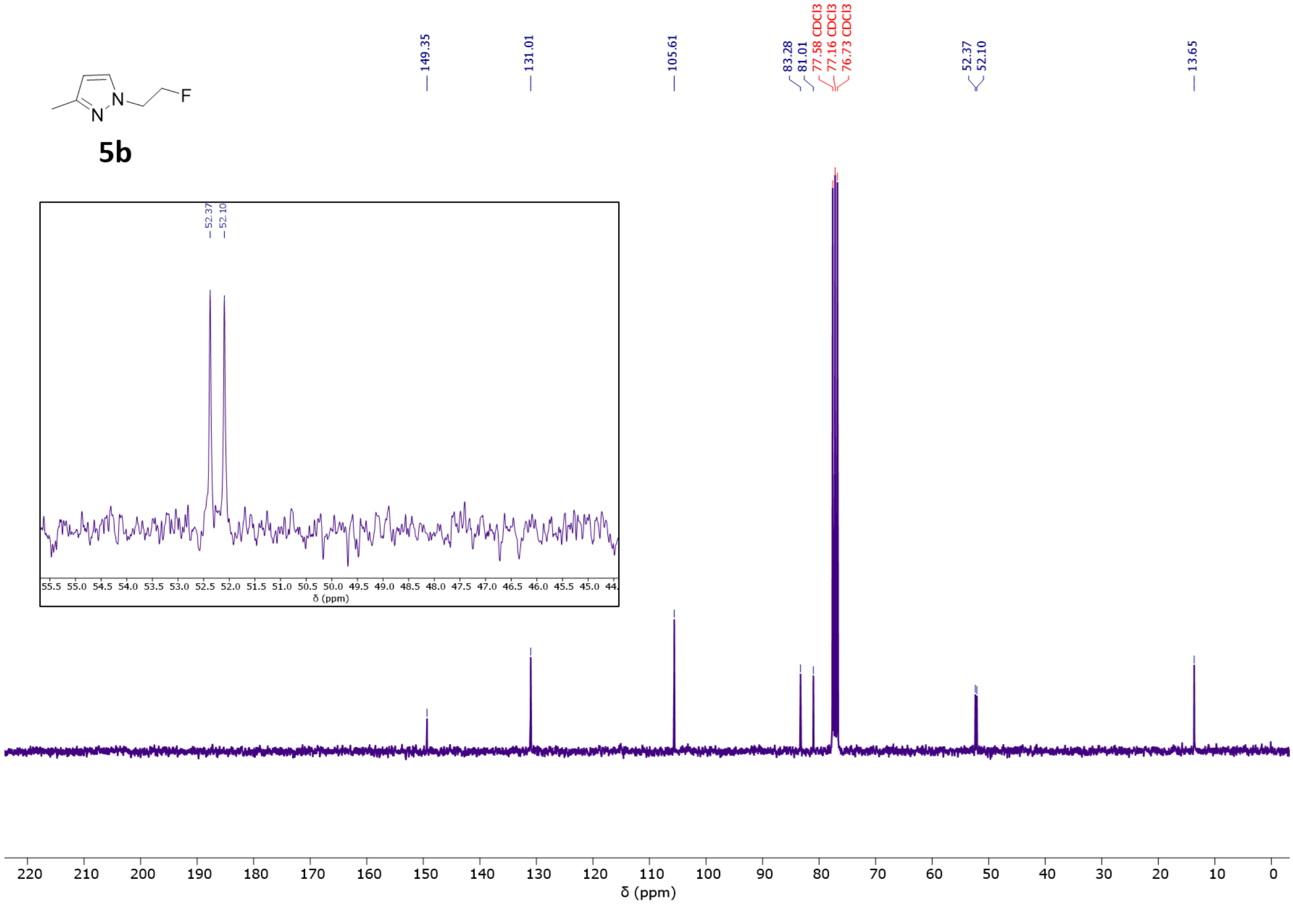

Figure S62. ^{13}C {^{1}H} NMR spectrum of compound 1-(2-fluoroethyl)-3-methyl-*1H*-pyrazole.

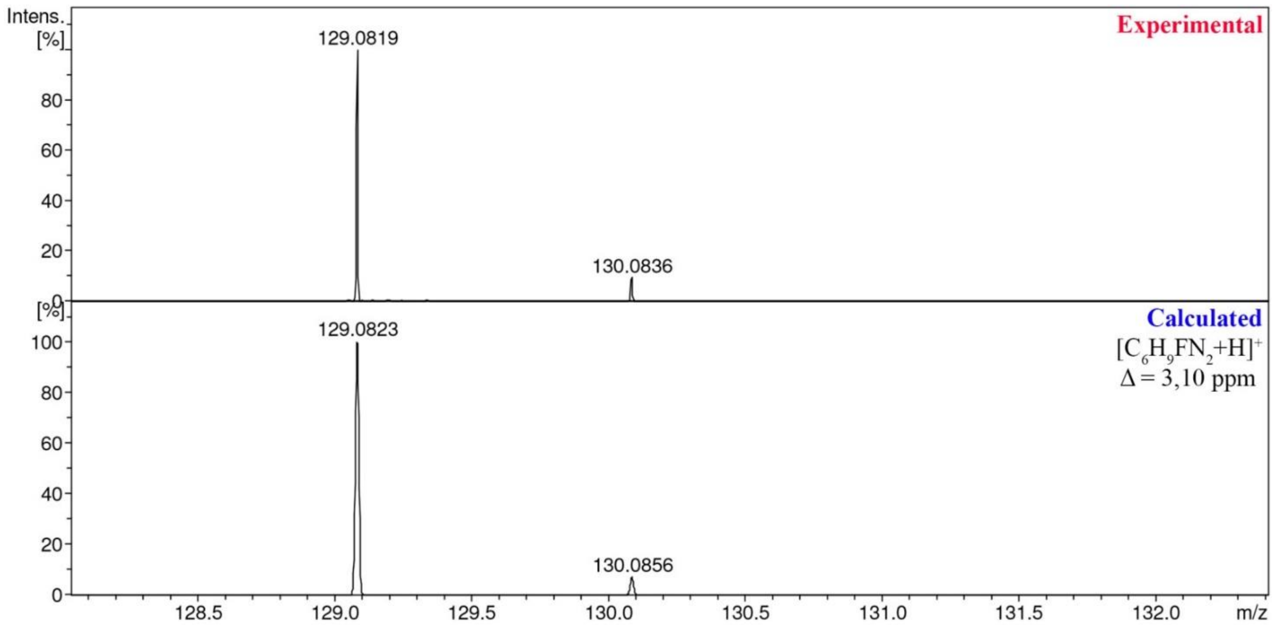

Figure S63. ESI-HRMS of the compound 1-(2-fluoroethyl)-3-methyl-*1H*-pyrazole.

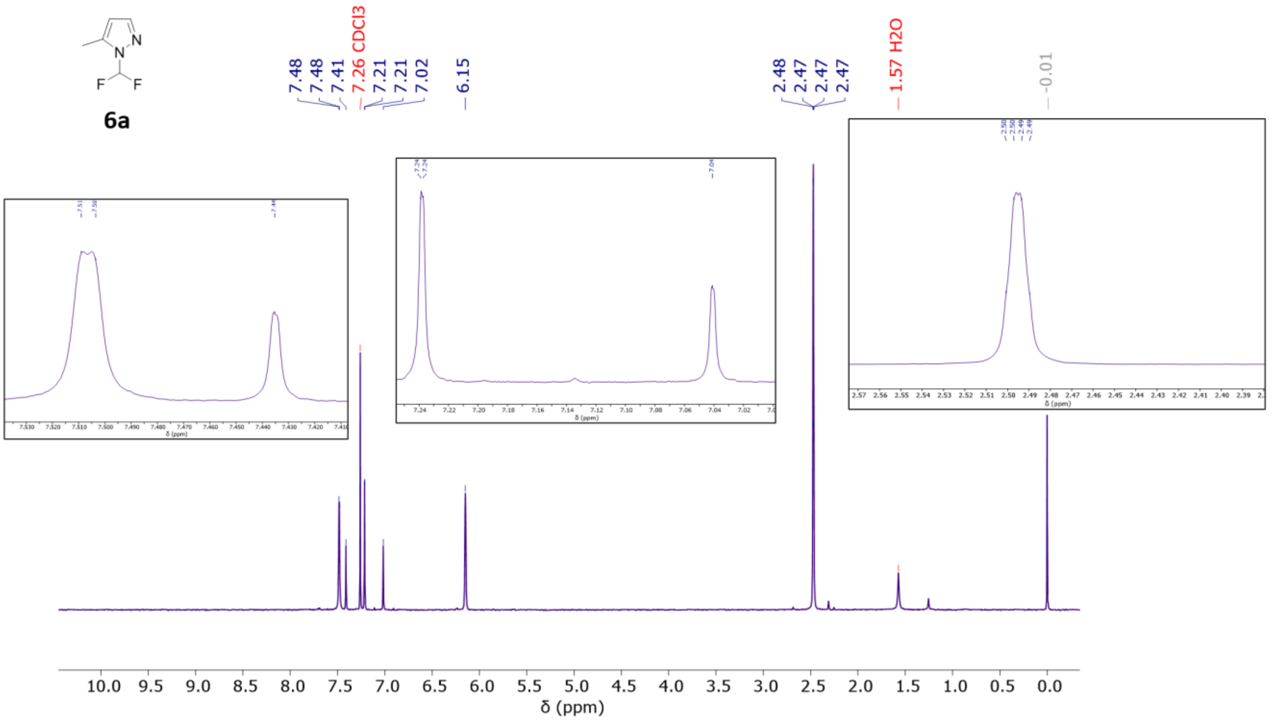

Figure S64. ^{1}H NMR spectrum of compound 1-(difluoromethyl)-5-methyl-*1H*-pyrazole.

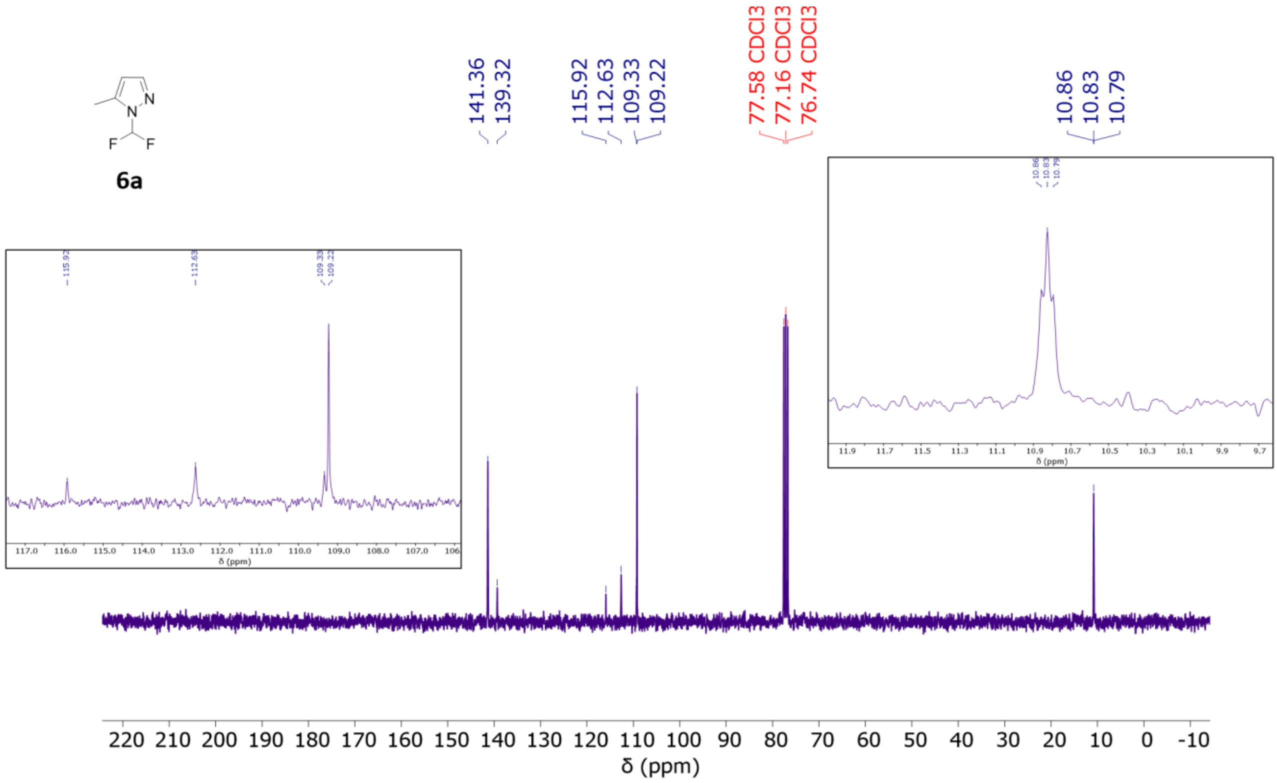

Figure S65. ^{13}C {^{1}H} NMR spectrum of compound 1-(difluoromethyl)-5-methyl-*1H*-pyrazole.

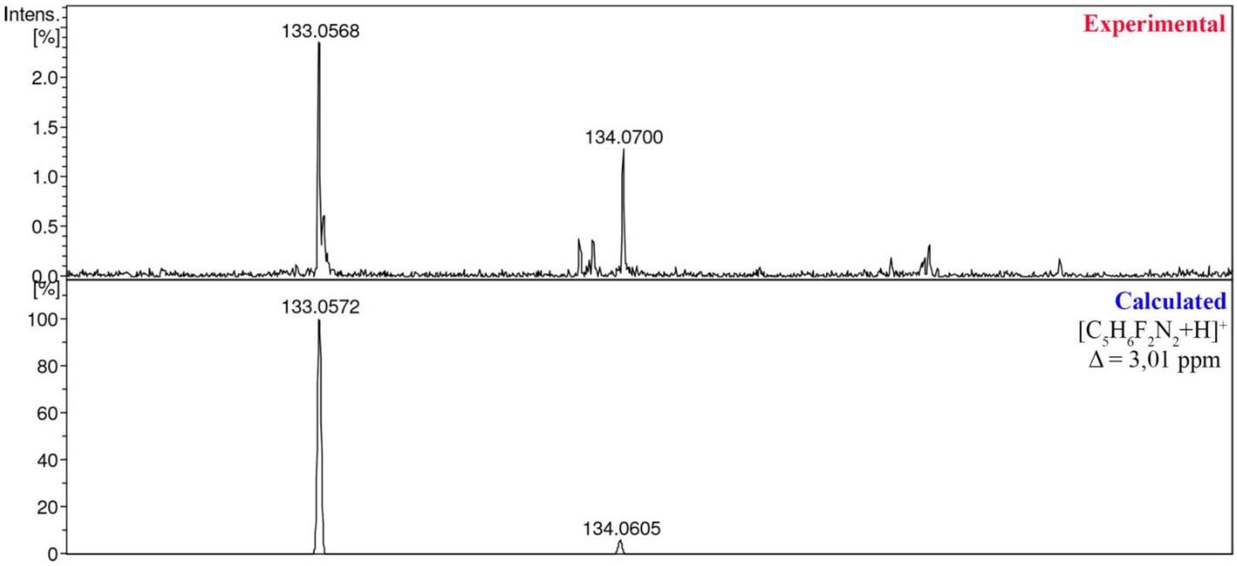

Figure S66. ESI-HRMS of the compound 1-(difluoromethyl)-5-methyl-*1H*-pyrazole.

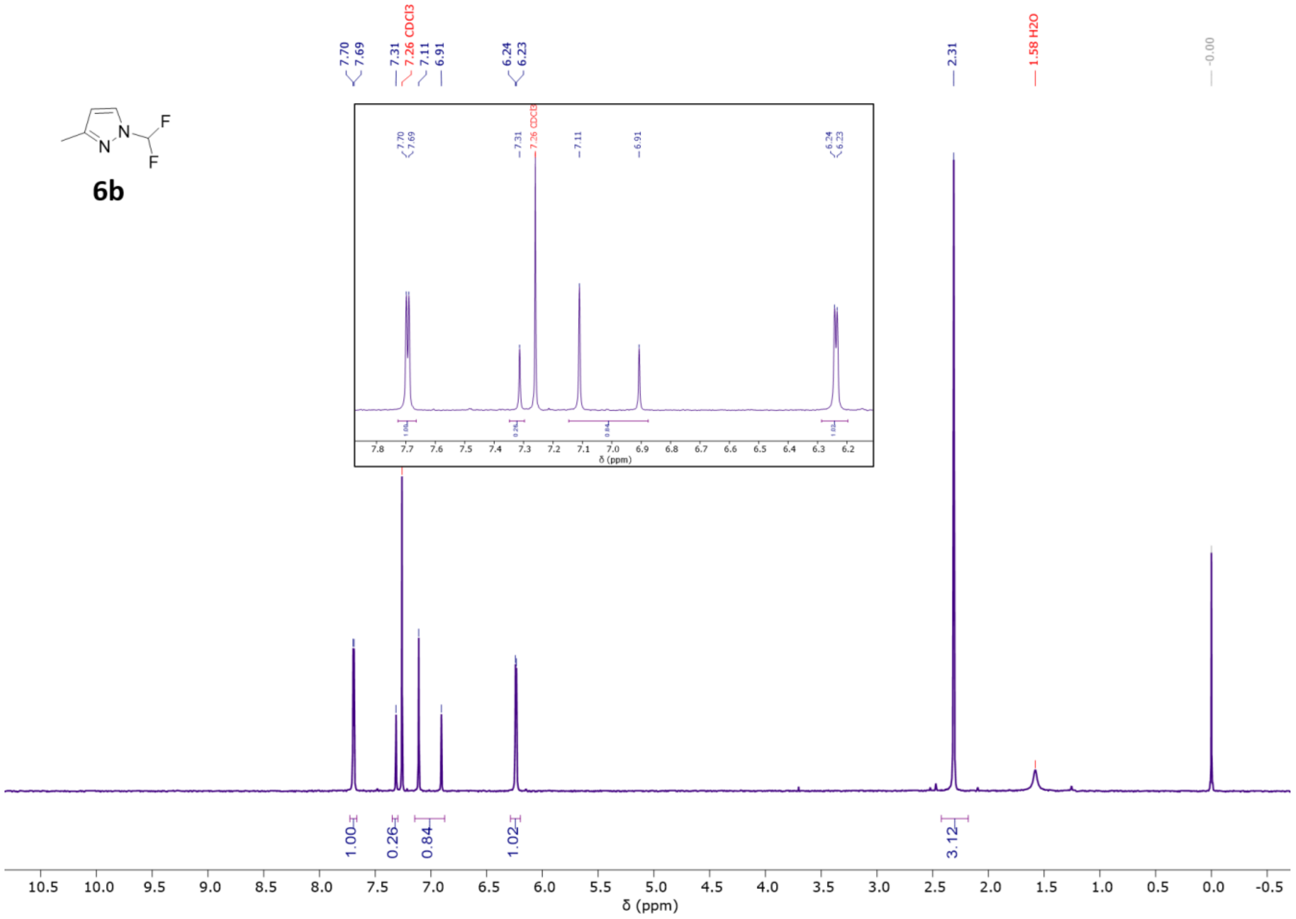

Figure S67. ^{1}H NMR spectrum of compound 1-(difluoromethyl)-3-methyl-*1H*-pyrazole.

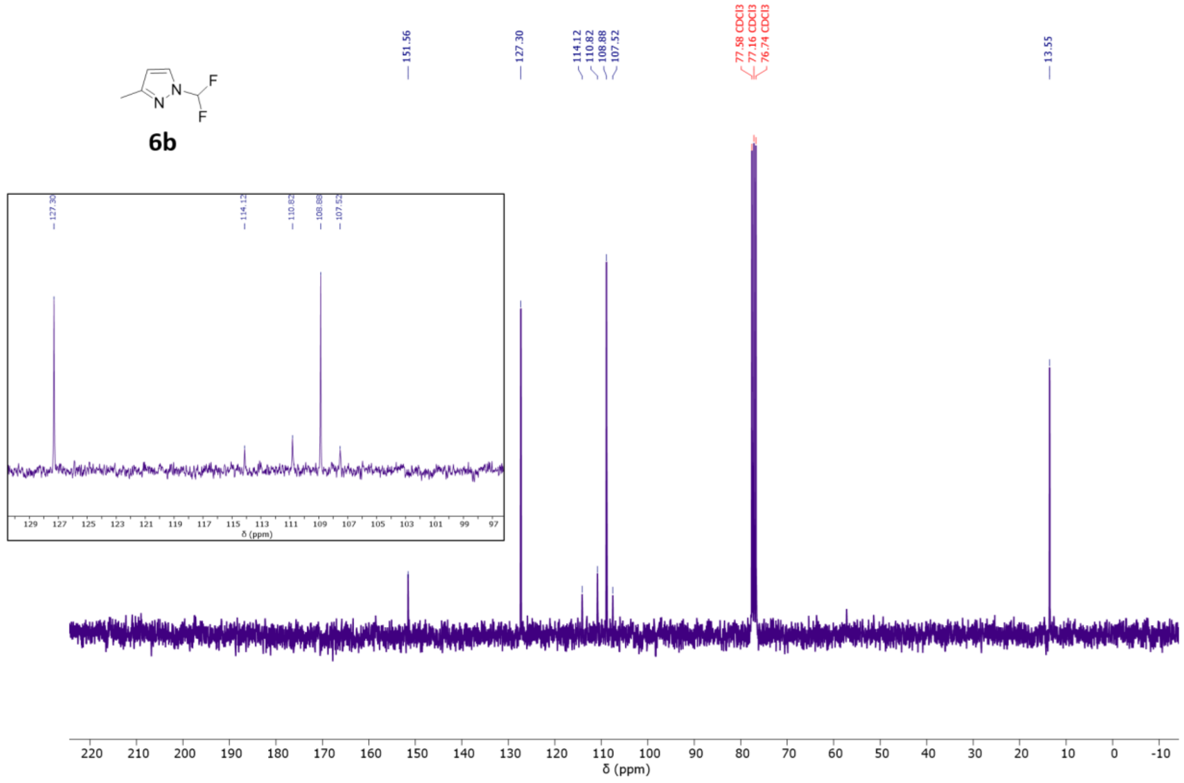

Figure S68. ^{13}C {^{1}H} NMR spectrum of compound 1-(difluoromethyl)-3-methyl-*1H*-pyrazole.

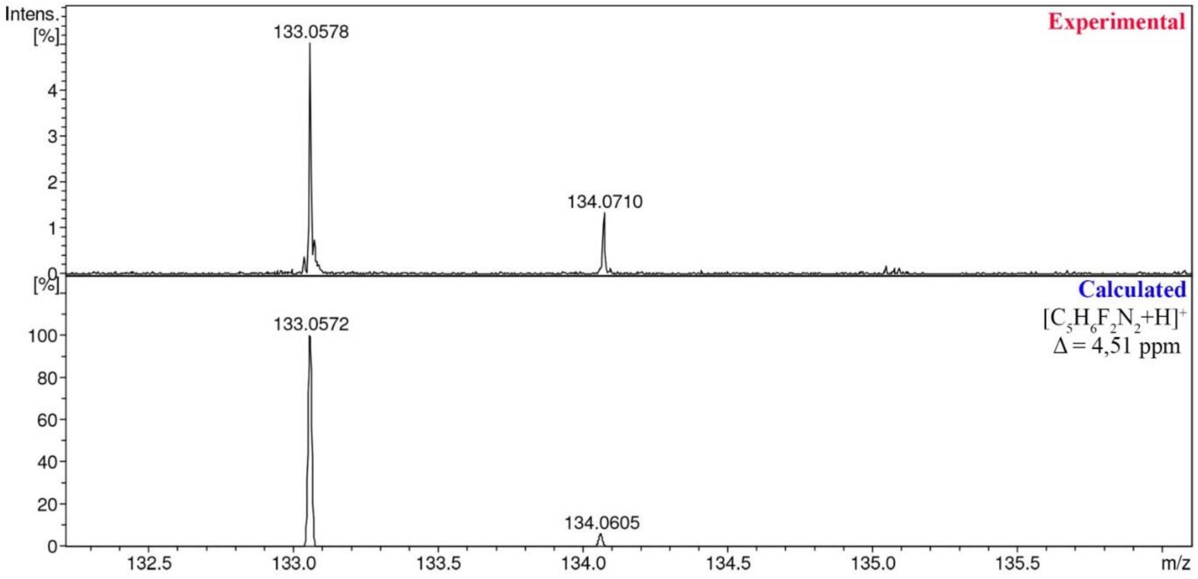

Figure S69. ESI-HRMS of the compound 1-(difluoromethyl)-3-methyl-1H-pyrazole.

X-ray crystallographic data and refinement details.

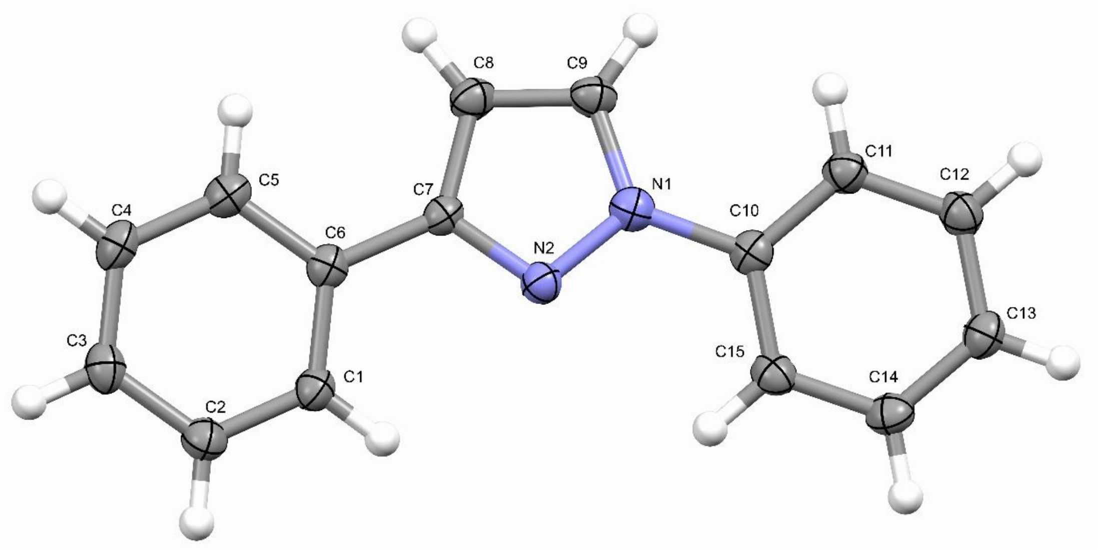

Figure S70. X-ray crystallographic images of 1,3-diphenyl-*1H*-pyrazole.

Table S1. Crystal data and structure refinement for 1,3-diphenyl-1H-pyrazole.

Empirical formula	for C15 H12 N2	
Formula weight	220.27	
Temperature	100.00(10) K	
Wavelength	1.54184 Å	
Crystal system	Monoclinic	
Space group	$P2_1/c$	
Unit cell dimensions	a = 5.55130(10) Å	α= 90°.
	b = 9.08050(10) Å	β= 93.8320(10)°.
	c = 22.6176(3) Å	γ = 90°.
Volume	1137.57(3) $Å^3$	
Z	4	
Density (calculated)	1.286 g/cm^3	
Absorption coefficient	0.599 mm^{-1}	
F(000)	464	
Crystal size	0.31 × 0.17 × 0.04 mm^3	
Theta range for data collection	3.918-79.783°.	
Index ranges	-6<=h<=7, -11<=k<=7, -28<=l<=28	
Reflections collected	13169	
Independent reflections	2471 [R(int) = 0.0266]	
Observed reflections	2305	
Completeness to theta = 67.684°	100.0%	
Absorption correction	Gaussian	
Max. and min. transmission	1.000 and 0.512	
Refinement method	Full-matrix least-squares on F^2	
Data/restraints/parameters	2471/0/155	
Goodness-of-fit on F^2	1.030	
Final R indices [I>2 sigma(I)]	R1 = 0.0397, wR2 = 0.1100	
R indices (all data)	R1 = 0.0420, wR2 = 0.1123	
Extinction coefficient	0.0020(5)	
Largest diff. peak and hole	0.391 and -0.253 e.$Å^{-3}$	

Table S2. Atomic coordinates (x 10^4) and equivalent isotropic displacement parameters ($Å^2$x 10^3) for 1,3-diphenyl-1H-pyrazole. U(eq) is defined as one third of the trace of the orthogonalized U^{ij} tensor.

	x	y	z	U(eq)
N(1)	3824(2)	5084(1)	7278(1)	26(1)
N(2)	3444(2)	5995(1)	6806(1)	24(1)
C(1)	3251(2)	7245(1)	5656(1)	24(1)
C(2)	3301(2)	7945(1)	5111(1)	28(1)
C(3)	5178(2)	7683(1)	4748(1)	28(1)
C(4)	7023(2)	6730(1)	4938(1)	28(1)
C(5)	7002(2)	6037(1)	5484(1)	26(1)
C(6)	5098(2)	6280(1)	5847(1)	23(1)
C(7)	5021(2)	5545(1)	6415(1)	21(1)
C(8)	6393(2)	4353(1)	6640(1)	27(1)
C(9)	5612(2)	4076(1)	7189(1)	26(1)
C(10)	2419(2)	5246(1)	7785(1)	23(1)
C(11)	3116(2)	4505(1)	8307(1)	26(1)
C(12)	1769(2)	4655(1)	8799(1)	28(1)
C(13)	-268(2)	5550(1)	8773(1)	27(1)
C(14)	-949(2)	6293(1)	8252(1)	28(1)
C(15)	376(2)	6144(1)	7756(1)	26(1)

Table S3. Bond lengths [Å] and angles [°] for 1,3-diphenyl-1H-pyrazole.

N(1)-N(2)	1.3555(14)
N(1)-C(9)	1.3750(16)
N(1)-C(10)	1.4368(15)
N(2)-C(7)	1.3482(14)
C(1)-H(1)	0.9500
C(1)-C(2)	1.3884(17)
C(1)-C(6)	1.3950(17)
C(2)-H(2)	0.9500
C(2)-C(3)	1.3903(17)
C(3)-H(3)	0.9500
C(3)-C(4)	1.3874(19)
C(4)-H(4)	0.9500
C(4)-C(5)	1.3875(17)
C(5)-H(5)	0.9500
C(5)-C(6)	1.3979(16)
C(6)-C(7)	1.4518(16)
C(7)-C(8)	1.3996(16)
C(8)-H(8)	0.9500
C(8)-C(9)	1.3656(17)
C(9)-H(9)	0.9500
C(10)-C(11)	1.3925(17)
C(10)-C(15)	1.3947(17)
C(11)-H(11)	0.9500
C(11)-C(12)	1.3868(17)
C(12)-H(12)	0.9500
C(12)-C(13)	1.3911(18)
C(13)-H(13)	0.9500
C(13)-C(14)	1.3881(18)
C(14)-H(14)	0.9500
C(14)-C(15)	1.3891(17)
C(15)-H(15)	0.9500
N(2)-N(1)-C(9)	111.56(10)
N(2)-N(1)-C(10)	120.12(10)
C(9)-N(1)-C(10)	128.31(10)
C(7)-N(2)-N(1)	104.93(9)
C(2)-C(1)-H(1)	119.8
C(2)-C(1)-C(6)	120.33(11)
C(6)-C(1)-H(1)	119.8
C(1)-C(2)-H(2)	119.8
C(1)-C(2)-C(3)	120.42(12)
C(3)-C(2)-H(2)	119.8
C(2)-C(3)-H(3)	120.3
C(4)-C(3)-C(2)	119.36(11)
C(4)-C(3)-H(3)	120.3
C(3)-C(4)-H(4)	119.7
C(3)-C(4)-C(5)	120.63(11)
C(5)-C(4)-H(4)	119.7
C(4)-C(5)-H(5)	119.9

C(4)-C(5)-C(6)	120.16(11)
C(6)-C(5)-H(5)	119.9
C(1)-C(6)-C(5)	119.10(11)
C(1)-C(6)-C(7)	119.94(10)
C(5)-C(6)-C(7)	120.97(11)
N(2)-C(7)-C(6)	119.97(10)
N(2)-C(7)-C(8)	110.93(10)
C(8)-C(7)-C(6)	129.10(11)
C(7)-C(8)-H(8)	127.0
C(9)-C(8)-C(7)	105.98(11)
C(9)-C(8)-H(8)	127.0
N(1)-C(9)-H(9)	126.7
C(8)-C(9)-N(1)	106.60(11)
C(8)-C(9)-H(9)	126.7
C(11)-C(10)-N(1)	119.55(11)
C(11)-C(10)-C(15)	120.08(11)
C(15)-C(10)-N(1)	120.37(11)
C(10)-C(11)-H(11)	120.0
C(12)-C(11)-C(10)	119.92(11)
C(12)-C(11)-H(11)	120.0
C(11)-C(12)-H(12)	119.9
C(11)-C(12)-C(13)	120.28(11)
C(13)-C(12)-H(12)	119.9
C(12)-C(13)-H(13)	120.2
C(14)-C(13)-C(12)	119.61(11)
C(14)-C(13)-H(13)	120.2
C(13)-C(14)-H(14)	119.7
C(13)-C(14)-C(15)	120.62(11)
C(15)-C(14)-H(14)	119.7
C(10)-C(15)-H(15)	120.3
C(14)-C(15)-C(10)	119.49(11)
C(14)-C(15)-H(15)	120.3

Symmetry transformations used to generate equivalent atoms:

Table S4. Anisotropic displacement parameters ($Å^2 x 10^3$) for 1,3-diphenyl-1H-pyrazole. The anisotropic

The displacement factor exponent takes the form: $-2\pi^2[h^2 a^{*2}U^{11} + ... + 2 h k a^* b^* U^{12}]$

	U^{11}	U^{22}	U^{33}	U^{23}	U^{13}	U^{12}
N(1)	26(1)	24(1)	26(1)	-1(1)	1(1)	-1(1)
N(2)	24(1)	22(1)	24(1)	1(1)	3(1)	0(1)
C(1)	22(1)	28(1)	24(1)	-2(1)	3(1)	-1(1)
C(2)	25(1)	31(1)	26(1)	1(1)	-1(1)	1(1)
C(3)	29(1)	31(1)	24(1)	-1(1)	3(1)	-6(1)
C(4)	26(1)	30(1)	28(1)	-5(1)	8(1)	-4(1)
C(5)	23(1)	25(1)	30(1)	-4(1)	4(1)	-1(1)
C(6)	23(1)	22(1)	23(1)	-4(1)	1(1)	-3(1)
C(7)	19(1)	20(1)	22(1)	-4(1)	1(1)	-1(1)
C(8)	27(1)	26(1)	28(1)	-4(1)	2(1)	3(1)
C(9)	27(1)	22(1)	29(1)	-1(1)	-1(1)	3(1)
C(10)	23(1)	20(1)	26(1)	-1(1)	2(1)	-4(1)
C(11)	26(1)	24(1)	29(1)	1(1)	3(1)	1(1)
C(12)	31(1)	26(1)	26(1)	4(1)	3(1)	0(1)
C(13)	25(1)	29(1)	28(1)	-1(1)	6(1)	-3(1)
C(14)	22(1)	28(1)	33(1)	1(1)	2(1)	0(1)
C(15)	23(1)	26(1)	27(1)	3(1)	-1(1)	-2(1)

Table S5. Hydrogen coordinates (x 10^4) and isotropic displacement parameters (Å^{2}x 10 ^{3}) for 1,3-diphenyl-1H-pyrazole.

	x	y	z	U(eq)
H(1)	1952	7423	5899	29
H(2)	2044	8608	4986	33
H(3)	5198	8153	4372	33
H(4)	8313	6550	4692	34
H(5)	8286	5397	5613	31
H(8)	7618	3841	6450	32
H(9)	6192	3332	7458	31
H(11)	4512	3897	8327	32
H(12)	2240	4143	9154	33
H(13)	-1187	5653	9110	33
H(14)	-2335	6910	8235	33
H(15)	-106	6649	7400	31

Table S6. Torsion angles [°] for 1,3-diphenyl-1H-pyrazole.

N(1)-N(2)-C(7)-C(6)	179.43(9)
N(1)-N(2)-C(7)-C(8)	-0.01(13)
N(1)-C(10)-C(11)-C(12)	-179.96(11)
N(1)-C(10)-C(15)-C(14)	-179.60(10)
N(2)-N(1)-C(9)-C(8)	-0.19(14)
N(2)-N(1)-C(10)-C(11)	-167.86(10)
N(2)-N(1)-C(10)-C(15)	11.89(16)
N(2)-C(7)-C(8)-C(9)	-0.10(14)
C(1)-C(2)-C(3)-C(4)	-0.91(19)
C(1)-C(6)-C(7)-N(2)	-12.69(16)
C(1)-C(6)-C(7)-C(8)	166.65(12)
C(2)-C(1)-C(6)-C(5)	0.40(17)
C(2)-C(1)-C(6)-C(7)	-179.56(11)
C(2)-C(3)-C(4)-C(5)	0.19(18)
C(3)-C(4)-C(5)-C(6)	0.83(18)
C(4)-C(5)-C(6)-C(1)	-1.11(17)
C(4)-C(5)-C(6)-C(7)	178.85(10)
C(5)-C(6)-C(7)-N(2)	167.35(11)
C(5)-C(6)-C(7)-C(8)	-13.31(18)
C(6)-C(1)-C(2)-C(3)	0.61(18)
C(6)-C(7)-C(8)-C(9)	-179.48(11)
C(7)-C(8)-C(9)-N(1)	0.17(13)
C(9)-N(1)-N(2)-C(7)	0.12(13)
C(9)-N(1)-C(10)-C(11)	12.41(18)
C(9)-N(1)-C(10)-C(15)	-167.84(11)
C(10)-N(1)-N(2)-C(7)	-179.65(10)
C(10)-N(1)-C(9)-C(8)	179.56(11)
C(10)-C(11)-C(12)-C(13)	-0.41(19)
C(11)-C(10)-C(15)-C(14)	0.15(18)
C(11)-C(12)-C(13)-C(14)	0.10(19)
C(12)-C(13)-C(14)-C(15)	0.35(19)
C(13)-C(14)-C(15)-C(10)	-0.47(18)
C(15)-C(10)-C(11)-C(12)	0.29(18)

References

[1] J. P. Perdew, K. Burke, M. Ernzerhof, *Phys. Rev. Lett.,* 1996, **77**, 3865-3868.
[2] C. Adamo, V. Barone, *J. Chem. Phys.*, 1999, **110**, 6158-6170.
[3] F. Weigend, R. Ahlrichs, *Phys. Chem. Chem. Phys.,* 2005, **7**, 3297-3305.
[4] F. Weigend, *Phys. Chem. Chem. Phys.,* 2006, **8**, 1057- 1065.
[5] S. Grimme, S. Ehrlich, L. Goerigk, *J. Comput. Chem.,* 2011, **32**, 1456.
[6] S. Grimme, J. Antony, S. Ehrlich, H. Krieg, *J. Chem. Phys.*, 2010, **132**, 154104.
[7] M. J. Frisch, G. W. Trucks, H. B. Schlegel, G. E. Scuseria, M. A. Robb, J. R. Cheeseman, G. Scalmani, V. Barone, G. A. Petersson, H. Nakatsuji, X. Li, M. Caricato, A. V. Marenich, J. Bloino, B. G. Janesko, R. Gomperts, B. Mennucci, H. P. Hratchian, J. V. Ortiz, A. F. Izmaylov, J. L. Sonnenberg, D. F. Williams-Young, Ding, F. Lipparini, F. Egidi, J. Goings, B. Peng, A. Petrone, T. Henderson, D. Ranasinghe, V. G. Zakrzewski, J. Gao, N. Rega, G. Zheng, W. Liang, M. Hada, M. Ehara, K. Toyota, R. Fukuda, J. Hasegawa, M. Ishida, T. Nakajima, Y. Honda, O. Kitao, H. Nakai, T. Vreven, K. Throssell, J. A., Jr. Montgomery, J. E. Peralta, F. Ogliaro, M. J. Bearpark, J. J. Heyd, E. N. Brothers, K. N. Kudin, V. N. Staroverov, T. A. Keith, R. Kobayashi, J. Normand, K. Raghavachari, A. P. Rendell, J. C. Burant, S. S. Iyengar, J. Tomasi, M. Cossi, J. M. Millam, M. Klene, C. Adamo, R. Cammi, J. W. Ochterski, R. L. Martin, K. Morokuma, O. Farkas, J. B. Foresman, D. J. Fox, Gaussian 16 Revision C.01, Inc., Wallingford CT, 2016.
[8] V. Marenich, C. J. Cramer, D. G. Truhlar, *J. Phys. Chem. B.*, 2009, **113**, 6378-6396.
[9] C. Y. Legault, CYLview20; Université de Sherbrooke, 2020. http://www.cylview.org.
[10] CrysAlisPro. Version 1.171.41.106a. *Rigaku Oxford Diffraction*, **2021**.
[11] G. M. Sheldrick, *Acta Cryst.,* 2015, **A71**, 1, 3-8.
[12] G. M. Sheldrick, *Acta Cryst.,* 2015, **C71**, 1, 3-8.
[13] O. V. Dolomanov, L. J. Bourhis, R. J. Gildea, J. A. K. Howard, H. Puschmann, *J. Appl. Cryst.* 2009, **42**, 2, 339-341.
[14] C. F. Macrae, I. Sovago, S. J. Cottrell, P. T. A. Galek, P. McCabe, E. Pidcock, M. Platings, G. P. Shields, J. S. Stevens, M. Towler, P. A. Wood, *J. Appl. Cryst.,* 2020, **53**, 226-235.
[15] R. SanMartin, E. Martínez de Marigorta, E. Domínguez, *Tetrahedron,* 1994, **50**, 2255-2264.
[16] R. Olivera, R. SanMartin, E. Domínguez, *J. Org. Chem.,* 2000, **65**, 7010-7019.
[17] S. Hernández, I. l Moreno, R. SanMartin, G. Gómez, M. T. Herrero, E. Domínguez. *J. Org. Chem.,* 2010, **75**, 2, 434-441.
